# Role of Phosphorylated Gonadotropin-Regulated Testicular RNA Helicase (GRTH/DDX25) in the Regulation of Germ Cell Specific mRNAs in Chromatoid Bodies During Spermatogenesis

**DOI:** 10.3389/fcell.2020.580019

**Published:** 2020-12-23

**Authors:** Rajakumar Anbazhagan, Raghuveer Kavarthapu, Steven L. Coon, Maria L. Dufau

**Affiliations:** ^1^Section on Molecular Endocrinology, Division of Developmental Biology, Eunice Kennedy Shriver National Institute of Child Health and Human Development, National Institutes of Health, Bethesda, MD, United States; ^2^Molecular Genomics Core, Eunice Kennedy Shriver National Institute of Child Health and Human Development, National Institutes of Health, Bethesda, MD, United States

**Keywords:** phospho-GRTH, chromatoid bodies, spermatogenesis, transcriptome analysis, TP2 and PRM2

## Abstract

GRTH/DDX25 is a member of the DEAD-box family of RNA helicases that play an essential role in spermatogenesis. GRTH knock-in (KI) mice with the human mutant GRTH gene (R242H) show loss of the phospho-species from cytoplasm with preservation of the non-phospho form in the cytoplasm and nucleus. GRTH KI mice are sterile and lack elongated spermatids and spermatozoa, with spermatogenic arrest at step 8 of round spermatids which contain chromatoid body (CB) markedly reduced in size. We observed an absence of phospho-GRTH in CB of GRTH KI mice. RNA-Seq analysis of mRNA isolated from CB revealed that 1,421 genes show differential abundance, of which 947 genes showed a decrease in abundance and 474 genes showed an increase in abundance in GRTH KI mice. The transcripts related to spermatid development, differentiation, and chromatin remodeling (*Tnp1/2*, *Prm1/2/3*, *Spem1/2*, *Tssk 2/3/6*, *Grth*, *tAce*, and *Upf2*) were reduced, and the transcripts encoding for factors involved in RNA transport, regulation, and surveillance and transcriptional and translational regulation (*Eef1a1, Ppp1cc*, *Pabpc1*, *Ybx3*, *Tent5b*, *H2al1m*, *Dctn2*, and *Dync1h1*) were increased in the CB of KI mice and were further validated by qPCR. In the round spermatids of wild-type mice, mRNAs of *Tnp2*, *Prm2*, and *Grth* were abundantly co-localized with MVH protein in the CB, while in GRTH KI mice these were minimally present. In addition, GRTH binding to *Tnp1/2*, *Prm1/2*, *Grth*, and *Tssk6* mRNAs was found to be markedly decreased in KI. These results demonstrate the importance of phospho-GRTH in the maintenance of the structure of CB and its role in the storage and stability of germ cell-specific mRNAs during spermiogenesis.

## Introduction

Spermatogenesis is characterized by a complex and highly specialized differentiation program, in which male germ cells show a cohesive expression of an array of testicular genes with a remarkable degree of cellular restructuring involved in a complex events of genomic and epigenetic reorganization to produce mature spermatozoa (Steger, 2001; Sassone-Corsi, 2002; Kimmins and Sassone-Corsi, 2005). During these events, several mRNAs transported from the nucleus to the cytoplasm undergo near-to-complete translational silencing/repression, extensive post-transcriptional processing, and storage at specific cytoplasmic sites, such as the ribonucleoprotein (RNP) granules called chromatoid bodies (CBs), for translation at later stages of spermiogenesis (Steger, 2001; Maclean and Wilkinson, 2005; Kotaja and Sassone-Corsi, 2007). Owing to the importance of accurate RNA regulatory mechanisms in controlling normal spermatogenesis, it is imperative to study the functions of CB which may act as a compartment for mRNA processing pathways.

The CB is a membraneless, perinuclear organelle which primarily contains Piwi-interacting RNA (piRNA), mRNAs, small RNAs, long non-coding RNAs, RNA binding proteins, and other proteins involved in RNA processing of male germ cells which play a critical role in chromatin compaction during sperm elongation (Parvinen, 2005; Sato et al., 2010). CBs temporarily store mRNAs that are transported from the nucleus by gonadotropin-regulated testicular RNA helicase (GRTH/DDX25), where these are translationally repressed, awaiting translation at various stages of spermatid development, and the mRNAs can also undergo degradation (Sheng et al., 2006; Sato et al., 2010). During spermiogenesis, there is a precise control and stability in the quality of transcription and posttranscriptional regulation to secure the correct timing of protein expression in male germ cells/spermatids (Sato et al., 2010). At later stages of spermatid elongation, “CB” splits into two separate structures: one gets discarded along with the residual cytoplasm, and the other forms a ring around the base of the flagellum (Fawcett et al., 1970; Shang et al., 2010; Meikar et al., 2011; Lehtiniemi and Kotaja, 2018). The origin of CBs is highly debatable, the most accepted notion being their emergence from small granules which are associated with the nuclear envelope present nearby the inter-mitochondrial cement (IMC). The IMC is highly electron-dense and has CB-like material found among clusters of mitochondria during male germ cell differentiation (Fawcett et al., 1970; Yokota, 2012; Lehtiniemi and Kotaja, 2018), and it is particularly evident in pachytene spermatocytes. CBs are believed to possess functional similarities to RNA processing bodies (P-bodies) (Franks and Lykke-Andersen, 2008; Luo et al., 2018) and stress granules of somatic cells that maintain RNA regulation (Eulalio et al., 2007; Decker and Parker, 2012). CB contains several components of the RNA-induced silencing complex (Kotaja et al., 2006), mouse Vasa homolog (MVH/DDX4), a germ cell marker (Fujiwara et al., 1994; Noce et al., 2001), piRNA binding protein, and GRTH/DDX25 in high abundance (Sato et al., 2010; Meikar et al., 2014). piRNA and piRNA binding protein constitute a major part of the CB composition, and piRNAs derived from pachytene piRNA clusters and transposable elements. piRNAs are present both in the embryonic and the postnatal male germ cells, and their expression is induced abundantly in late meiotic cells and haploid round spermatids (Meikar et al., 2011, 2014).

GRTH is a member of the DEAD-box family of RNA helicases that play an essential role in the completion of spermatogenesis (Tsai-Morris et al., 2004; Dufau and Tsai-Morris, 2007). In germ cells, there are two species of GRTH, the 56-kDa non-phospho and the 61-kDa phospho forms. Previous studies revealed a missense mutation (R^242^H) of GRTH in Japanese azoospermic men, which resulted in the lack of phospho-GRTH at T239 (pGRTH) (Tsai-Morris et al., 2007, 2012; Raju et al., 2019). GRTH knock-in (KI) transgenic mice (human mutant GRTH gene with R^242^H) lack the 61-kDa phospho-species from the cytoplasm and CBs, while the non-phospho form from the cytoplasm, nucleus, and CBs are not affected. GRTH KI mice are sterile and have testis of reduced size which lack elongated spermatids and spermatozoa, with arrest at step 8 of round spermatids (RS) (Kavarthapu et al., 2019). Of note is the observed marked reduction in the size or complete loss of CBs in the round spermatids, indicating the importance of pGRTH in maintaining the structural integrity of CB (Kavarthapu et al., 2019). GRTH participates in the transport of specific mRNAs from the nucleus to the cytoplasmic sites, including CB, for storage prior to their translation during spermiogenesis (Sheng et al., 2006; Dufau and Tsai-Morris, 2007; Sato et al., 2010).

To understand the precise role of pGRTH in the regulation of germ cell-specific mRNA in CBs during spermiogenesis, CBs isolated from germ cells of WT and GRTH KI mice were analyzed using Illumina RNA-Seq to compare their transcriptome profiles. This study delineates the importance of pGRTH/DDX25 in CB regulation and participation in pathways which are essential for the progression and completion of spermiogenesis.

## Materials and Methods

### Animals

GRTH KI transgenic mice were generated with the human GRTH gene containing the R^242^H mutation found in Japanese infertile men as described previously (Kavarthapu et al., 2019). Briefly, WT and GRTH KI transgenic mice were genotyped after confirming the presence/absence of the transgene using two sets of primers Geno F1/Geno R1 and Geno F2/Geno R2 (Supplementary Table 1). All the animals were housed in pathogen-free and temperature- and light-controlled conditions (22°C), with 14:10-h light/dark cycle. All studies were approved by the National Institute of Child Health and Human Development Animal Care and Use Committee.

### Isolation of CBs From Mouse Testes

Isolation of CBs from adult mouse testes was carried out using the protocol (Meikar and Kotaja, 2014) with minor modifications. Testes from 10 WT and homozygous GRTH KI mice (45 days) (two mice for each sample, *N* = 5) were finely minced and digested in phosphate-buffered saline (PBS) containing 0.5 mg/ml of collagenase with 1 mg/ml glucose for 60 min at room temperature. Digested seminiferous tubules were filtered using a 100-μm cell strainer (BD Biosciences) and centrifuged for 5 min at 300 *g* at 4°C. After two washes in cold PBS, the cells were crosslinked with 0.1% paraformaldehyde (PFA; Thermo Scientific, MA, United States) for 20 min at 22°C. The reaction was stopped by adding 0.25 M glycine, pH 7.0 (Bostonbio, MA, United States). The cells were washed in PBS once, resuspended in RIPA buffer [(Millipore-Merck); 150 mM NaCl, 50 mM Tris–HCl, pH 7.5, 1 mM DTT, 1% Non-idet P40, 0.5% sodium deoxycholate with 1:200 RNase Inhibitor (Thermo Scientific), and protease inhibitor cocktail (Thermo Scientific)], and sonicated (five cycles of 20 s with 45-s intervals). The cell lysate was centrifuged for 10 min at 500 *g* at 4°C. The pellet containing the CBs was suspended in RIPA buffer and sonicated (two cycles of 20 s with 45-s intervals), filtered with a 5-μm filter (Millipore-Merck), and used for CB isolation. Aliquots of germ cells, pellet (PEL), and filtered pellet (FPEL) fractions were collected to monitor the success of the procedure. Fifty microliters of Protein G Dynabeads (Thermo Scientific) was coupled with 8 μg of commercial rabbit polyclonal anti-MVH antibody (cst#8761; Cell Signaling) or control rabbit IgG for 3 h according to the manufacturer’s instructions. After two washes with citrate phosphate buffer containing 0.1% Tween, the Dynabeads–IgG complex was stored in RIPA buffer briefly. The FPEL was pre-cleared with 10 μl of washed Protein G Dynabeads (Thermo Scientific) for 30 min at 4°C and used for immunoprecipitation (IP) overnight with rotation at 4°C with the Dynabeads–IgG complex. On the next day, the Dynabeads–IgG–CB complexes were washed three times in 1 ml of RIPA buffer. Crosslinking was reversed in the same buffer (for RNA analysis) or in 1 × Laemmli/SDS sample buffer (for protein analysis) at 70°C for 45 min. Subsequently, the Dynabeads–IgG complex containing CBs was used for RNA isolation.

### Immunofluorescence and Electron Microscopy Analysis

To monitor the success of the CB isolation procedure, the CB samples (5 μl) were fixed using a drying-down fixing solution (0.15% TritonX-100 and 1% PFA in PBS; 10 μl/slide) on a precleaned slide and air-dried overnight in a humidified chamber at 22°C. Once the slides were completely dried, these were post-fixed using 2% PFA for 5 min and then washed and treated with 0.2% Triton-X-100 in PBS for 2 min. Then, the slides were blocked using 5% BSA for 1 h in a humidified chamber and immunoprobed using anti-MVH/DDX4 antibody (1:1,000; cst#8761; Cell Signaling) overnight at 4°C. Following incubation with Alexa Fluor 568 secondary antibody (1:500) or Alexa Fluor 488 secondary antibody (1:500) for 1 h at 22°C, the slides were washed with phosphate-buffered saline–Tween^®^ 20 (PBST; twice, 5 min each) and PBS (twice, 5 min each) and mounted with ProLong Diamond antifade reagent (Thermo Scientific). These slides were imaged using a Zeiss LSM 710 confocal microscope (Carl Zeiss, CA, United States) and analyzed using ZenPro software (Carl Zeiss, CA, United States). Immunofluorescent staining of drying-down slide preparations is a reliable method to visualize the CBs. Testicular sections or germ cell preparations obtained from WT and KI mice were immunoprobed using anti-MVH/DDX4 antibody (1:1,000; cst#8761; Cell Signaling) overnight at 4°C. Following incubation with Alexa Fluor 488 secondary antibody (1:500) for 1 h at 22°C, the slides were washed with PBST (twice, 5 min each) and PBS (once, 5 min each) and mounted with ProLong Diamond antifade reagent with DAPI (Thermo Scientific). These slides were imaged using a Ziess Axioplan 2 fluorescence microscope (Carl Zeiss, CA, United States) and were analyzed using ZenPro blue and Axiovision v4.5 software (Carl Zeiss, CA, United States). Electron microscopy studies were carried out as described previously (Kavarthapu et al., 2019). Briefly, testicular tissue isolated from WT and GRTH KI mice were first fixed in 2.5% glutaraldehyde buffer at 4°C overnight, and then the tissues were post-fixed with 1% osmium tetroxide and stained using 2% uranyl acetate, dehydrated, and then embedded in Spurr’s epoxy. An EM ultramicrotome (Leica, Wetzlar, Germany) was used for making ultra-thin sections and were post-stained with lead citrate and observed under a JEOL JEM-1400 transmission electron microscope (JEOL USA, Inc., Peabody, MA, United States).

### Western Blot Analysis

CBs isolated from the testes of WT and GRTH KI mice were subjected to western blot analysis. The protein G–IgG–CB complex was washed in RIPA buffer and reverse cross-linked at 70°C for 45 min in the 1× Laemmli/SDS sample buffer containing reducing agents. The resulting protein samples were centrifuged at 1,500 *g* for 5 min at 22°C. The supernatants collected were run on 4–12% Bis-Tris gel (Thermo Scientific) and transferred onto a nitrocellulose membrane in an iBlot (Thermo Scientific). The membrane was blocked with 5% skimmed milk powder in tris-buffered saline and then incubated with either of the following primary antibodies: affinity-purified anti-GRTH rabbit polyclonal antibody (1:500 dilution), anti-MVH/DDX4 polyclonal (1:500; cst#8761; Cell Signaling), mouse Argonaute/PIWI family RNA binding protein (MIWI) monoclonal antibody (CB control; 1:500; cst#6915; Cell Signaling), brain and muscle ARNT-like 1 (BMAL1) rabbit monoclonal antibody (1:500 dilution; cst#14020; Cell Signaling), circadian locomotor output cycles protein kaput (CLOCK) rabbit monoclonal antibody (1:500 dilution; cst#5157; Cell Signaling), Ybx3 mouse monoclonal antibody (1:500 dilution; LS-C105064; LS Bio), PRM1 rabbit polyclonal antibody (1:500 dilution; HPA055150; Millipore Sigma, St. Louis, MO, United States), and PRM2 rabbit monoclonal antibody (1:500 dilution; Sc-30172; Santa Cruz Biotechnology, Dallas, Texas). After the antibody incubation and washing steps, the membranes were incubated with the respective secondary antibodies conjugated with Poly-HRP (1:2,000; Thermo Scientific). Immunosignals were detected by a super-signal chemiluminescence system (Thermo Scientific).

### Total RNA Preparation, Library Construction, RNA-Seq (Illumina HiSeq2500)

Total RNA was extracted from reverse cross-linked Dynabeads–IgG–CB complexes using phenol/chloroform/isoamyl alcohol (25:24:1, v/v; Thermo Scientific). RNA quality and quantity were assessed using the RNA Nano 6000 Assay Kit in an Agilent Bioanalyzer 2100 system (Agilent Technologies, Santa Clara, CA, United States). Sequencing libraries were prepared using a TruSeq Stranded mRNA Prep Kit (Illumina), without the polyA selection step. Sequencing was performed *via* a paired-end 75 cycle on Illumina HiSeq 2500 (Molecular Genomics Core, NICHD). The RNA-Seq data have been submitted to the NCBI (https://www.ncbi.nlm.nih.gov/geo), with GEO accession number GSE148897.

### RNA-Seq Data and Differences in the Abundance of Transcripts

RNA-Seq reads were trimmed and aligned using STAR to mouse mm10 reference genome sequences using Partek Flow for next-generation sequencing data (http://www.partek.com/partek-flow/). The transcript abundance was quantitated using Partek E/M (mm10-Ensembl Transcripts release 97) annotation model to obtain gene counts. Principal component analysis was done on gene counts to determine the variability in the data set (data not shown). Differences in mRNA/transcript abundance between WT and GRTH KI were generated using DESeq2 (fold change < -1.5 or > 1.5; false discovery rate/*P*_adj_ < 0.05; *P* < 0.05) in Partek Flow.

### Gene Ontology Analysis

Gene Ontology (GO) enrichment analysis was performed to identify the functional classes using metascape software. Transcripts were annotated to three main GO categories—biological process (BP), cellular component (CC), and molecular function (MF)—and were represented separately. Kyoto Encyclopedia of Genes and Genomes (KEGG) pathway enrichment analysis was performed on all the transcripts which show differential abundance using metascape to identify the important pathways (*P* < 0.05).

### Protein–Protein Interaction Network Analysis Using String/Cytoscape

To better illustrate the interactions among the differentially enriched transcripts, The Search Tool for the Retrieval of Interacting Genes/Proteins (STRING) was used to construct and visualize the PPI network using Cytoscape software (version 3.7.2), and to analyze by network analyzer plugin, cytoHubba app was used to find the top 100 hub genes based on the maximal clique centrality (MCC) algorithm, and the PPI network was constructed. Four different clusters were identified using the MCODE app in Cytoscape and were further assessed based on functional enrichment analysis.

### Validation of RNA-Seq Using Real-Time Quantitative PCR Analysis

qRT-PCR was used to validate the differentially enriched transcripts obtained from RNA-Seq transcriptome analysis. Total RNA prepared for RNA sequencing library preparation was used to perform qRT-PCR. One microgram of total RNA was used to prepare cDNA using the Iscript first-strand synthesis kit (Bio-Rad Laboratories, Hercules, CA, United States), and qRT-PCR was performed with Fast SYBR green using a set of specific gene primers (Supplementary Table 1) in a 7500 Fast Real-Time PCR machine (Applied Biosystems, Foster City, CA, United States). All reactions were performed in triplicates, and the cycle threshold (Ct) values were normalized to *DDX-4/Vasa/MVH* as the reference gene, and the comparative quantification of mRNA was performed using the 2^–ΔΔCt^ method.

### Immunoprecipitation of GRTH Protein–RNA Complex and qRT-PCR Analysis

The CB-enriched fraction (FPEL; 0.5 mg) isolated from the testes of WT and GRTH KI mice was reverse cross-linked and pre-cleared using 50 μl of protein A-agarose beads (Thermo Scientific) and 1 μg of rabbit IgG containing IP binding buffer (Thermo Scientific, United States) for 30 min at 4°C in a rocker. Upon spinning, the resulting pre-cleared supernatant obtained was incubated with 5 μg of anti-GRTH rabbit polyclonal antibody (Kavarthapu et al., 2019) or rabbit IgG at 4°C overnight to co-immunoprecipitate the GRTH–RNP complex. Upon overnight incubation, the GRTH–RNP complex was incubated with 50 μl of protein A-agarose beads and incubated for 2 h at 4°C. The resulting protein–RNP complex was washed with IP binding buffer (four washes), and the total RNA was isolated using the phenol/chloroform/isoamyl alcohol (25:24:1, v/v; Thermo Scientific, Waltham, MA, United States) method. The first-strand cDNA was prepared using iscript first-strand synthesis kit (Bio-Rad Laboratories, Hercules, CA, United States), and qRT-PCR was performed with Fast SYBR green using a set of gene-specific primers (Supplementary Table 1) in a 7500 Fast Real-Time PCR machine (Applied Biosystems, Foster City, CA, United States).

### *In situ* Hybridization

A germ cell suspension was obtained by squeezing the seminiferous tubules of WT and GRTH KI mice with fine forceps in 1% PFA containing 100 mM sucrose (RNase free) in a petri dish. The germ cell suspensions were spread onto slides pre-coated/cleaned with 1% PFA with 0.15% Triton X-100 and dried overnight in a humidified chamber. *In situ* hybridization was carried out using a slightly modified procedure (Rajakumar and Senthilkumaran, 2014). In short, the fixed cells were permeabilized with Proteinase K (in PBST) for 10 min and washed twice with PBST (5 min each). Pre-hybridization was carried out using a hybridization buffer: formamide (1:1 v/v) at 55°C for 1 h in a hybridization oven. The respective probes (100 ng) in hybridization buffer/formamide (1:1 v/v) were pre-heated at 85°C for 5 min, cooled on ice for a few minutes, and added onto the slides and incubated for 4 h in a hybridization oven. For post-hybridization washes, wash buffer containing a decreasing concentration of SSC at 60°C was used. The slides were then blocked with a blocking buffer (Thermo Scientific) containing 5% normal goat serum (Sigma) in 1× maleic acid buffer (Thermo Scientific) for 1 h at room temperature in a humidified chamber. The slides were incubated with anti-DIG Rhodamine secondary antibody (1:500) and specific anti-MVH/DDX4 rabbit polyclonal antibody (Cell Signaling, cst#8761; 1:1,000 dilution) in blocking buffer (Thermo Scientific) containing 5% normal goat serum (Sigma) in maleic acid buffer at 4°C overnight. The slides were subsequently washed with DIG wash buffer (twice for 10 min each), PBST (5 min), and PBS (5 min) and incubated with Alexa Fluor 488 for 1 h at room temperature. The slides were washed with PBST (twice, 5 min each), PBS (twice, 5 min each), and mounted with ProLong Diamond antifade reagent with DAPI (Thermo Scientific). These slides were imaged using a Ziess Axioplan 2 fluorescence microscope (Carl Zeiss, CA) and were analyzed using ZenPro blue and Axiovision v4.5 software (Carl Zeiss, CA, United States).

### Statistical Analyses

The significance of the differences between groups was determined by Tukey’s multiple-comparison test (one-way ANOVA analysis) using the Prism software program (GraphPad Software, Inc., San Diego, CA, United States) and Microsoft Excel (Microsoft).

## Results

### Analysis of CBs From WT and GRTH KI Mouse Testes

**FIGURE 1:**
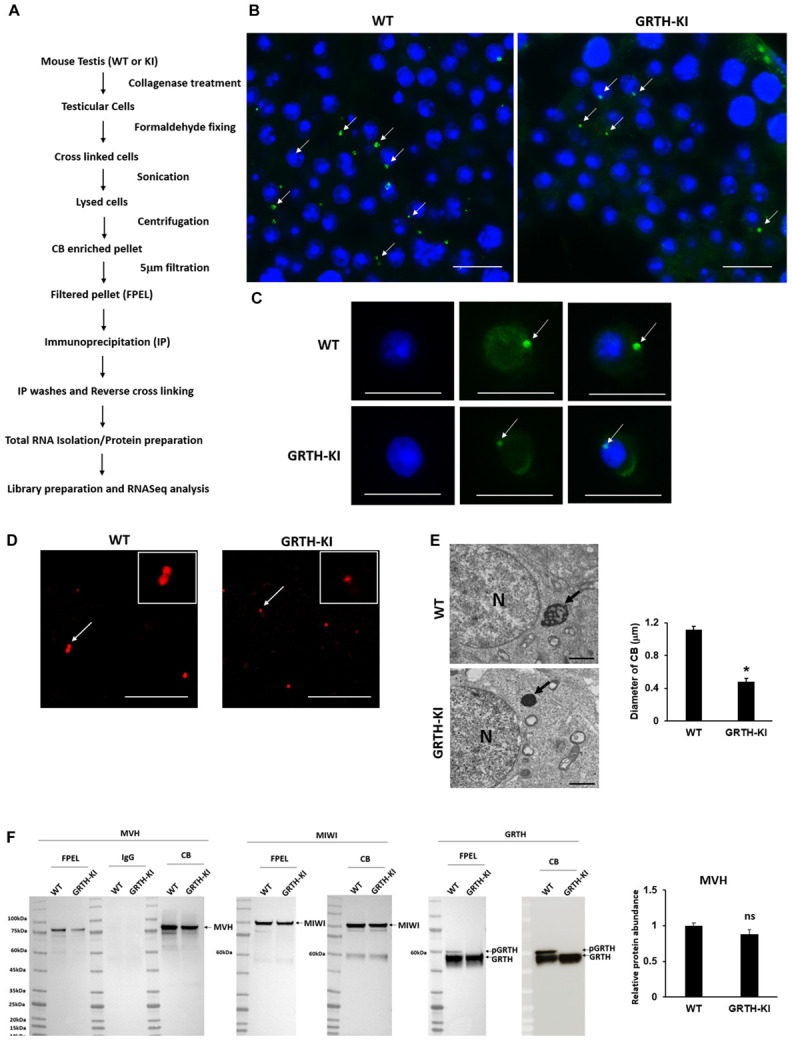
Isolation and characterization of chromatoid bodies (CBs) from wild-type (WT) and knock-in (KI) mice. **(A)** Overview of CB isolation protocol from germ cells of mouse testes. **(B)** Immunofluorescence staining of MVH/DDX4 (green) in the testicular tissue sections of WT and KI mice; CBs are indicated by an arrow. Scale bar, ∼25 μm. **(C)** Immunofluorescence staining of MVH/DDX4 (green) in the round spermatids obtained from WT and KI mice; CBs are indicated by an arrow. Scale bar, ∼10 μm. **(D)** Immunofluorescence staining of MVH/DDX4 as a marker showing that CBs (red) isolated from KI mice are smaller in size compared to WT. Inset shows the detail of CBs indicated by an arrow. Scale bar, ∼25 μm. **(E)** Electron microscopy images from round spermatids showing a lobular structure with an irregular network of the less-dense strands characteristic of the CB clearly visible in WT, while in KI, CBs are markedly reduced in size. Arrows indicate CBs and N indicates the nucleus. *P*-values were calculated by two-tailed Students t-test (*P < 0.05). Scale bar, ∼1 μm. **(F)** Western blot analysis showing the complete loss of pGRTH proteins in CBs of KI mice compared to CBs of WT mice, while the levels of MVH, MIWI (CB control), and non-phospho GRTH were unaltered. FPEL, filtered pellet.

The size of the testis of KI mice was significantly reduced (*P* < 0.05) when compared to that of WT mice (Supplementary Figure 2). The CBs were isolated successfully from WT and KI mice (Figure 1A). The KI mice have smaller CBs in the testicular sections (Figure 1B) and round spermatids (Figure 1C). The success of the CB isolation procedure was confirmed using microscopy (Supplementary Figure 3). The CBs obtained from the WT group were markedly bigger in size compared to those of GRTH KI mice (Figure 1D). Furthermore, EM studies showed smaller and condensed CBs in KI mice, which is evident by a significant reduction in their size/diameter when compared to CBs of WT mice which display an amorphous “nuage” texture (Figure 1E). Among proteins extracted from CBs of WT and KI mice, the levels of MVH/DDX4, MIWI (CB control), and non-phospho GRTH were unaltered, while pGRTH protein was completely absent in the CBs of KI mice compared to WT (Figure 1F; Table 1A; Kavarthapu et al., 2019). In addition, there was no change in the expression of BMAL1, CLOCK, and YBX3 proteins in the CB of WT and KI mice. PRM1/PRM2 expression was not detected in the CB of WT and KI mice (Supplementary Figure 4).

**TABLE 1:** **(A)** List of important proteins with their cellular localization with respect to wild-type and knock-in mice.

List of proteins (WT)	Cytoplasm (WT)	Nucleus (WT)	CB (WT)
GRTH	Yes	Yes	Yes
pGRTH	Yes	No	Yes
MVH	Yes	No	Yes

**List of proteins (KI)**	**Cytoplasm (KI)**	**Nucleus (KI)**	**CB (KI)**

GRTH	Yes	Yes	Yes
pGRTH	No	No	No
MVH	Yes	No	Yes

### Overview of RNA-Seq Data Analysis

**FIGURE 2:**
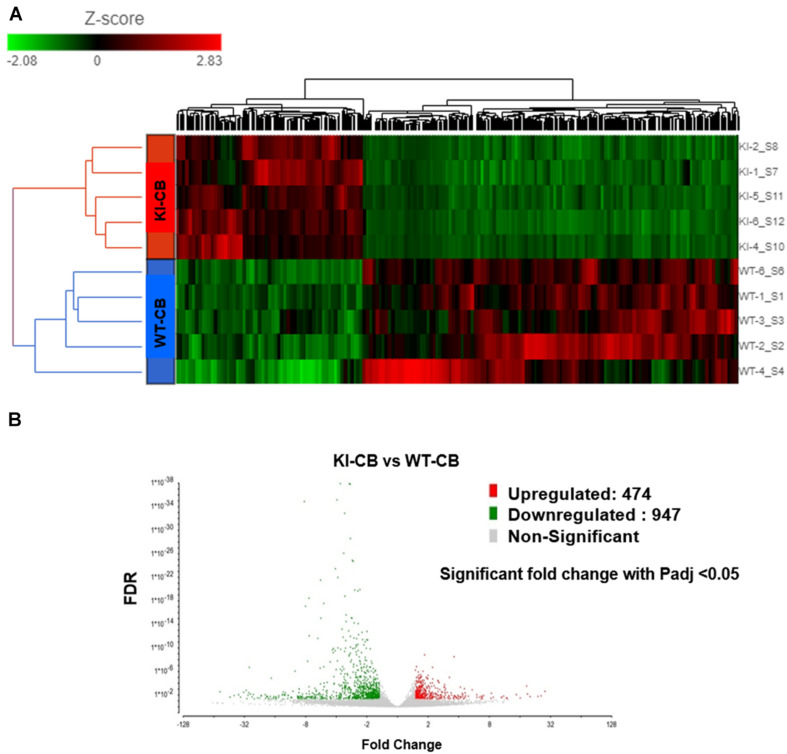
RNA-Seq analysis of RNA extracted from chromatoid bodies of wild-type (WT) and knock-in (KI) mice. **(A)** RNA-Seq data show a clustered heat map with differential abundance of transcripts (WT and KI; fold change < -1.5 or > 1.5; *P*_adj_ < 0.05). The red blocks represent the genes with an increase in abundance, and the green blocks represent a decrease in abundance of genes. The colored bar represents the transcript abundance levels. **(B)** Volcano plot (fold change vs. false discovery rate value) showing 1,421 differentially enriched transcripts from KI and WT groups (*n* = 5 per group). Significantly increased and decreased abundances of transcripts are highlighted in red and green, respectively. The X-axis shows the fold change in transcripts between different samples, and the Y-axis shows the statistical significance of the differences.

RNA-Seq was performed on RNA samples obtained from the CBs of WT and KI mice testes (*N* = 5) to analyze the transcriptomic profiles of CBs in the presence (WT mice) and in the absence of pGRTH (KI mice). Sequencing of CBs from WT and GRTH KI mice yielded 75.6 and 125.9 million reads, respectively. The average Phred score was 38 (with base calling error rate of almost 1 in 10,000), and more than 75% of reads could be mapped to the mouse reference genome. Separation of the GRTH KI and WT genotypes was evident in the hierarchically clustered heat map shown in the transcript abundance profiles (Figure 2A).

### Differences in Transcript Abundance Analysis Using GO and KEGG Pathways

**FIGURE 3:**
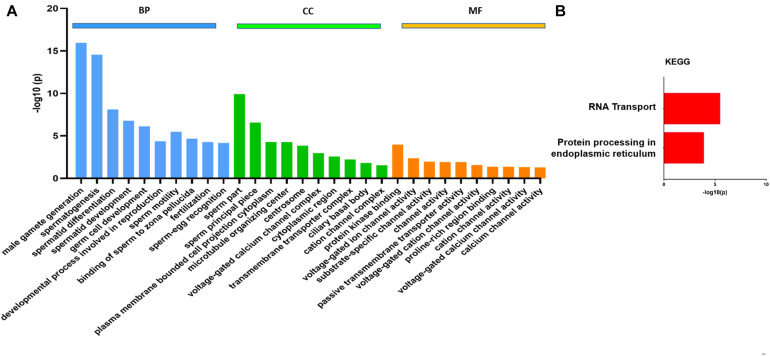
Gene Ontology (GO) analysis in differentially enriched transcripts obtained from RNA-Seq using STRING. **(A)** GO analysis (top 10) of transcripts (*P*_adj_ < 0.05) obtained from chromatoid bodies of wild-type and knock-in mice. The GO results were grouped into different functional categories: cellular component (CC), molecular function (MF), and biological process (BP). The Y-axis represents the number of genes in each GO term. **(B)** Kyoto Encyclopedia of Genes and Genomes enrichment analysis of the top two enriched pathways with -log10 *P*-value on the X-axis.

The transcript levels of several genes involved in spermatogenesis (Tnp1/2, Prm1/2, TSSK 2/3/6, *etc*.) were also significantly reduced in the CB of KI mice similar to that which we found in RNA-seq data from germ cells of KI mice (Kavarthapu et al., 2020; Supplementary Table 5). Differences in the transcript abundance between CBs isolated from WT and KI mice were analyzed using DESeq2. In total, 1,421 genes show differential abundance, including 474 genes that showed an increase in abundance and 947 genes with a decrease in abundance (*P*_adj_ < 0.05; Supplementary Table 6). Volcano plots (Figure 2B) were used to infer the overall distribution of differentially enriched transcripts. The GO functional enrichment analysis was classified into three categories: BP, CC, and MF (Supplementary Table 7). Only significant GO categories with *P* < 0.05 were chosen for analysis, and differentially abundant transcripts (top 10) of gene-enriched GO terms were identified. In the BP group, transcripts which show differential abundance were mainly enriched in spermatogenesis, spermatid differentiation, and spermatid development, motility, and fertilization (Figure 3A). In the CC group, transcripts which show differential abundance were enriched in sperm principle piece, microtubule organizing center, cation channel complex, *etc*., while in the MF group, transcripts which show differential abundance were enriched in protein kinase binding, voltage-gated cation channel activity, proline-rich region binding, passive transmembrane transporter activity, *etc*. (Figure 3A). KEGG pathway enrichment analysis revealed that all the differentially enriched transcripts (*P* < 0.05) were associated with either RNA transport or protein processing in the endoplasmic reticulum pathways (Figure 3B; Table 1B). We also compared the transcript abundance (fold change) of genes from CB RNA-seq (this study) and germ cells RNA-seq data (expression in fold change) that were previously reported from our laboratory (Kavarthapu et al., 2020). Among 947 genes with a decrease in abundance in CBs of KI mice, we found 272 genes that were downregulated and eight genes that were upregulated in the germ cells of KI mice. Among 474 genes with an increase in abundance in CBs of KI mice, we found 18 genes that were upregulated in the germ cells of KI mice, which were illustrated in the form of Venn diagrams (Supplementary Figure 8). The detailed list of genes with fold change between these datasets are given in Supplementary Table 5.

**Table:** **(B)** Kyoto Encyclopedia of Genes and Genomes pathway enrichment analysis of differentially enriched transcripts showing two essential pathways.

List of KEGG pathways	List of genes
RNA Transport	Eef1a1, Eif4e, Eif4ebp2, Pabpc1, Rasl2-9, Ube2i, Eif4e2, Nup160, Pabpc6, Nup35, 1700009N14Rik, 4930444G20Rik, Paip1, Thoc5, AF366264, Nup133, Pabpc4l, Upf2, Gm5415, Gm9839, Ncbp1
Protein processing in ER	Atf4, Bag1, Hspa1l, Hspa2, Hsp90ab1, Ube2d2a, Dnajc5b, Edem3, Ero1lb, Stt3b, Tram1, Dnajb1, Rpn1, Selenos, Yod1, Sel1l2, Ubqlnl, Mbtps2

### PPI Network Analysis Showing Important Network/Pathway Interactions

**FIGURE 4:**
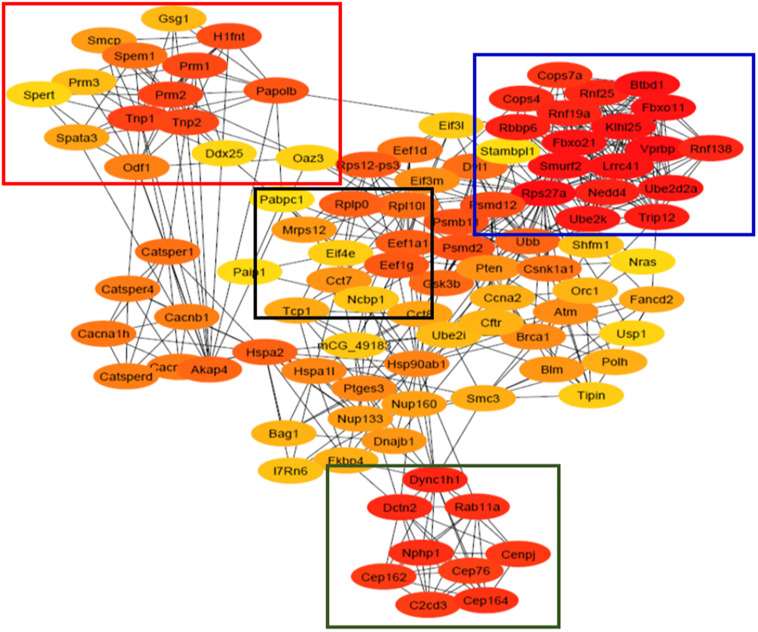
Protein–protein interaction (PPI) network of the top 100 hub genes identified using cytoHubba plugin in Cytoscape software. Four different clusters were obtained from this network using MCODE which belong to genes involved in spermatogenesis (red box), ubiquitination pathway (blue box), RNA transport (black box), and centrosome organization and organelle assembly (green box). The colored nodes represent genes/proteins having high (red) and low (yellow) PPI scores. The edges represent the protein–protein interaction/associations.

All 1,421 differentially enriched transcripts (*P*_adj_ < 0.05 and < −1.5/ > 1.5 fold) were analyzed using the STRING tool in Cytoscape software to visualize the PPI network. The top 100 hub genes in the PPI network were chosen based on the MCC algorithm using cytoHubba app in Cytoscape (Figure 4). A total of 99 nodes and 541 edges were mapped in the PPI network, and all these four important modules (squared) which were interconnected to each other belong to major pathways/processes, including RNA surveillance/transport, spermatogenesis, chromatin condensation/compaction, ubiquitin-proteasome pathway (UPP), centrosome organization, and organelle assembly (Figure 4).

### RNA-Seq/Differences in Transcript Abundance Validation Using qRT-PCR

**FIGURE 5:**
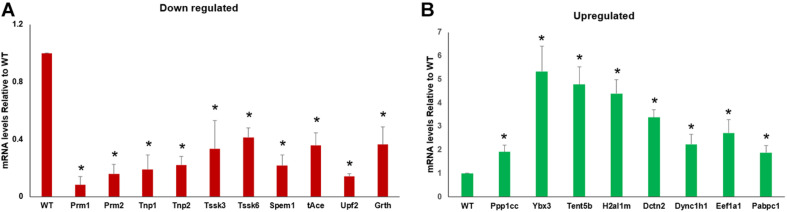
qRT-PCR validation of RNA-Seq data with RNA obtained from chromatoid bodies (CBs) isolated from wild-type (WT) and knock-in (KI) mice. The transcript levels showing decreased **(A)** and increased **(B)** abundances in CBs obtained from KI *vs*. WT mice were normalized using actin. All statistical analyses were performed using Student’s *t*-test (*P < 0.05), and data represent mean ± SEM of three independent experiments in triplicates.

To validate the genes that are enriched in RNA-Seq data, specific sets were selected based on GO and KEGG results, and their transcript abundance was analyzed using qRT-PCR. Overall, the qRT-PCR analysis results showed a good correlation with the RNA-Seq/transcriptome analysis results. The transcript levels of *Tnp1/2*, *Prm1/2*, *Spem1, Tssk 3/6*, *Grth*, *tAce*, and *Upf2* involved in spermatid development, differentiation, chromatin compaction, and remodeling were significantly (*P* < 0.05) reduced in the CB of GRTH KI mice compared to that of WT mice (Figure 5A). Furthermore, transcripts encoding for factors involved in RNA transport, storage, transcriptional, and translational regulation (*Eef1a1, Ppp1cc*, *Pabpc1*, *Ybx3*, *Tent5b*, *H2al1m*, *Dctn2*, and *Dync1h1*) were found to be increased (Figure 5B) in the CB of GRTH KI mice compared to CB of WT mice, confirming the consistency of the RNA-Seq data.

### GRTH-IP and RNA Analysis Reveal the Importance of pGRTH Binding to Specific Transcripts in the CBs

**FIGURE 6:**
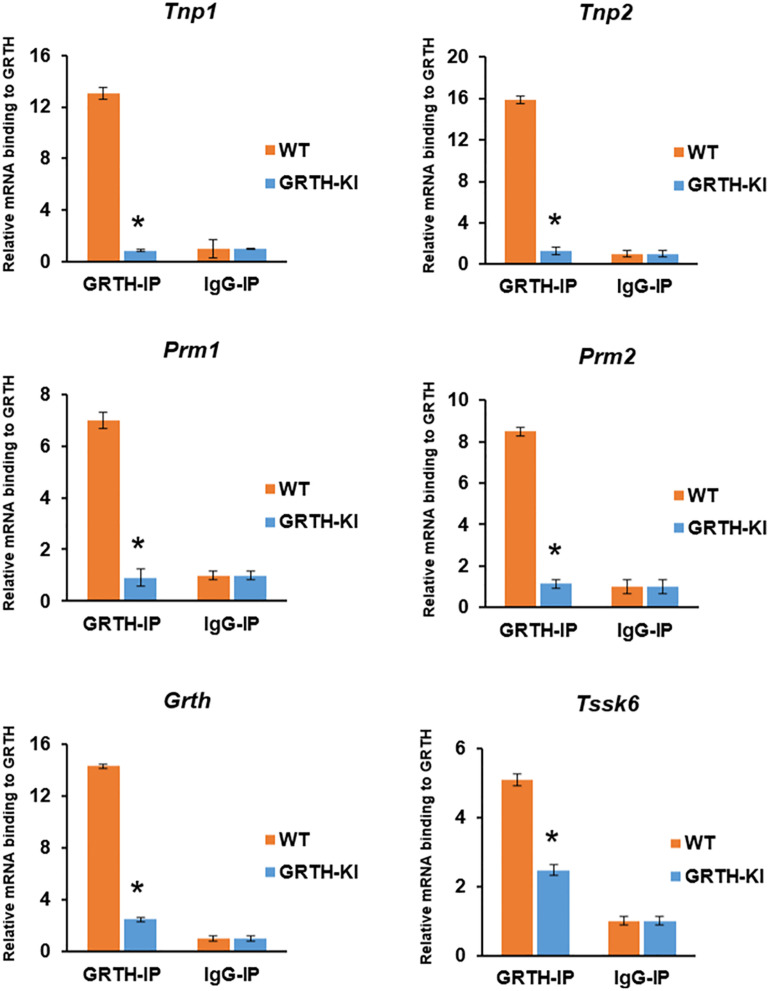
Binding of GRTH to specific germ cell mRNAs in chromatoid bodies (CBs) isolated from wild-type (WT) and knock-in (KI) mice. Relative binding of *Tnp1/2*, *Prm1/2*, *Grth*, and *Tssk6* mRNA to GRTH protein in CB of germ cells in the testes from WT and GRTH-KI mice. All statistical analyses were performed using Student’s *t*-test (*P < 0.05), and data represent mean ± SEM of three independent experiments in triplicates.

Successful GRTH-IP were confirmed using western blot (Supplementary Figure 9) prior to isolating GRTH-bound mRNAs. GRTH-IP mRNA binding experiments reveal that GRTH protein binding to specific germ cell mRNAs (*Tnp1/2*, *Prm1/2, Grth*, and *Tssk6)* in CBs was decreased significantly to basal IgG levels (*P* < 0.05) in GRTH KI mice (Figure 6). The mRNA binding function in CBs was impacted significantly in GRTH KI mice (lacks pGRTH), which resulted in impaired chromatin compaction and spermatid elongation and stalled spermiogenesis at step 8.

### Role of pGRTH in the Storage of *Tnp2*, *Prm2*, and *Grth* mRNAs in the CB of Round Spermatids

**FIGURE 7:**
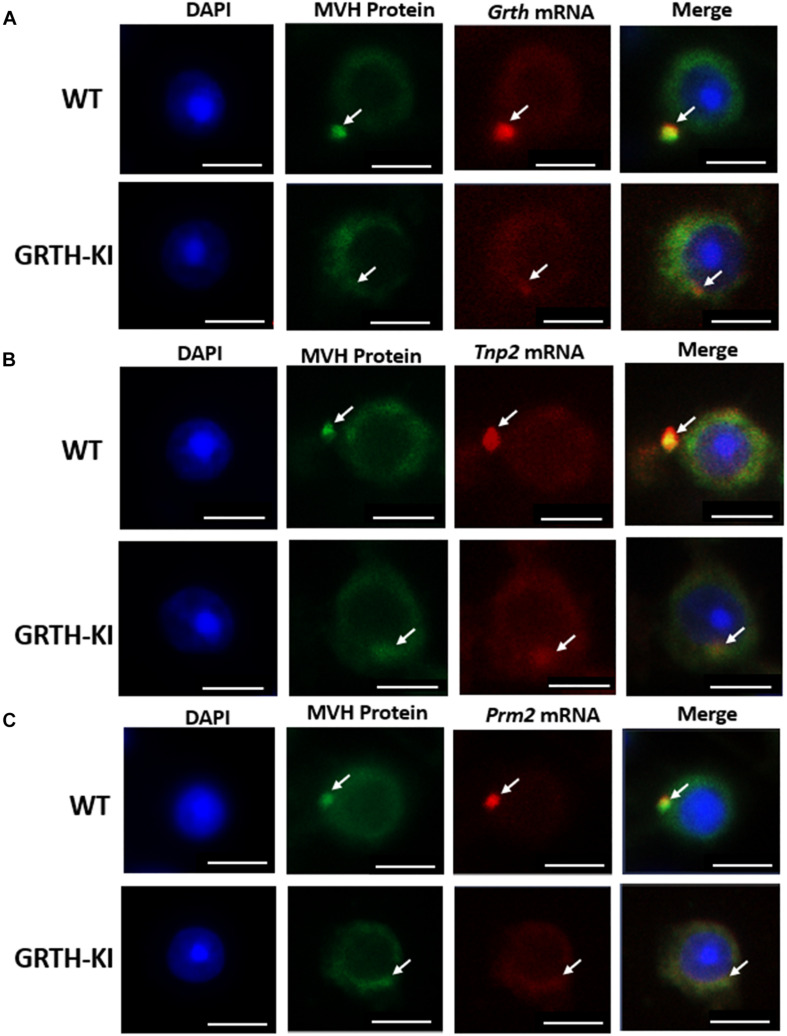
Chromatoid bodies (CBs) accumulate mRNAs involved in spermiogenesis. Fluorescence microscopy showing the *in situ* localization of selected mRNAs (red) with MVH protein (green) in CBs of round spermatids from WT and KI mice. **(A)** *ISH* of *Grth* mRNAs. **(B)** *ISH* of *Tnp2* mRNAs. **(C)** *ISH* of *Prm2* mRNAs. CBs of KI mice are indistinct/smaller in size compared to CBs of WT mice. Co-staining with MVH confirmed their localization in the CB. The nuclei are stained with DAPI (blue). CB is marked by white arrows. Scale bar, ∼5 μm.

The ISH analysis revealed that the presence of *Tnp2*, *Prm2*, and *Grth* mRNAs was more abundant in CBs than in the cytoplasm of round spermatids (Figures 7A–C). In WT mouse germ cells, *Tnp2*, *Prm2*, and *Grth* mRNAs (red) and MVH-protein (green) were distinctively noticeable (Figures 7A–C), while in GRTH KI mouse germ cells, *Tnp2*, *Prm2*, and *Grth* mRNAs were drastically reduced in CBs, and the sizes of the CBs were smaller, with less distinction through the cytoplasm (Figures 7A–C). In GRTH KI mice, the mRNA storage decreases due to lack of pGRTH, which is one of the CB structural proteins. Taken together, these results indicate that loss of pGRTH impaired the storage of these specific mRNAs in the CBs of round spermatids which are later required for their translation and are essential for the progress of spermiogenesis. These findings reveal the importance of CBs in mRNA storage and the role of pGRTH in maintaining their structure during specific stages of spermiogenesis.

## Discussion

The GRTH KI mice with loss of pGRTH protein, lacking elongating spermatids due to arrest at step 8 of spermiogenesis, contain round spermatids with structural changes in the CBs (Kavarthapu et al., 2019). However, studies of specific changes in the CB at the level of the transcriptome were of immediate relevance to link the function of pGRTH and its effects in the subsequent translational events with this site. In this study, we demonstrated the importance of CBs in spermiogenesis in the presence and in the absence of pGRTH by analyzing the abundance of transcripts of intrinsic importance in the progression of spermatogenesis through an evaluation of transcriptome profiles of CBs from WT and GRTH KI mice together with their association with GRTH protein and localization of selected mRNAs which are involved in the initial and late stages of spermiogenesis.

GRTH is an RNA binding protein involved in the transport of germ cell-specific mRNAs from the nucleus to the cytoplasmic site and is essential for the completion of spermatogenesis (Sheng et al., 2006; Dufau and Tsai-Morris, 2007). GRTH associates with actively translating polyribosomes and regulates the translation of specific germ cell genes likes *Tnp1/2* and *Prm1/2*, which are involved in sperm maturation (Tsai-Morris et al., 2012). The pGRTH is shown to regulate the translation of the *Tnp2* gene which occurs in a 3′-UTR-dependent manner (Kavarthapu et al., 2019). During the process of spermiogenesis, RS undergo an elongation process where the histones are first replaced by highly basic proteins TNP1/2 and subsequently by PRM1/2. These chromatin remodeling proteins play a crucial role in hyper-chromatin condensation and reshaping the nucleus of elongating and condensing spermatids (Boa and Bedfors, 2016).

In this study, in a GO enrichment analysis, transcripts which show differential abundance to be prevalent in spermatogenesis, spermatid differentiation, and development are significantly decreased. The germ cell-specific transcripts like *Tnp2*, *Prm2*, and *Grth* in CBs decreased significantly, and this was confirmed by the *in situ* localization of these transcripts in CBs of RS of both WT and GRTH KI mice (Figure 7). Furthermore, the association of pGRTH protein with specific germ cell mRNAs (*Tnp1/2*, *Prm1/2*, *Grth*, and *Tssk6)* in CBs was also found to be decreased due to the loss of pGRTH in GRTH KI mice. These results demonstrate the importance of pGRTH as an RNA binding protein for the above-mentioned genes and as a stabilizer of their mRNAs in CBs until translation at the later stages of spermiogenesis. The absence of the phospho form of GRTH in the CBs of KI mice also has a direct impact on the structure of CBs in RS as GRTH is one of the important structural proteins along with other CB markers MVH and MIWI (Sato et al., 2010; Kavarthapu et al., 2019). The expression of MVH and MIWI protein was unaltered in the CBs of KI mice compared to WT. MVH is an essential factor in the piRNA processing pathway (Kuramochi-Miyagawa et al., 2010; Meikar et al., 2011). Also unchanged in the KI mice are CB transient proteins, CLOCK and BMAL1, with their expression overlapping with CB marker proteins in an RS stage-specific manner (Peruquetti et al., 2012). Targeted ablation of these circadian proteins BMAL1 and CLOCK results in infertility in mice with significant characteristic morphological alterations of CBs (Peruquetti et al., 2012) which are clearly different from those observed in mice lacking pGRTH (KI) (Kavarthapu et al., 2019) and GRTH null mice (Tsai-Morris et al., 2004). From the unaltered levels of CLOCK and BMAL1 proteins in the CB of KI in our present studies, we can exclude pGRTH as a regulator of these proteins.

The RNA profiles of CBs obtained from WT and GRTH KI were distinct, which is apparent from the hierarchically clustered heat map and volcano plots of differentially enriched transcripts, which depict genes with decrease and increase in abundance in CBs of GRTH KI mice. The KEGG pathway enrichment analysis revealed that most of the differentially enriched transcripts were mainly associated with both RNA transport and protein processing in endoplasmic reticulum pathways, which also confirms the impact on RNA transport into the CBs due to the loss of pGRTH in the GRTH KI mice. To compensate for the loss, the genes essential for RNA stability, including *Pabpc*, were increased inside the CBs of GRTH KI mice.

Furthermore, the inter-relation among differentially enriched transcripts and PPI network was constructed. This showed four classes/modules with key hub genes belonging to spermatogenesis, UPP, mRNA metabolism/regulation, and microtubule organization/motility. The UPP genes are implicated in histone ubiquitination and acetylation, which play a crucial role in chromatin remodeling essential for spermatid elongation during spermiogenesis (Lu et al., 2010; Sheng et al., 2014). Recently, our studies in KI mice have shown that loss of pGRTH results in a reduced expression of UPP genes (*Ube2J1*, *Ube2K*, *Ube2W*, *Rnf8*, *Rnf133*, and *Rnf138*) and *Mof/Myst1*, which subsequently impaired the ubiquitination of H2A/B and the acetylation of H4 essential for histone eviction and replacement with transition proteins, followed by protamines during spermiogenesis (Kavarthapu et al., 2020). During elongation of RS, histones were first replaced by transition proteins (TNP1/2), which constitute 90% of the chromatin basic proteins, and then followed by the deposition of the protamines (PRM1/2), which results in hyper-chromatin condensation and compaction of the RS nucleus (Boa and Bedfors, 2016; Schneider et al., 2016; Kavarthapu et al., 2019). The transcripts of *Tnp1/2*, *Prm1/2/3*, *Tssk2/3/6*, and *Spem1/2* were significantly reduced in the CB of GRTH KI mice. The reduction in these transcripts in the CBs is probably due to the decreased mRNA transport and/or mRNA degradation as they are not bound to pGRTH in GRTH KI mice. This eventually led to failure of chromatin remodeling, which is essential for the condensation of chromatin in developing spermatids during spermiogenesis. *Tssk2/6* KO mice are sterile, with defects in spermatogenesis due to post-meiotic chromatin remodeling and intracellular signal transduction defects (Shima et al., 2004; Spiridonov et al., 2005). *Spem1* is exclusively expressed in the round and elongating spermatids, and the loss of this protein causes aberrant cytoplasm removal, sperm deformation, and male infertility (Zheng et al., 2007). Interestingly, mice deficient of *Tnp1*/*2* (Meistrich et al., 2003; Shirley et al., 2004) and Camk4 (Wu et al., 2000) also show failure of cytoplasmic removal during spermiogenesis. Furthermore, the exchange of basic nuclear proteins is impaired in male germ cells lacking Camk4 (Wu et al., 2000). This explains the impact of the loss of pGRTH on these germ cell-specific transcripts (*Tnp1/2*, *Prm1/2/3*, *Tssk2/3/6*, and *Spem1/2*) and their cumulative effects which result in complete spermatogenic arrest just before spermatid elongation at step 8 in GRTH KI mice. *Upf2*, which is involved in nonsense-mediated mRNA decay, an mRNA surveillance pathway that eliminate transcripts with premature stop codons, was decreased, resulting in inefficient mRNA surveillance in GRTH KI mice. To support the role of CBs in mRNA processing, splicing, regulation, translation, and mRNA turnover, a very high number of poly(A)-binding proteins (Pabp) and Poly (rC) binding protein (Pcbp) are found in CBs (Meikar et al., 2014; Peruquetti, 2015). In the current study, transcripts of *Pabpc 1/6/4l* and *Pcbp 2/3* were reduced significantly in CBs of GRTH KI mice, as the decreased mRNAs in the CB of KI mice require more stabilization from Pabpc and Pcbp proteins. Furthermore, *Pabpc1/4*, *Pcbp1/3* were abundantly expressed in round spermatids prior to their elongation, and hence their transcript levels were abundant in GRTH KI mice. The m7G cap at the 5’ end associates with eukaryotic initiation factor 4E (eIF4E) which, through interaction with other factors, regulates gene expression. Transcripts of several initiation factors (*eIF4e2*, 4ebp2, 3l and 3m) together with mRNAs related to the 60S subunit (Rpl10l/Rplp0) were increased and accumulated in CB as mRNAs which could not get transported from the CB to the polyribosomes for translation; these instead remain stored in the CB in GRTH KI mice due to the loss of pGRTH, hence the disruption of RNA quality control and translation machinery in germ cells of GRTH KI mice.

## Conclusion

In summary, our data demonstrate that pGRTH is required for the maintenance of the CB structure and is crucial for the storage of germ cell-specific mRNAs until their translation in later stages of spermatids during spermiogenesis.

## Data Availability Statement

The RNA-Seq data have been submitted to the NCBI (https://www.ncbi.nlm.nih.gov/geo) with GEO accession number GSE148897.

## Ethics Statement

The animal study was reviewed and approved by National Institute of Child Health and Human Development Animal Care and Use Committee.

## Author Contributions

RA, RK, and MD conceived and planned the experiments. RA performed the experiments and analyzed the data. RA and MD discussed the results and wrote the manuscript. SC contributed to part of the RNA-Seq data analysis. All authors contributed to the article and approved the submitted version.

## Conflict of Interest

The authors declare that the research was conducted in the absence of any commercial or financial relationships that could be construed as a potential conflict of interest.

## Supplementary Material

Supplementary Table 1 List of primers used for genotyping and validation of differentially enriched transcripts using qRT-PCR.

Supplementary Figure 2 **(A)** The size of the testes obtained from wild-type and GRTH knock-in (KI) mice. The testes from homozygous KI mice are smaller compared with those from wild-type mice. **(B)** Mean testis–body weight ratio of WT and GRTH KI mice (*n* = 10). **P* < 0.05.

Supplementary Figure 3 Successful chromatoid body isolation from wild-type and knock-in mice was validated with DDX4 antibody immunofluorescence. Arrows indicate CBs showing DDX4 expression in green.

Supplementary Figure 4 Western blot analysis showing BMAL1, CLOCK, YBX3, PRM1, and PRM2 expression in the CB samples obtained from wild-type and knock-in mice. MIWI was used as a loading control for CB proteins.

Supplementary Table 5 Comparison of gene expression (fold change) in germ cells with transcript abundance and fold change from chromatoid bodies (CBs) obtained from WT and KI mice. **(A)** Downregulated/Upregulated genes in germ cells in comparison with a decrease in abundance of genes in CB. **(B)** Upregulated genes in germ cells in comparison with an increase in abundance of genes in CB.

Supplementary Table 6 Differential abundance of transcripts with ensemble ID, total counts, *P*-value, false discovery rate, and fold change between chromatoid bodies of wild-type and knock-in groups.

Supplementary Table 7 Gene Ontology enrichment analysis showing functional classes, biological process, cellular component, and molecular function with group ID, category, log *P*-value, and list of gene symbols.

Supplementary Figure 8 Comparison of gene expression in germ cells with transcript abundance from chromatoid bodies (CBs) obtained from wild-type and knock-in mice. **(A)** Venn diagram showing downregulated genes in germ cells and a decrease in the abundance of genes in CB. **(B)** Venn diagram showing upregulated genes in germ cells and an increase in abundance of genes in CB.

Supplementary Figure 9 Western blot analysis showing GRTH expression in input samples and GRTH immunoprecipitation samples from the chromatoid bodies obtained from wild-type and knock-in mice.
