# Supplementary material for: Role of Phosphorylated Gonadotropin-Regulated Testicular RNA Helicase (GRTH/DDX25) in the Regulation of Germ Cell Specific mRNAs in Chromatoid Bodies During Spermatogenesis
Source: Front Cell Dev Biol. 2020 Dec 23;8:580019. doi: 10.3389/fcell.2020.580019 (PMC7786181; doi:10.3389/fcell.2020.580019)
Supplement: Supplementary Table 1 — List of primers used for genotyping and validation of differentially enriched transcripts using qRT-PCR. [file Data_Sheet_1.zip › Supplementary files/Supplementary Table 1.DOCX]

**Supplementary Table 1:**

List of primers used for genotyping and validation of DEGs using qRT-PCR

| **Primer Name** | **Primer Sequence 5’---3’** |
| --- | --- |
| **Geno F1** | AGA ACG GCA TCA AGG TGA AC |
| **Geno R1** | GCC TCC CCA CAG TAA CGA C |
| **Geno F2** | GAT ACC TAA GTT GGC TAG GTA TCC TGA G |
| **Geno R2** | ATA GTT GCC CAA GCT GCT ACC CCA GTT CCA |
| **Grth F** | CAG ATT ATA ATT GGC ACT CCT G |
| **Grth R** | CAT TCG GAG GGT AGA GCT CT |
| **Tnp1 F** | GAC TCA TGG CAT GAG GAG AG |
| **Tnp1 R** | ATT GCG ACT TGC ATC ATC GC |
| **Tnp2 F** | GCT CTA GCT CCA GCC CCA GC |
| **Tnp2 R** | CTT GTA TCT TCG CCC TGA G |
| **Prm1 F** | AGA TAC CGA TGC TGC CGC AG |
| **Prm1 R** | ACG GCA GCA TCT TCG CCT CCT C |
| **Prm2 F** | GGC AAG GGC TGA GCC CAG AGC |
| **Prm2 R** | TCG GGA TCT TCT GCA GCC TCT GC |
| **Tssk3 F** | GAG GGC CAG AAG AGT TTA TCC A |
| **Tssk3 R** | CAT CTG CTG ACT CCA GCA TCT |
| **Tssk6 F** | CCG CGA AGT CGC CGC GCG CCA CTG |
| **Tssk6 R** | TCT CTC TTC TCT TTG CGC CCC TCC |
| **Spem1 F** | GGT GAC CCT GCT CTG GAG C |
| **Spem1 R** | CAC AGG GTC CAT GGT ACA TCG A |
| **Upf2 F** | GAT TGG GAA TGG AGG TTA ATC A |
| **Upf2 R** | ACT TGG AGA CCC ATC AGG |
| **tAce F** | ATGGGCCACATCCAGTATTTCATGCA |
| **tAce R** | GATCTTGTCGAGGGCCATCTTCATTAG |
| **Eef1a1 F** | GTC TGG CGA TGC TGC CAT TG |
| **Eef1a1 R** | GCA AAG CGA CCA AGT GGA G |
| **PPP1cc F** | AGA TAT GTG GTG ACA TCC ACG |
| **PPP1cc R** | GCA GAT TGT CTC CAG GGA CTG |
| **Ybx3 F** | AGT TCT CTG GGG CCA GGA A |
| **Ybx3 R** | ATC TCA CCA GCC TGC ATT CTG |
| **H2al1m F** | GTC GCC TGA GCT CTT CCG CA |
| **H2al1m R** | GTG GGC CAC CTC ACC AGC CA |
| **Dctn2 F** | GAG AAG ATC AAG ACG ACA GTG |
| **Dctn2 R** | CAG CAG TAA GCG CTT AGC T |
| **Dync1h1 F** | GAA GGC CAG CGT GGT GAC CT |
| **Dync1h1 R** | GCT GCG AGG ATC TTC CTT TGT AGC |
| **Pabpc1 F** | ATG CGC AGA AAG CTG TGG |
| **Pabpc1 R** | GAT CCT ATC TTG CTT CAT CTG C |
| **Tent5b F** | GAT CGT GCA GGT GGT CCG CA |
| **Tent5b R** | GCA CGT GGC TGG CGG CTG |
| **DDX4 F** | GTA CTG TCA GAC GCT CAA CAG G |
| **DDX4 R** | GCT ACT GAA GCT GGG AGG CA |
