# Supplementary material for: Role of Phosphorylated Gonadotropin-Regulated Testicular RNA Helicase (GRTH/DDX25) in the Regulation of Germ Cell Specific mRNAs in Chromatoid Bodies During Spermatogenesis
Source: Front Cell Dev Biol. 2020 Dec 23;8:580019. doi: 10.3389/fcell.2020.580019 (PMC7786181; doi:10.3389/fcell.2020.580019)
Supplement: Supplementary Table 1 — List of primers used for genotyping and validation of differentially enriched transcripts using qRT-PCR. [file Data_Sheet_1.zip › Supplementary files/Supplementary Table 5.docx]

**Supplementary Table 5:** Comparison of gene expression (fold change) in germ cells with transcript abundance and fold change from chromatoid bodies (CBs) obtained from WT and KI mice. (A) Downregulated/Upregulated genes in germ cells in comparison with a decrease in abundance of genes in CB. (B) Upregulated genes in germ cells in comparison with an increase in abundance of genes in CB.

**A.**

| **Genes** | Fold change in CB | Fold change in Germ cells |
| --- | --- | --- |
| Ddx25 | -9.75E+00 | -8.15E+01 |
| Prokr1 | -8.66E+00 | -1.44E+01 |
| Lrrc57 | -4.30E+00 | -1.11E+01 |
| 1700008P02Rik | -5.76E+00 | -1.08E+01 |
| Gm5893 | -3.46E+00 | -1.03E+01 |
| Wbscr25 | -3.76E+00 | -9.86E+00 |
| Prm2 | -3.54E+00 | -9.61E+00 |
| 1700012A03Rik | -3.77E+00 | -9.42E+00 |
| Azin2 | -3.11E+00 | -9.36E+00 |
| Ppp2r2b | -2.42E+00 | -8.82E+00 |
| Ubqlnl | -5.57E+00 | -8.76E+00 |
| Car2 | -2.76E+00 | -8.75E+00 |
| 1700009J07Rik | -3.17E+00 | -8.72E+00 |
| Fam131b | -7.08E+00 | -8.21E+00 |
| Acte1 | -3.40E+00 | -8.01E+00 |
| Tmem56 | -2.94E+00 | -7.82E+00 |
| Tssk6 | -2.87E+00 | -7.81E+00 |
| 1700016H13Rik | -1.80E+00 | -7.58E+00 |
| Klk1b8 | -2.85E+00 | -7.47E+00 |
| 1700029B22Rik | -2.48E+00 | -7.39E+00 |
| Tppp2 | -5.36E+00 | -7.35E+00 |
| Spem2 | -3.33E+00 | -7.24E+00 |
| Prm1 | -3.36E+00 | -6.98E+00 |
| 1700027A15Rik | -2.45E+00 | -6.87E+00 |
| Smcp | -2.46E+00 | -6.74E+00 |
| Spata18 | -2.55E+00 | -6.61E+00 |
| Lexm | -3.40E+00 | -6.59E+00 |
| Cabs1 | -3.84E+00 | -6.58E+00 |
| Fscn3 | -3.06E+00 | -6.43E+00 |
| Spem1 | -3.99E+00 | -6.21E+00 |
| Odf1 | -2.83E+00 | -5.86E+00 |
| 4933411K16Rik | -2.82E+00 | -5.80E+00 |
| Oaz3 | -3.20E+00 | -5.62E+00 |
| Fam71f2 | -1.90E+00 | -5.56E+00 |
| Reep6 | -2.14E+00 | -5.41E+00 |
| Nudt16l2 | -3.13E+00 | -5.39E+00 |
| St6galnac2 | -2.63E+00 | -5.28E+00 |
| Nnmt | -1.45E+01 | -5.28E+00 |
| 4921507P07Rik | -2.65E+00 | -5.21E+00 |
| Spata3 | -2.43E+00 | -5.16E+00 |
| Spata32 | -2.29E+00 | -5.16E+00 |
| 4930428D20Rik | -3.09E+00 | -5.10E+00 |
| Glrx2 | -1.55E+00 | -5.07E+00 |
| Mical3 | -1.57E+00 | -4.91E+00 |
| 1700096J18Rik | -6.74E+00 | -4.89E+00 |
| 1700042G07Rik | -2.65E+00 | -4.87E+00 |
| Tnp2 | -2.33E+00 | -4.77E+00 |
| Oxct2b | -2.24E+00 | -4.77E+00 |
| Clmn | -2.56E+00 | -4.74E+00 |
| 1700015G11Rik | -2.30E+00 | -4.68E+00 |
| Tssk3 | -2.63E+00 | -4.62E+00 |
| Gapdhs | -1.97E+00 | -4.47E+00 |
| 1700031M16Rik | -2.55E+00 | -4.43E+00 |
| Hspa1l | -2.85E+00 | -4.40E+00 |
| Prkcq | -1.91E+00 | -4.39E+00 |
| Lelp1 | -1.98E+00 | -4.38E+00 |
| Gm6760 | -2.52E+00 | -4.38E+00 |
| Phospho1 | -2.08E+00 | -4.36E+00 |
| Dnajb8 | -2.22E+00 | -4.35E+00 |
| 1700048M11Rik | -1.47E+01 | -4.33E+00 |
| 4930571K23Rik | -2.45E+00 | -4.33E+00 |
| Atl3 | -2.44E+00 | -4.24E+00 |
| Gsg1 | -2.39E+00 | -4.15E+00 |
| Tnp1 | -2.90E+00 | -4.14E+00 |
| Prkar2a | -2.01E+00 | -4.04E+00 |
| Spatc1 | -1.93E+00 | -3.96E+00 |
| Prm3 | -1.78E+00 | -3.88E+00 |
| 1700001O22Rik | -1.99E+00 | -3.86E+00 |
| Ubl4b | -1.87E+00 | -3.84E+00 |
| Gm11780 | -2.67E+00 | -3.84E+00 |
| Cdrt4 | -2.10E+00 | -3.82E+00 |
| 2810408A11Rik | -1.90E+00 | -3.79E+00 |
| Iqcf3 | -2.11E+00 | -3.75E+00 |
| 1700009N14Rik | -1.73E+00 | -3.73E+00 |
| 1700024P04Rik | -3.17E+00 | -3.72E+00 |
| 1700034E13Rik | -2.63E+00 | -3.70E+00 |
| Ccdc91 | -1.83E+00 | -3.68E+00 |
| Csnk1g2 | -2.18E+00 | -3.65E+00 |
| Akap4 | -2.71E+00 | -3.65E+00 |
| Cdiptos | -1.62E+00 | -3.65E+00 |
| Pdzd8 | -1.84E+00 | -3.63E+00 |
| Ube2k | -1.81E+00 | -3.60E+00 |
| Gm3409 | -4.52E+00 | -3.59E+00 |
| 4930503B20Rik | -1.87E+00 | -3.54E+00 |
| Gm136 | -2.12E+00 | -3.48E+00 |
| Dbil5 | -2.28E+00 | -3.44E+00 |
| Lipe | -2.09E+00 | -3.40E+00 |
| Mfap3l | -2.10E+00 | -3.40E+00 |
| BB014433 | -2.01E+00 | -3.37E+00 |
| Isg20l2 | -1.79E+00 | -3.37E+00 |
| Spz1 | -2.08E+00 | -3.37E+00 |
| Fam187b | -2.71E+00 | -3.35E+00 |
| Oxct2a | -3.27E+00 | -3.35E+00 |
| Acsl1 | -1.78E+00 | -3.28E+00 |
| Actl11 | -1.66E+00 | -3.27E+00 |
| 1700034I23Rik | -2.06E+00 | -3.25E+00 |
| Rnf133 | -2.14E+00 | -3.24E+00 |
| Txndc2 | -2.01E+00 | -3.24E+00 |
| Lrrc8b | -1.55E+00 | -3.21E+00 |
| AF366264 | -2.70E+00 | -3.17E+00 |
| Prkag2os1 | -4.19E+00 | -3.17E+00 |
| Bcl2l14 | -1.70E+00 | -3.16E+00 |
| 1700029H14Rik | -1.88E+00 | -3.14E+00 |
| Fam243 | -1.66E+00 | -3.14E+00 |
| Chl1 | -2.01E+00 | -3.13E+00 |
| H1fnt | -1.97E+00 | -3.13E+00 |
| Ccdc54 | -1.69E+00 | -3.10E+00 |
| Syce2 | -1.71E+00 | -3.09E+00 |
| Hk1 | -1.62E+00 | -3.00E+00 |
| Ppm1a | -1.59E+00 | -3.00E+00 |
| Naa60 | -2.30E+00 | -2.91E+00 |
| Atp1b3 | -1.93E+00 | -2.91E+00 |
| 1700022A22Rik | -1.66E+00 | -2.90E+00 |
| Prss51 | -3.08E+00 | -2.90E+00 |
| Rnf138 | -1.59E+00 | -2.83E+00 |
| 1110017D15Rik | -2.46E+00 | -2.82E+00 |
| Nsun4 | -1.80E+00 | -2.82E+00 |
| Ankef1 | -1.96E+00 | -2.78E+00 |
| Speer3 | -5.18E+00 | -2.77E+00 |
| Satl1 | -2.37E+00 | -2.76E+00 |
| Iqcf4 | -2.05E+00 | -2.67E+00 |
| 2610318N02Rik | -1.65E+00 | -2.67E+00 |
| Akap1 | -1.69E+00 | -2.62E+00 |
| Acsbg2 | -1.62E+00 | -2.62E+00 |
| Hmgb4 | -2.36E+00 | -2.62E+00 |
| Spert | -2.05E+00 | -2.71E+00 |
| Chpt1 | -1.69E+00 | -2.61E+00 |
| Wdr64 | -1.89E+00 | -2.61E+00 |
| Actg2 | -2.18E+00 | -2.60E+00 |
| Ly6g6c | -2.94E+00 | -2.59E+00 |
| Gm6370 | -3.73E+00 | -2.59E+00 |
| Camk4 | -1.98E+00 | -2.58E+00 |
| Nt5c1b | -1.69E+00 | -2.53E+00 |
| Cdc14a | -1.56E+00 | -2.52E+00 |
| Gm9758 | -5.82E+00 | -2.51E+00 |
| Fam71d | -1.84E+00 | -2.47E+00 |
| Ccdc70 | -1.57E+00 | -2.46E+00 |
| 4933402C06Rik | -2.67E+00 | -2.46E+00 |
| Tex50 | -1.66E+00 | -2.44E+00 |
| Eef1d | -1.58E+00 | -2.43E+00 |
| Slfnl1 | -1.72E+00 | -2.42E+00 |
| 1700019M22Rik | -1.60E+00 | -2.42E+00 |
| Bag1 | -1.55E+00 | -2.41E+00 |
| Sppl2c | -1.61E+00 | -2.39E+00 |
| Pmfbp1 | -1.68E+00 | -2.38E+00 |
| Cst8 | -1.67E+00 | -2.37E+00 |
| Prr30 | -1.60E+00 | -2.36E+00 |
| Tasp1 | -1.71E+00 | -2.35E+00 |
| Cnbd1 | -1.85E+00 | -2.35E+00 |
| Zfp239 | -3.26E+00 | -2.33E+00 |
| Tex35 | -2.38E+00 | -2.31E+00 |
| Klhl10 | -1.51E+00 | -2.31E+00 |
| Gm10354 | -1.10E+01 | -2.30E+00 |
| Gm3415 | -2.07E+01 | -2.30E+00 |
| Gm17019 | -8.18E+00 | -2.29E+00 |
| Chrna5 | -5.49E+00 | -2.28E+00 |
| Otub2 | -2.03E+00 | -2.27E+00 |
| Bzw1 | -1.65E+00 | -2.27E+00 |
| Dnajc5b | -1.85E+00 | -2.26E+00 |
| Gm3404 | -7.74E+00 | -2.25E+00 |
| Pkhd1l1 | -2.64E+00 | -2.25E+00 |
| Cdk16 | -2.08E+00 | -2.24E+00 |
| Chn2 | -2.11E+00 | -2.23E+00 |
| Ace | -1.57E+00 | -2.22E+00 |
| Fam166a | -1.99E+00 | -2.22E+00 |
| Tipin | -1.96E+00 | -2.21E+00 |
| Tmem156 | -2.96E+00 | -2.20E+00 |
| Ankrd53 | -2.14E+00 | -2.20E+00 |
| Dlgap4 | -1.79E+00 | -2.20E+00 |
| Wwp2 | -1.82E+00 | -2.19E+00 |
| Sh3rf2 | -2.03E+00 | -2.17E+00 |
| 1700013G24Rik | -1.66E+00 | -2.15E+00 |
| Cdv3 | -1.57E+00 | -2.15E+00 |
| Actl9 | -1.61E+00 | -2.13E+00 |
| Tle3 | -2.30E+00 | -2.13E+00 |
| Gabarapl1 | -1.56E+00 | -2.12E+00 |
| 1700092M07Rik | -1.73E+00 | -2.11E+00 |
| Nalcn | -3.20E+00 | -2.11E+00 |
| Gm3402 | -5.40E+00 | -2.10E+00 |
| Yod1 | -1.60E+00 | -2.09E+00 |
| BC048671 | -2.07E+00 | -2.09E+00 |
| Nipsnap3a | -2.40E+00 | -2.09E+00 |
| Tssk2 | -1.53E+00 | -2.07E+00 |
| Proca1 | -1.54E+00 | -2.05E+00 |
| 1700095B10Rik | -2.01E+00 | -2.05E+00 |
| Speer4b | -4.29E+00 | -2.04E+00 |
| Dydc1 | -2.11E+00 | -2.03E+00 |
| Akap12 | -1.74E+00 | -2.02E+00 |
| Grip1 | -1.57E+00 | -2.02E+00 |
| Oser1 | -1.62E+00 | -2.01E+00 |
| Suv39h2 | -2.30E+00 | -1.99E+00 |
| Eef1g | -1.69E+00 | -1.99E+00 |
| 1700003F12Rik | -1.76E+00 | -1.99E+00 |
| Aldoart2 | -1.56E+00 | -1.97E+00 |
| Gm6408 | -5.25E+00 | -1.96E+00 |
| Gm20611 | -2.91E+00 | -1.93E+00 |
| Ttll10 | -1.58E+00 | -1.92E+00 |
| Iqcm | -1.61E+00 | -1.92E+00 |
| Tepp | -3.03E+00 | -1.92E+00 |
| Izumo3 | -1.51E+00 | -1.91E+00 |
| Aknad1 | -1.88E+00 | -1.90E+00 |
| Spn-ps | -2.45E+00 | -1.90E+00 |
| Gdpd1 | -1.75E+00 | -1.89E+00 |
| Samd4 | -1.59E+00 | -1.89E+00 |
| Gm5512 | -1.76E+00 | -1.88E+00 |
| Dnajb7 | -1.70E+00 | -1.88E+00 |
| Rmnd1 | -2.28E+00 | -1.88E+00 |
| Ntan1 | -7.34E+00 | -1.88E+00 |
| Ilrun | -1.88E+00 | -1.88E+00 |
| Smok3b | -3.65E+00 | -1.87E+00 |
| Smok3c | -3.46E+00 | -1.85E+00 |
| 4933404G15Rik | -2.36E+00 | -1.85E+00 |
| 4933402J07Rik | -1.58E+00 | -1.84E+00 |
| Cfhr1 | -2.73E+00 | -1.84E+00 |
| Spanxn4 | -1.71E+00 | -1.84E+00 |
| Fam227a | -1.99E+00 | -1.82E+00 |
| Speer4e | -3.27E+00 | -1.81E+00 |
| Clk4 | -2.23E+00 | -1.80E+00 |
| Prdx4 | -2.77E+00 | -1.78E+00 |
| Irgc1 | -1.51E+00 | -1.77E+00 |
| Trabd2b | -9.21E+00 | -1.77E+00 |
| Zfand3 | -1.52E+00 | -1.76E+00 |
| Speer4f1 | -3.61E+00 | -1.76E+00 |
| Spata48 | -2.02E+00 | -1.75E+00 |
| Ropn1 | -1.86E+00 | -1.75E+00 |
| Hsdl2 | -1.67E+00 | -1.75E+00 |
| Vwa3a | -1.58E+00 | -1.75E+00 |
| 4930442L01Rik | -2.00E+00 | -1.75E+00 |
| Rasa3 | -2.20E+00 | -1.73E+00 |
| Wbp2nl | -2.21E+00 | -1.73E+00 |
| Spata20 | -1.56E+00 | -1.72E+00 |
| Rgsl1 | -1.73E+00 | -1.72E+00 |
| 4931408C20Rik | -3.10E+00 | -1.71E+00 |
| Cep112 | -1.54E+00 | -1.69E+00 |
| Sorbs3 | -2.35E+00 | -1.68E+00 |
| Phactr1 | -2.07E+00 | -1.68E+00 |
| Tex24 | -3.34E+00 | -1.68E+00 |
| Glul | -1.65E+00 | -1.68E+00 |
| Paip2 | -1.54E+00 | -1.67E+00 |
| Spaca9 | -1.91E+00 | -1.67E+00 |
| Cabyr | -1.58E+00 | -1.66E+00 |
| 4930572O13Rik | -4.36E+00 | -1.66E+00 |
| Lemd1 | -2.29E+00 | -1.65E+00 |
| Ccdc28a | -2.03E+00 | -1.64E+00 |
| Shcbp1 | -1.65E+00 | -1.63E+00 |
| Eif4e | -1.56E+00 | -1.63E+00 |
| Gm9839 | -3.16E+00 | -1.63E+00 |
| Srxn1 | -2.11E+00 | -1.62E+00 |
| Hsfy2 | -1.84E+00 | -1.62E+00 |
| Erich3 | -1.60E+00 | -1.61E+00 |
| Gm5415 | -2.81E+00 | -1.60E+00 |
| 4930563J15Rik | -1.82E+00 | -1.60E+00 |
| Slc35a5 | -1.81E+00 | -1.60E+00 |
| R3hcc1 | -2.09E+00 | -1.59E+00 |
| Ydjc | -3.39E+00 | -1.58E+00 |
| Fam83e | -1.78E+00 | -1.58E+00 |
| Nup35 | -2.22E+00 | -1.57E+00 |
| 1700011L22Rik | -2.06E+00 | -1.56E+00 |
| Prdm16 | -2.88E+00 | -1.56E+00 |
| 1700012B07Rik | -1.56E+00 | -1.56E+00 |
| Dcaf1 | -1.61E+00 | -1.55E+00 |
| Antxrl | -1.71E+00 | -1.55E+00 |
| Gm4787 | -1.57E+00 | -1.55E+00 |
| Il1rap | -3.51E+00 | -1.55E+00 |
| Pigg | -2.27E+00 | -1.55E+00 |
| Gm21190 | -1.07E+01 | -1.54E+00 |
| Wscd2 | -2.03E+00 | -1.54E+00 |
| Trp53tg5 | -2.43E+00 | -1.53E+00 |
| Tmem210 | -2.20E+00 | -1.52E+00 |
| Selenov | -1.84E+00 | -1.52E+00 |
| Ccdc150 | -1.64E+00 | -1.51E+00 |
| 4930444G20Rik | -2.50E+00 | -1.50E+00 |
| Genes | Fold change in CB | Fold change in Germ cells |
| Tmem184c | -2.22E+00 | 1.54E+00 |
| 2810006K23Rik | -3.82E+00 | 1.62E+00 |
| Akap6 | -2.13E+00 | 1.74E+00 |
| Ptgfrn | -6.51E+00 | 1.77E+00 |
| Zfp72 | -2.22E+00 | 1.95E+00 |
| Ptn | -3.25E+01 | 2.01E+00 |
| Fmo2 | -1.44E+01 | 2.41E+00 |
| Ccr2 | -4.45E+00 | 2.73E+00 |

**B.**

| Genes | Fold change in CB | Fold change in Germ cells |
| --- | --- | --- |
| Lrrc47 | 1.94E+00 | 1.55E+00 |
| Rnf214 | 1.69E+00 | 1.61E+00 |
| Plxnb1 | 3.00E+00 | 1.68E+00 |
| Bhmt | 6.60E+00 | 1.72E+00 |
| Lamb2 | 6.79E+00 | 1.74E+00 |
| Insl3 | 4.23E+00 | 1.78E+00 |
| Csk | 4.62E+00 | 1.86E+00 |
| Col9a3 | 3.28E+00 | 2.05E+00 |
| Dixdc1 | 1.62E+00 | 2.29E+00 |
| Tarsl2 | 2.44E+00 | 2.45E+00 |
| Kcnh8 | 3.47E+00 | 2.48E+00 |
| Olfr1372-ps1 | 2.89E+00 | 2.70E+00 |
| Slc26a11 | 6.89E+00 | 2.93E+00 |
| Plekhg2 | 2.38E+00 | 2.97E+00 |
| Dnah11 | 3.82E+00 | 3.26E+00 |
| Esrrb | 3.44E+00 | 5.21E+00 |
| Kcnh4 | 7.26E+00 | 1.09E+01 |
| Asprv1 | 7.12E+00 | 5.90E+01 |
