# Supplementary material for: Role of Phosphorylated Gonadotropin-Regulated Testicular RNA Helicase (GRTH/DDX25) in the Regulation of Germ Cell Specific mRNAs in Chromatoid Bodies During Spermatogenesis
Source: Front Cell Dev Biol. 2020 Dec 23;8:580019. doi: 10.3389/fcell.2020.580019 (PMC7786181; doi:10.3389/fcell.2020.580019)
Supplement: Supplementary Table 1 — List of primers used for genotyping and validation of differentially enriched transcripts using qRT-PCR. [file Data_Sheet_1.zip › Supplementary files/Supplementary Table 6.docx]

**Supplementary Table 6:** Differential abundance of transcripts with ensemble ID, total counts, P-value, false discovery rate, and fold change between chromatoid bodies of wild-type and knock-in groups.

| **Gene name** | **Total counts** | **P-value** | **FDR** | **Fold change** |  |
| --- | --- | --- | --- | --- | --- |
| Gm10354 | 2.11E+03 | 4.93E-44 | 1.09E-39 | -1.10E+01 |  |
| Prm2 | 1.40E+04 | 9.26E-44 | 1.09E-39 | -3.54E+00 |  |
| Odf1 | 5.42E+03 | 1.50E-38 | 1.17E-34 | -2.83E+00 |  |
| St6galnac2 | 7.14E+03 | 4.64E-34 | 2.72E-30 | -2.63E+00 |  |
| Tex24 | 2.88E+03 | 2.55E-30 | 1.19E-26 | -3.34E+00 |  |
| Tnp1 | 8.71E+03 | 8.07E-30 | 3.15E-26 | -2.90E+00 |  |
| Cabs1 | 4.10E+03 | 1.11E-29 | 3.73E-26 | -3.84E+00 |  |
| Gm8251 | 1.32E+04 | 1.02E-25 | 2.98E-22 | -2.41E+00 |  |
| 4933411K16Rik | 2.91E+03 | 1.92E-23 | 5.00E-20 | -2.82E+00 |  |
| 1700007E05Rik | 2.04E+03 | 4.73E-22 | 1.11E-18 | -4.51E+00 |  |
| Prm1 | 3.80E+03 | 5.81E-22 | 1.24E-18 | -3.36E+00 |  |
| Spem1 | 1.23E+03 | 1.18E-21 | 2.31E-18 | -3.99E+00 |  |
| Wbscr25 | 1.64E+03 | 4.15E-21 | 7.48E-18 | -3.76E+00 |  |
| Gm3415 | 4.42E+02 | 1.63E-20 | 2.73E-17 | -2.07E+01 |  |
| Ubqlnl | 1.08E+03 | 2.08E-20 | 3.24E-17 | -5.57E+00 |  |
| Hspa1l | 2.83E+03 | 3.72E-20 | 5.45E-17 | -2.85E+00 |  |
| Clmn | 1.46E+03 | 9.32E-20 | 1.28E-16 | -2.56E+00 |  |
| Speer3 | 6.14E+02 | 8.75E-19 | 1.14E-15 | -5.18E+00 |  |
| Gm17019 | 1.07E+03 | 1.14E-18 | 1.40E-15 | -8.18E+00 |  |
| Akap4 | 7.27E+03 | 1.40E-18 | 1.58E-15 | -2.71E+00 |  |
| Dbil5 | 3.39E+03 | 1.42E-18 | 1.58E-15 | -2.28E+00 |  |
| Gm21149 | 1.48E+03 | 5.26E-18 | 5.60E-15 | -8.90E+00 |  |
| AF366264 | 2.69E+03 | 6.03E-18 | 6.15E-15 | -2.70E+00 |  |
| Gm3409 | 5.58E+02 | 7.37E-18 | 7.19E-15 | -4.52E+00 |  |
| Chl1 | 3.67E+03 | 3.55E-17 | 3.32E-14 | -2.01E+00 |  |
| Fam205a3 | 1.91E+03 | 2.25E-16 | 2.03E-13 | -3.73E+00 |  |
| Gm22513 | 7.74E+04 | 3.16E-16 | 2.74E-13 | 2.59E+00 |  |
| Rnu1b1 | 2.75E+04 | 3.83E-16 | 3.20E-13 | 2.86E+00 |  |
| 4931408C20Rik | 1.71E+03 | 6.91E-16 | 5.58E-13 | -3.10E+00 |  |
| Tppp2 | 6.72E+02 | 7.32E-16 | 5.71E-13 | -5.36E+00 |  |
| Speer4c | 1.32E+03 | 1.62E-15 | 1.22E-12 | -6.61E+00 |  |
| Gm6611 | 9.13E+02 | 1.92E-15 | 1.41E-12 | -4.56E+00 |  |
| Ankef1 | 3.77E+03 | 4.48E-15 | 3.18E-12 | -1.96E+00 |  |
| 4932415M13Rik | 2.17E+03 | 5.28E-15 | 3.64E-12 | -2.83E+00 |  |
| Gm9839 | 1.28E+03 | 6.58E-15 | 4.40E-12 | -3.16E+00 |  |
| Gm24830 | 2.56E+04 | 1.32E-14 | 8.56E-12 | 2.51E+00 |  |
| Gm7361 | 1.23E+03 | 1.41E-14 | 8.94E-12 | -3.33E+00 |  |
| Gm8703 | 4.90E+02 | 2.12E-14 | 1.31E-11 | -3.65E+00 |  |
| Fancd2os | 1.29E+03 | 3.90E-14 | 2.34E-11 | -3.37E+00 |  |
| Gm5152 | 4.68E+02 | 8.43E-14 | 4.94E-11 | -9.93E+00 |  |
| Chn2 | 2.06E+03 | 1.69E-13 | 9.67E-11 | -2.11E+00 |  |
| Upf2 | 2.93E+03 | 2.42E-13 | 1.35E-10 | -2.01E+00 |  |
| Oaz3 | 4.37E+03 | 3.04E-13 | 1.66E-10 | -3.20E+00 |  |
| Gm4942 | 1.60E+03 | 3.23E-13 | 1.72E-10 | -2.83E+00 |  |
| Gm26232 | 3.38E+04 | 3.93E-13 | 2.05E-10 | 2.31E+00 |  |
| Rnu1b6 | 3.40E+04 | 8.69E-13 | 4.43E-10 | 2.29E+00 |  |
| Gm9758 | 7.02E+02 | 1.30E-12 | 6.50E-10 | -5.82E+00 |  |
| Catsper1 | 1.48E+03 | 1.36E-12 | 6.65E-10 | -2.57E+00 |  |
| Hmgb4 | 3.14E+03 | 1.44E-12 | 6.89E-10 | -2.36E+00 |  |
| Tmem56 | 8.07E+02 | 3.26E-12 | 1.51E-09 | -2.94E+00 |  |
| Speer4b | 1.03E+03 | 3.28E-12 | 1.51E-09 | -4.29E+00 |  |
| Trim59 | 2.32E+03 | 3.48E-12 | 1.57E-09 | -2.81E+00 |  |
| Gm10220 | 6.88E+02 | 4.18E-12 | 1.85E-09 | -3.24E+00 |  |
| Gm1527 | 1.23E+03 | 6.36E-12 | 2.76E-09 | -3.63E+00 |  |
| Gm23804 | 2.61E+03 | 8.08E-12 | 3.41E-09 | 4.23E+00 |  |
| Tssk3 | 1.31E+03 | 8.15E-12 | 3.41E-09 | -2.63E+00 |  |
| Atp1b3 | 2.30E+03 | 9.27E-12 | 3.81E-09 | -1.93E+00 |  |
| Spem2 | 9.85E+02 | 9.63E-12 | 3.89E-09 | -3.33E+00 |  |
| Gm6647 | 4.70E+02 | 1.49E-11 | 5.87E-09 | -4.62E+00 |  |
| Rnu3b2 | 5.06E+04 | 1.50E-11 | 5.87E-09 | 2.58E+00 |  |
| 4930596D02Rik | 6.65E+02 | 1.96E-11 | 7.55E-09 | -3.18E+00 |  |
| 4930571K23Rik | 3.44E+03 | 2.56E-11 | 9.68E-09 | -2.45E+00 |  |
| 1700012A03Rik | 5.39E+02 | 2.64E-11 | 9.81E-09 | -3.77E+00 |  |
| Smcp | 1.37E+04 | 2.97E-11 | 1.09E-08 | -2.46E+00 |  |
| Rnu1b2 | 3.34E+04 | 3.23E-11 | 1.16E-08 | 2.57E+00 |  |
| Cftr | 2.84E+03 | 4.35E-11 | 1.54E-08 | -2.25E+00 |  |
| Rnu3b1 | 5.09E+04 | 4.52E-11 | 1.58E-08 | 2.54E+00 |  |
| Lrrc57 | 7.32E+02 | 5.29E-11 | 1.82E-08 | -4.30E+00 |  |
| Speer4f2 | 7.24E+02 | 5.69E-11 | 1.93E-08 | -5.62E+00 |  |
| Fam205a4 | 2.01E+03 | 7.39E-11 | 2.47E-08 | -3.18E+00 |  |
| Spata18 | 1.49E+03 | 7.69E-11 | 2.54E-08 | -2.55E+00 |  |
| Prdx4 | 8.71E+02 | 8.12E-11 | 2.64E-08 | -2.77E+00 |  |
| 1700031M16Rik | 9.83E+02 | 8.95E-11 | 2.87E-08 | -2.55E+00 |  |
| Mfap3l | 1.68E+03 | 1.20E-10 | 3.78E-08 | -2.10E+00 |  |
| Gm5862 | 6.55E+02 | 1.26E-10 | 3.94E-08 | -3.90E+00 |  |
| 1700034I23Rik | 2.15E+03 | 1.37E-10 | 4.21E-08 | -2.06E+00 |  |
| 4930548H24Rik | 5.13E+02 | 1.42E-10 | 4.32E-08 | -3.41E+00 |  |
| Snord15b | 1.83E+03 | 1.76E-10 | 5.29E-08 | 3.66E+00 |  |
| 4933421I07Rik | 6.21E+02 | 1.81E-10 | 5.37E-08 | -3.53E+00 |  |
| Speer4d | 9.88E+02 | 2.64E-10 | 7.73E-08 | -5.76E+00 |  |
| Atl3 | 1.64E+03 | 2.74E-10 | 7.91E-08 | -2.44E+00 |  |
| Gm6657 | 7.05E+02 | 2.77E-10 | 7.91E-08 | -2.54E+00 |  |
| Gm20594 | 1.11E+04 | 3.60E-10 | 1.01E-07 | 2.54E+00 |  |
| Fam187b | 9.41E+02 | 3.67E-10 | 1.01E-07 | -2.71E+00 |  |
| Ybx3 | 5.89E+03 | 3.69E-10 | 1.01E-07 | 1.90E+00 |  |
| Iqcf3 | 2.31E+03 | 3.73E-10 | 1.01E-07 | -2.11E+00 |  |
| Gm5361 | 1.03E+03 | 3.88E-10 | 1.05E-07 | -2.99E+00 |  |
| 1110017D15Rik | 9.82E+02 | 4.46E-10 | 1.19E-07 | -2.46E+00 |  |
| Mtbp | 1.14E+03 | 5.22E-10 | 1.37E-07 | -2.25E+00 |  |
| Spz1 | 3.95E+03 | 7.53E-10 | 1.96E-07 | -2.08E+00 |  |
| Dram1 | 6.90E+02 | 8.07E-10 | 2.08E-07 | -2.82E+00 |  |
| Tmem184c | 2.63E+03 | 8.43E-10 | 2.15E-07 | -2.22E+00 |  |
| 4933404G15Rik | 1.83E+03 | 9.82E-10 | 2.47E-07 | -2.36E+00 |  |
| Iqca | 1.19E+03 | 1.01E-09 | 2.49E-07 | -2.44E+00 |  |
| Gm20557 | 5.68E+02 | 1.01E-09 | 2.49E-07 | -3.64E+00 |  |
| Gm6650 | 3.34E+02 | 1.11E-09 | 2.70E-07 | -7.51E+00 |  |
| Nsun4 | 3.13E+03 | 1.23E-09 | 2.98E-07 | -1.80E+00 |  |
| Gsg1 | 3.07E+03 | 1.44E-09 | 3.43E-07 | -2.39E+00 |  |
| AL824709.1 | 1.54E+03 | 1.54E-09 | 3.64E-07 | -2.82E+00 |  |
| Gm5798 | 3.53E+02 | 1.85E-09 | 4.34E-07 | -3.92E+00 |  |
| Gm4808 | 9.20E+02 | 2.17E-09 | 5.04E-07 | -3.00E+00 |  |
| Gm24616 | 1.30E+03 | 2.29E-09 | 5.25E-07 | 3.16E+00 |  |
| Lipe | 1.42E+03 | 2.97E-09 | 6.76E-07 | -2.09E+00 |  |
| Gm5415 | 2.43E+03 | 3.08E-09 | 6.94E-07 | -2.81E+00 |  |
| Gm24265 | 1.86E+05 | 3.25E-09 | 7.26E-07 | 2.01E+00 |  |
| Gm3404 | 4.18E+02 | 3.31E-09 | 7.32E-07 | -7.74E+00 |  |
| Actl10 | 5.00E+02 | 3.46E-09 | 7.58E-07 | -3.39E+00 |  |
| Gm7358 | 1.54E+03 | 3.75E-09 | 8.13E-07 | -2.15E+00 |  |
| Dctn2 | 1.36E+03 | 3.85E-09 | 8.28E-07 | 1.97E+00 |  |
| Ydjc | 6.21E+02 | 3.95E-09 | 8.41E-07 | -3.39E+00 |  |
| Rnf19a | 4.48E+03 | 4.16E-09 | 8.78E-07 | 1.54E+00 |  |
| H1fnt | 2.94E+03 | 4.34E-09 | 9.07E-07 | -1.97E+00 |  |
| Gm5893 | 5.67E+02 | 5.33E-09 | 1.10E-06 | -3.46E+00 |  |
| Tsga10 | 2.88E+03 | 5.37E-09 | 1.10E-06 | 1.62E+00 |  |
| 4932416K20Rik | 9.21E+02 | 5.39E-09 | 1.10E-06 | -2.91E+00 |  |
| Dnajb8 | 8.03E+02 | 7.21E-09 | 1.46E-06 | -2.22E+00 |  |
| 4930488N24Rik | 9.57E+02 | 7.28E-09 | 1.46E-06 | -2.51E+00 |  |
| Acsl1 | 4.26E+03 | 7.90E-09 | 1.56E-06 | -1.78E+00 |  |
| Clk3 | 2.28E+03 | 7.94E-09 | 1.56E-06 | -1.80E+00 |  |
| Smc3 | 2.37E+03 | 8.70E-09 | 1.70E-06 | -1.82E+00 |  |
| Gm8871 | 3.55E+02 | 9.38E-09 | 1.82E-06 | -8.22E+00 |  |
| Akap12 | 2.24E+04 | 1.01E-08 | 1.94E-06 | -1.74E+00 |  |
| 1700034E13Rik | 8.56E+02 | 1.02E-08 | 1.95E-06 | -2.63E+00 |  |
| Gm21663 | 1.24E+03 | 1.35E-08 | 2.53E-06 | -3.81E+00 |  |
| Akna | 1.14E+03 | 1.35E-08 | 2.53E-06 | -2.14E+00 |  |
| Gm24305 | 2.44E+03 | 1.47E-08 | 2.73E-06 | 2.31E+00 |  |
| Txndc2 | 5.75E+03 | 1.55E-08 | 2.85E-06 | -2.01E+00 |  |
| Gm3883 | 5.40E+02 | 1.59E-08 | 2.92E-06 | -3.06E+00 |  |
| Rbl2 | 1.37E+03 | 1.62E-08 | 2.93E-06 | -2.22E+00 |  |
| Fbp1 | 1.84E+03 | 1.63E-08 | 2.93E-06 | 2.26E+00 |  |
| Tle3 | 1.24E+03 | 1.78E-08 | 3.18E-06 | -2.30E+00 |  |
| Gm4353 | 5.99E+02 | 1.79E-08 | 3.18E-06 | -3.26E+00 |  |
| Tnp2 | 6.81E+03 | 1.81E-08 | 3.19E-06 | -2.33E+00 |  |
| Morf4l1 | 2.01E+03 | 2.22E-08 | 3.88E-06 | 1.84E+00 |  |
| Gm8879 | 5.74E+02 | 2.47E-08 | 4.26E-06 | -3.29E+00 |  |
| Spata3 | 1.08E+03 | 2.47E-08 | 4.26E-06 | -2.43E+00 |  |
| Akap1 | 2.67E+03 | 2.50E-08 | 4.28E-06 | -1.69E+00 |  |
| Zfp292 | 5.51E+02 | 2.73E-08 | 4.64E-06 | -2.50E+00 |  |
| 4921507P07Rik | 1.37E+03 | 2.85E-08 | 4.80E-06 | -2.65E+00 |  |
| Rsrc2 | 1.94E+03 | 3.36E-08 | 5.62E-06 | -1.77E+00 |  |
| Neat1 | 3.02E+03 | 3.85E-08 | 6.39E-06 | 2.31E+00 |  |
| Esrrb | 3.61E+02 | 5.05E-08 | 8.33E-06 | 3.44E+00 |  |
| Fam227a | 1.44E+03 | 5.64E-08 | 9.25E-06 | -1.99E+00 |  |
| Spdye4a | 6.72E+02 | 6.51E-08 | 1.06E-05 | -2.66E+00 |  |
| Gapdhs | 3.78E+03 | 6.61E-08 | 1.07E-05 | -1.97E+00 |  |
| Gm21655 | 6.12E+02 | 6.72E-08 | 1.07E-05 | -4.61E+00 |  |
| 4930572O03Rik | 2.92E+02 | 6.73E-08 | 1.07E-05 | -6.70E+00 |  |
| Pdcl2 | 3.05E+03 | 6.79E-08 | 1.08E-05 | 1.64E+00 |  |
| Gm8897 | 4.98E+02 | 7.21E-08 | 1.13E-05 | -5.02E+00 |  |
| Nsmf | 4.53E+02 | 8.42E-08 | 1.31E-05 | 2.95E+00 |  |
| Gm49895 | 2.51E+02 | 8.53E-08 | 1.32E-05 | -4.65E+00 |  |
| Trip12 | 1.35E+04 | 8.91E-08 | 1.37E-05 | 1.57E+00 |  |
| Reep6 | 2.76E+03 | 9.16E-08 | 1.40E-05 | -2.14E+00 |  |
| Tex29 | 1.38E+03 | 9.55E-08 | 1.45E-05 | -2.04E+00 |  |
| Speer4f1 | 6.30E+02 | 9.78E-08 | 1.48E-05 | -3.61E+00 |  |
| Clk4 | 1.73E+03 | 9.88E-08 | 1.48E-05 | -2.23E+00 |  |
| Cdiptos | 3.05E+03 | 1.36E-07 | 2.03E-05 | -1.62E+00 |  |
| Gm7275 | 8.06E+02 | 1.37E-07 | 2.03E-05 | -2.97E+00 |  |
| Ppme1 | 1.01E+03 | 1.45E-07 | 2.13E-05 | -2.33E+00 |  |
| Sorbs3 | 9.80E+02 | 1.46E-07 | 2.14E-05 | -2.35E+00 |  |
| Tes3-ps | 2.24E+03 | 1.51E-07 | 2.19E-05 | -1.81E+00 |  |
| Qk | 1.66E+03 | 1.52E-07 | 2.19E-05 | 1.72E+00 |  |
| Csnk1g2 | 1.60E+03 | 1.80E-07 | 2.58E-05 | -2.18E+00 |  |
| Pabpc1 | 1.55E+04 | 1.87E-07 | 2.67E-05 | 1.62E+00 |  |
| Ptn | 7.61E+01 | 1.90E-07 | 2.70E-05 | -3.25E+01 |  |
| Rnu3b4 | 1.36E+05 | 1.91E-07 | 2.70E-05 | 1.95E+00 |  |
| Gm9602 | 4.33E+02 | 2.01E-07 | 2.83E-05 | -4.84E+00 |  |
| Nphp1 | 4.59E+03 | 2.10E-07 | 2.93E-05 | 1.58E+00 |  |
| 0610010F05Rik | 9.63E+02 | 2.12E-07 | 2.93E-05 | 1.97E+00 |  |
| BB014433 | 1.09E+03 | 2.13E-07 | 2.93E-05 | -2.01E+00 |  |
| Dusp1 | 2.84E+02 | 2.25E-07 | 3.08E-05 | -4.24E+00 |  |
| Capza1 | 1.11E+03 | 2.38E-07 | 3.25E-05 | 2.08E+00 |  |
| Cenpj | 1.51E+03 | 2.43E-07 | 3.29E-05 | -1.78E+00 |  |
| Fkbp7 | 9.02E+02 | 2.50E-07 | 3.36E-05 | -3.99E+00 |  |
| Ankrd13a | 1.76E+03 | 2.62E-07 | 3.50E-05 | 2.08E+00 |  |
| Rn7s1 | 2.52E+04 | 2.63E-07 | 3.50E-05 | 1.75E+00 |  |
| Ropn1 | 2.46E+03 | 2.98E-07 | 3.94E-05 | -1.86E+00 |  |
| Azin2 | 4.89E+02 | 3.09E-07 | 4.05E-05 | -3.11E+00 |  |
| Speer4e | 7.45E+02 | 3.10E-07 | 4.05E-05 | -3.27E+00 |  |
| Iqcf4 | 1.15E+03 | 3.38E-07 | 4.40E-05 | -2.05E+00 |  |
| Wdr64 | 1.80E+03 | 3.80E-07 | 4.92E-05 | -1.89E+00 |  |
| Zfp608 | 1.77E+03 | 4.05E-07 | 5.21E-05 | -1.86E+00 |  |
| Chrna5 | 1.84E+02 | 4.24E-07 | 5.43E-05 | -5.49E+00 |  |
| Spert | 3.12E+03 | 4.41E-07 | 5.62E-05 | -2.05E+00 |  |
| 1700009J07Rik | 3.70E+02 | 4.47E-07 | 5.67E-05 | -3.17E+00 |  |
| Rbbp6 | 8.61E+03 | 4.67E-07 | 5.89E-05 | -1.54E+00 |  |
| Gm5092 | 4.85E+02 | 4.81E-07 | 6.03E-05 | -2.52E+00 |  |
| Fktn | 2.53E+03 | 4.99E-07 | 6.22E-05 | -1.70E+00 |  |
| Rnu3b3 | 1.37E+05 | 5.06E-07 | 6.27E-05 | 1.92E+00 |  |
| Zdhhc5 | 3.80E+03 | 5.24E-07 | 6.46E-05 | 1.72E+00 |  |
| Gm5861 | 3.69E+02 | 5.30E-07 | 6.50E-05 | -3.47E+00 |  |
| Gm7347 | 8.40E+02 | 6.01E-07 | 7.33E-05 | -4.43E+00 |  |
| Otub2 | 1.25E+03 | 6.59E-07 | 8.00E-05 | -2.03E+00 |  |
| Fam71d | 3.76E+03 | 6.81E-07 | 8.23E-05 | -1.84E+00 |  |
| 1700029H14Rik | 1.98E+03 | 7.14E-07 | 8.57E-05 | -1.88E+00 |  |
| Gm30246 | 7.22E+01 | 7.38E-07 | 8.83E-05 | -2.09E+01 |  |
| Ube2k | 1.41E+03 | 7.44E-07 | 8.85E-05 | -1.81E+00 |  |
| Olfr1372-ps1 | 4.85E+02 | 7.59E-07 | 8.96E-05 | 2.89E+00 |  |
| Usp8 | 3.20E+03 | 7.61E-07 | 8.96E-05 | 1.57E+00 |  |
| Suv39h2 | 6.90E+02 | 7.68E-07 | 8.98E-05 | -2.30E+00 |  |
| Vmn1r28 | 1.91E+02 | 7.70E-07 | 8.98E-05 | -5.81E+00 |  |
| Cep162 | 1.34E+03 | 7.81E-07 | 9.06E-05 | -1.83E+00 |  |
| Acte1 | 3.55E+02 | 8.08E-07 | 9.32E-05 | -3.40E+00 |  |
| Gm21680 | 3.82E+02 | 8.15E-07 | 9.37E-05 | -5.86E+00 |  |
| Cep76 | 6.57E+02 | 9.23E-07 | 1.06E-04 | -2.19E+00 |  |
| Rnu3a | 4.84E+04 | 9.84E-07 | 1.12E-04 | 2.12E+00 |  |
| Sh3rf2 | 7.91E+02 | 9.96E-07 | 1.13E-04 | -2.03E+00 |  |
| Gm8256 | 1.06E+02 | 1.00E-06 | 1.13E-04 | -1.14E+01 |  |
| Oxct2a | 4.74E+02 | 1.05E-06 | 1.17E-04 | -3.27E+00 |  |
| Ptpn20 | 1.96E+03 | 1.13E-06 | 1.26E-04 | 1.61E+00 |  |
| Zfp184 | 9.92E+02 | 1.15E-06 | 1.28E-04 | -2.29E+00 |  |
| Olfr1310 | 1.20E+02 | 1.18E-06 | 1.31E-04 | -1.01E+01 |  |
| Tex35 | 6.87E+02 | 1.23E-06 | 1.36E-04 | -2.38E+00 |  |
| Cdv3 | 2.00E+03 | 1.27E-06 | 1.39E-04 | -1.57E+00 |  |
| Ccdc182 | 7.59E+02 | 1.34E-06 | 1.46E-04 | -2.35E+00 |  |
| Gm5554 | 6.74E+02 | 1.37E-06 | 1.48E-04 | -3.03E+00 |  |
| Btbd1 | 1.44E+03 | 1.40E-06 | 1.51E-04 | 1.77E+00 |  |
| Gm9805 | 1.60E+03 | 1.40E-06 | 1.51E-04 | 1.96E+00 |  |
| Gtsf1l | 2.64E+03 | 1.41E-06 | 1.51E-04 | -1.91E+00 |  |
| Ppp1ccb | 4.77E+03 | 1.43E-06 | 1.52E-04 | 1.74E+00 |  |
| Rimbp3 | 4.48E+03 | 1.46E-06 | 1.54E-04 | 1.59E+00 |  |
| Rn7s2 | 2.60E+04 | 1.48E-06 | 1.56E-04 | 1.73E+00 |  |
| Calr3 | 7.60E+02 | 1.49E-06 | 1.56E-04 | -2.17E+00 |  |
| Pkhd1l1 | 3.45E+02 | 1.52E-06 | 1.58E-04 | -2.64E+00 |  |
| Adam32 | 5.35E+03 | 1.52E-06 | 1.58E-04 | 1.69E+00 |  |
| Gm3867 | 4.27E+01 | 1.53E-06 | 1.58E-04 | -5.37E+01 |  |
| Gm37642 | 4.75E+01 | 1.54E-06 | 1.59E-04 | -5.29E+01 |  |
| Gm24888 | 2.20E+02 | 1.56E-06 | 1.60E-04 | 4.10E+00 |  |
| Rsph6a | 1.03E+03 | 1.57E-06 | 1.61E-04 | 1.91E+00 |  |
| Ccdc166 | 2.89E+02 | 1.67E-06 | 1.70E-04 | -3.03E+00 |  |
| Gm8267 | 2.99E+02 | 1.70E-06 | 1.73E-04 | -4.16E+00 |  |
| Fscn3 | 4.29E+02 | 1.81E-06 | 1.83E-04 | -3.06E+00 |  |
| Triml1 | 1.33E+03 | 1.82E-06 | 1.83E-04 | -1.88E+00 |  |
| Gm23849 | 6.05E+04 | 1.86E-06 | 1.86E-04 | 5.96E+00 |  |
| Gm24950 | 6.11E+04 | 1.90E-06 | 1.89E-04 | 6.00E+00 |  |
| Clk1 | 1.26E+03 | 1.91E-06 | 1.89E-04 | -2.02E+00 |  |
| 4930474N05Rik | 3.88E+03 | 2.00E-06 | 1.98E-04 | -1.66E+00 |  |
| Fbxo11 | 4.53E+02 | 2.04E-06 | 2.00E-04 | 2.51E+00 |  |
| Eef1d | 1.83E+03 | 2.06E-06 | 2.02E-04 | -1.58E+00 |  |
| Atf1 | 8.70E+02 | 2.08E-06 | 2.03E-04 | 1.97E+00 |  |
| Gm6960 | 5.68E+02 | 2.11E-06 | 2.05E-04 | -2.54E+00 |  |
| 4933402J07Rik | 1.67E+03 | 2.11E-06 | 2.05E-04 | -1.58E+00 |  |
| Gm6866 | 1.03E+03 | 2.16E-06 | 2.08E-04 | -2.52E+00 |  |
| Snord67 | 1.35E+03 | 2.24E-06 | 2.15E-04 | 2.60E+00 |  |
| Oscp1 | 1.23E+03 | 2.31E-06 | 2.21E-04 | 1.77E+00 |  |
| Mroh4 | 2.88E+03 | 2.40E-06 | 2.28E-04 | 1.73E+00 |  |
| Rplp0 | 1.13E+03 | 2.41E-06 | 2.29E-04 | 2.21E+00 |  |
| Plpp1 | 4.38E+02 | 2.44E-06 | 2.30E-04 | 2.49E+00 |  |
| Gm6558 | 7.54E+02 | 2.47E-06 | 2.32E-04 | -2.92E+00 |  |
| Tcp1 | 1.07E+04 | 2.49E-06 | 2.34E-04 | 1.64E+00 |  |
| Gm16226 | 6.85E+02 | 2.58E-06 | 2.40E-04 | -2.45E+00 |  |
| Ppm1a | 2.36E+03 | 2.59E-06 | 2.41E-04 | -1.59E+00 |  |
| Lrrc8b | 4.24E+03 | 2.61E-06 | 2.41E-04 | -1.55E+00 |  |
| Cct7 | 3.00E+03 | 2.63E-06 | 2.43E-04 | 1.56E+00 |  |
| Ptprk | 1.27E+02 | 2.71E-06 | 2.49E-04 | -5.42E+00 |  |
| Tmf1 | 3.26E+03 | 2.74E-06 | 2.51E-04 | 1.51E+00 |  |
| Gatad2b | 1.45E+03 | 2.80E-06 | 2.55E-04 | -1.89E+00 |  |
| 1700027A15Rik | 1.09E+03 | 2.81E-06 | 2.55E-04 | -2.45E+00 |  |
| Gm22711 | 2.57E+02 | 2.90E-06 | 2.61E-04 | 7.85E+00 |  |
| Mical2 | 1.75E+03 | 2.90E-06 | 2.61E-04 | -1.65E+00 |  |
| Gm6401 | 1.05E+02 | 3.19E-06 | 2.87E-04 | -8.53E+00 |  |
| Olfr6 | 1.14E+02 | 3.33E-06 | 2.98E-04 | -7.47E+00 |  |
| Gm2916 | 4.39E+02 | 3.42E-06 | 3.04E-04 | 2.85E+00 |  |
| Dgcr6 | 3.58E+02 | 3.52E-06 | 3.12E-04 | 2.93E+00 |  |
| Cct8 | 1.22E+03 | 3.75E-06 | 3.32E-04 | 1.73E+00 |  |
| Spata16 | 1.98E+03 | 4.19E-06 | 3.69E-04 | 1.73E+00 |  |
| Gm12918 | 5.49E+02 | 4.31E-06 | 3.78E-04 | 3.46E+00 |  |
| Gm6853 | 3.20E+03 | 4.35E-06 | 3.81E-04 | -1.77E+00 |  |
| Gm9220 | 1.33E+03 | 4.44E-06 | 3.84E-04 | -2.24E+00 |  |
| Gm7233 | 1.47E+02 | 4.44E-06 | 3.84E-04 | -5.06E+00 |  |
| Gm21083 | 1.80E+03 | 4.44E-06 | 3.84E-04 | -5.72E+00 |  |
| Gm3005 | 1.68E+02 | 4.46E-06 | 3.84E-04 | -8.70E+00 |  |
| Gm25890 | 2.11E+04 | 4.95E-06 | 4.23E-04 | 1.93E+00 |  |
| Ccnl1 | 1.15E+03 | 4.98E-06 | 4.24E-04 | -2.01E+00 |  |
| Selenov | 1.40E+03 | 5.00E-06 | 4.24E-04 | -1.84E+00 |  |
| Ubr5 | 1.14E+04 | 5.01E-06 | 4.24E-04 | 1.52E+00 |  |
| Ntan1 | 2.04E+02 | 5.17E-06 | 4.34E-04 | -7.34E+00 |  |
| Bscl2 | 2.19E+03 | 5.17E-06 | 4.34E-04 | 1.60E+00 |  |
| Lexm | 1.90E+02 | 5.31E-06 | 4.45E-04 | -3.40E+00 |  |
| Gm5591 | 1.46E+03 | 5.33E-06 | 4.45E-04 | -2.67E+00 |  |
| 1700123L14Rik | 3.53E+03 | 5.42E-06 | 4.51E-04 | 1.59E+00 |  |
| Gm26917 | 4.33E+04 | 5.55E-06 | 4.60E-04 | 1.88E+00 |  |
| Gm21190 | 8.97E+02 | 5.60E-06 | 4.62E-04 | -1.07E+01 |  |
| Nfkbiz | 2.04E+02 | 5.63E-06 | 4.62E-04 | -3.34E+00 |  |
| Gm8229 | 1.54E+02 | 5.64E-06 | 4.62E-04 | -6.98E+00 |  |
| Stt3b | 4.15E+03 | 5.66E-06 | 4.62E-04 | 1.66E+00 |  |
| Spn-ps | 6.67E+02 | 5.67E-06 | 4.62E-04 | -2.45E+00 |  |
| Ube2n-ps1 | 1.12E+03 | 5.70E-06 | 4.62E-04 | -1.88E+00 |  |
| Zdhhc11 | 4.21E+02 | 5.93E-06 | 4.79E-04 | -3.03E+00 |  |
| Phf7 | 7.08E+03 | 5.99E-06 | 4.83E-04 | 1.83E+00 |  |
| Pifo | 1.44E+03 | 6.02E-06 | 4.83E-04 | -1.95E+00 |  |
| Ccdc91 | 1.96E+03 | 6.08E-06 | 4.86E-04 | -1.83E+00 |  |
| Zmynd10 | 1.79E+03 | 6.30E-06 | 5.02E-04 | 1.68E+00 |  |
| Pdzd8 | 2.39E+03 | 6.34E-06 | 5.03E-04 | -1.84E+00 |  |
| Nudt16l2 | 2.20E+02 | 6.40E-06 | 5.05E-04 | -3.13E+00 |  |
| Jpt1 | 6.56E+02 | 6.43E-06 | 5.05E-04 | 2.26E+00 |  |
| Dydc1 | 7.95E+02 | 6.50E-06 | 5.09E-04 | -2.11E+00 |  |
| Atxn7l3b | 4.42E+03 | 6.67E-06 | 5.21E-04 | 1.52E+00 |  |
| Scara5 | 4.59E+02 | 6.70E-06 | 5.22E-04 | 2.57E+00 |  |
| Rasa3 | 5.11E+02 | 6.74E-06 | 5.23E-04 | -2.20E+00 |  |
| Pten | 2.26E+03 | 6.92E-06 | 5.34E-04 | 1.65E+00 |  |
| Eef1a1 | 4.45E+03 | 6.93E-06 | 5.34E-04 | 1.69E+00 |  |
| Vps37a | 1.38E+03 | 7.03E-06 | 5.38E-04 | 1.73E+00 |  |
| Cabyr | 3.21E+03 | 7.03E-06 | 5.38E-04 | -1.58E+00 |  |
| Nipsnap3a | 7.52E+02 | 7.55E-06 | 5.76E-04 | -2.40E+00 |  |
| 4933402N22Rik | 6.00E+02 | 7.61E-06 | 5.79E-04 | -3.19E+00 |  |
| Ddx25 | 1.09E+03 | 7.72E-06 | 5.86E-04 | -9.75E+00 |  |
| Nt5c1b | 1.74E+03 | 7.79E-06 | 5.89E-04 | -1.69E+00 |  |
| Snord17 | 7.17E+03 | 7.94E-06 | 5.95E-04 | 2.91E+00 |  |
| 4930444G20Rik | 1.11E+03 | 7.94E-06 | 5.95E-04 | -2.50E+00 |  |
| Gm7049 | 4.66E+02 | 7.94E-06 | 5.95E-04 | 2.49E+00 |  |
| Gm24924 | 1.38E+02 | 8.13E-06 | 6.06E-04 | 1.29E+01 |  |
| Edem3 | 8.55E+02 | 8.36E-06 | 6.22E-04 | -2.26E+00 |  |
| Prr30 | 3.19E+03 | 8.54E-06 | 6.33E-04 | -1.60E+00 |  |
| Papolb | 6.24E+03 | 8.69E-06 | 6.42E-04 | 1.53E+00 |  |
| 4930429C20Rik | 2.85E+02 | 8.75E-06 | 6.44E-04 | -2.93E+00 |  |
| Naa60 | 5.11E+02 | 9.01E-06 | 6.62E-04 | -2.30E+00 |  |
| 1700061G19Rik | 1.40E+03 | 9.21E-06 | 6.73E-04 | -1.67E+00 |  |
| Rpl10l | 7.91E+02 | 9.23E-06 | 6.73E-04 | 1.99E+00 |  |
| Rpl13-ps6 | 1.04E+02 | 9.30E-06 | 6.77E-04 | -3.28E+01 |  |
| Gm1821 | 1.65E+03 | 9.35E-06 | 6.79E-04 | 1.82E+00 |  |
| Ttll10 | 2.38E+03 | 9.41E-06 | 6.80E-04 | -1.58E+00 |  |
| Fam186a | 5.03E+03 | 9.51E-06 | 6.86E-04 | -1.51E+00 |  |
| Gm48051 | 1.47E+04 | 9.95E-06 | 7.15E-04 | -3.29E+00 |  |
| Orc6 | 1.85E+02 | 1.01E-05 | 7.22E-04 | -3.51E+00 |  |
| Rpph1 | 6.32E+03 | 1.03E-05 | 7.36E-04 | 1.77E+00 |  |
| Gm22614 | 2.04E+04 | 1.05E-05 | 7.47E-04 | 1.97E+00 |  |
| Dnajb1 | 1.49E+03 | 1.06E-05 | 7.48E-04 | 1.81E+00 |  |
| Lelp1 | 6.11E+02 | 1.06E-05 | 7.48E-04 | -1.98E+00 |  |
| Gm12669 | 1.56E+03 | 1.06E-05 | 7.48E-04 | 1.63E+00 |  |
| Usp34 | 6.31E+03 | 1.09E-05 | 7.65E-04 | 1.62E+00 |  |
| Gm3402 | 5.31E+02 | 1.10E-05 | 7.72E-04 | -5.40E+00 |  |
| Actg2 | 6.99E+02 | 1.11E-05 | 7.74E-04 | -2.18E+00 |  |
| Gm21093 | 1.03E+03 | 1.13E-05 | 7.90E-04 | -2.56E+00 |  |
| Gm8232 | 1.27E+02 | 1.17E-05 | 8.17E-04 | -6.27E+00 |  |
| Gm44106 | 5.96E+01 | 1.19E-05 | 8.23E-04 | -2.39E+01 |  |
| Macf1 | 2.00E+03 | 1.22E-05 | 8.41E-04 | 1.56E+00 |  |
| Hk1 | 5.37E+03 | 1.22E-05 | 8.41E-04 | -1.62E+00 |  |
| Gar1 | 2.18E+02 | 1.22E-05 | 8.41E-04 | -3.15E+00 |  |
| Arhgap5 | 4.34E+03 | 1.27E-05 | 8.66E-04 | 1.63E+00 |  |
| Gm8024 | 6.62E+01 | 1.27E-05 | 8.66E-04 | -2.87E+01 |  |
| Snord118 | 1.05E+04 | 1.27E-05 | 8.66E-04 | 4.61E+00 |  |
| n-R5-8s1 | 2.67E+04 | 1.28E-05 | 8.72E-04 | 4.06E+00 |  |
| Gm22486 | 6.31E+02 | 1.31E-05 | 8.87E-04 | 4.49E+00 |  |
| Spata20 | 4.00E+03 | 1.32E-05 | 8.91E-04 | -1.56E+00 |  |
| Cmtm2a | 2.30E+03 | 1.32E-05 | 8.92E-04 | 1.72E+00 |  |
| Ier5 | 3.91E+02 | 1.34E-05 | 8.99E-04 | -2.60E+00 |  |
| Klf2 | 3.97E+02 | 1.35E-05 | 9.02E-04 | -3.13E+00 |  |
| Tbpl1 | 1.34E+03 | 1.35E-05 | 9.02E-04 | 1.80E+00 |  |
| Lrrcc1 | 3.16E+03 | 1.36E-05 | 9.05E-04 | 1.64E+00 |  |
| Txnrd3 | 3.36E+03 | 1.37E-05 | 9.10E-04 | 1.61E+00 |  |
| Zfp28 | 3.73E+02 | 1.40E-05 | 9.25E-04 | -2.45E+00 |  |
| Pcbp2 | 2.56E+03 | 1.41E-05 | 9.28E-04 | 1.85E+00 |  |
| Vmn1r80 | 2.24E+02 | 1.44E-05 | 9.45E-04 | -4.94E+00 |  |
| Strn4 | 1.03E+03 | 1.44E-05 | 9.45E-04 | 1.92E+00 |  |
| Gm23143 | 1.78E+03 | 1.49E-05 | 9.70E-04 | 1.15E+01 |  |
| 2610301B20Rik | 1.09E+03 | 1.49E-05 | 9.72E-04 | -1.67E+00 |  |
| Trp53tg5 | 4.60E+02 | 1.52E-05 | 9.84E-04 | -2.43E+00 |  |
| Phc2 | 3.71E+02 | 1.56E-05 | 1.01E-03 | 2.68E+00 |  |
| Cep112os1 | 9.31E+02 | 1.58E-05 | 1.02E-03 | -1.90E+00 |  |
| Snora78 | 5.39E+02 | 1.58E-05 | 1.02E-03 | 4.18E+00 |  |
| Rd3 | 8.78E+02 | 1.60E-05 | 1.03E-03 | -2.22E+00 |  |
| Fam243 | 1.68E+03 | 1.61E-05 | 1.03E-03 | -1.66E+00 |  |
| Rmnd1 | 6.15E+02 | 1.65E-05 | 1.06E-03 | -2.28E+00 |  |
| Samd4 | 1.78E+03 | 1.73E-05 | 1.10E-03 | -1.59E+00 |  |
| Tmsb10 | 1.55E+03 | 1.74E-05 | 1.10E-03 | 1.93E+00 |  |
| Gm2666 | 5.13E+01 | 1.76E-05 | 1.11E-03 | -8.75E+01 |  |
| Cdkn2c | 6.72E+02 | 1.82E-05 | 1.14E-03 | 2.09E+00 |  |
| Ldhal6b | 3.85E+03 | 1.82E-05 | 1.14E-03 | 1.58E+00 |  |
| Vmn2r-ps94 | 3.06E+01 | 1.82E-05 | 1.14E-03 | -5.79E+01 |  |
| 4930500M09Rik | 4.58E+01 | 1.85E-05 | 1.16E-03 | -2.89E+01 |  |
| Blm | 6.77E+02 | 1.89E-05 | 1.18E-03 | 2.00E+00 |  |
| Wdpcp | 5.29E+02 | 1.93E-05 | 1.20E-03 | 2.29E+00 |  |
| Gm6455 | 5.75E+02 | 1.94E-05 | 1.20E-03 | -3.07E+00 |  |
| Slc1a7 | 1.11E+02 | 1.97E-05 | 1.22E-03 | -4.68E+00 |  |
| Wbp2nl | 4.97E+02 | 2.00E-05 | 1.23E-03 | -2.21E+00 |  |
| Gm25107 | 1.09E+03 | 2.00E-05 | 1.23E-03 | 2.81E+00 |  |
| Gm25813 | 2.47E+03 | 2.02E-05 | 1.24E-03 | 1.54E+01 |  |
| Hyal6 | 1.70E+03 | 2.05E-05 | 1.25E-03 | 1.61E+00 |  |
| Vmn2r104 | 2.25E+02 | 2.06E-05 | 1.25E-03 | -5.87E+00 |  |
| Gm23472 | 2.63E+04 | 2.08E-05 | 1.26E-03 | 3.28E+00 |  |
| Prkcq | 7.74E+02 | 2.11E-05 | 1.27E-03 | -1.91E+00 |  |
| Lca5 | 1.28E+03 | 2.14E-05 | 1.29E-03 | -1.89E+00 |  |
| Gm23444 | 2.58E+04 | 2.17E-05 | 1.30E-03 | 3.26E+00 |  |
| Gm6811 | 9.08E+02 | 2.17E-05 | 1.30E-03 | -2.14E+00 |  |
| Rnls | 5.09E+02 | 2.20E-05 | 1.31E-03 | 2.41E+00 |  |
| Gm6760 | 6.44E+02 | 2.21E-05 | 1.31E-03 | -2.52E+00 |  |
| Leo1 | 1.04E+03 | 2.21E-05 | 1.31E-03 | -1.73E+00 |  |
| Gm6818 | 8.37E+02 | 2.22E-05 | 1.31E-03 | -2.08E+00 |  |
| Sacs | 1.97E+03 | 2.23E-05 | 1.31E-03 | -1.57E+00 |  |
| Gdpd1 | 1.23E+03 | 2.28E-05 | 1.34E-03 | -1.75E+00 |  |
| Snora57 | 9.35E+02 | 2.29E-05 | 1.35E-03 | 3.10E+00 |  |
| Marchf11 | 2.31E+03 | 2.31E-05 | 1.35E-03 | 1.70E+00 |  |
| Olfr1263 | 9.90E+01 | 2.32E-05 | 1.35E-03 | -7.18E+00 |  |
| Fkbp4 | 2.01E+03 | 2.34E-05 | 1.36E-03 | 1.75E+00 |  |
| Gm6408 | 3.57E+02 | 2.36E-05 | 1.37E-03 | -5.25E+00 |  |
| Rhot1 | 7.14E+02 | 2.37E-05 | 1.37E-03 | 1.87E+00 |  |
| Gm6309 | 5.57E+02 | 2.37E-05 | 1.37E-03 | -3.12E+00 |  |
| Gm35386 | 4.64E+02 | 2.44E-05 | 1.40E-03 | -2.27E+00 |  |
| Slamf6 | 1.28E+02 | 2.53E-05 | 1.45E-03 | -9.77E+00 |  |
| Trim45 | 3.70E+02 | 2.55E-05 | 1.46E-03 | -2.28E+00 |  |
| Xpo6 | 2.33E+03 | 2.57E-05 | 1.47E-03 | 1.54E+00 |  |
| Lemd1 | 3.89E+02 | 2.59E-05 | 1.48E-03 | -2.29E+00 |  |
| Gm7954 | 8.90E+01 | 2.62E-05 | 1.49E-03 | -1.62E+01 |  |
| Prss51 | 2.13E+02 | 2.63E-05 | 1.49E-03 | -3.08E+00 |  |
| A530065N20Rik | 6.58E+01 | 2.66E-05 | 1.51E-03 | -9.22E+00 |  |
| Nnmt | 6.66E+01 | 2.68E-05 | 1.51E-03 | -1.45E+01 |  |
| 4930503B20Rik | 1.35E+03 | 2.69E-05 | 1.51E-03 | -1.87E+00 |  |
| Cep112 | 1.87E+03 | 2.73E-05 | 1.53E-03 | -1.54E+00 |  |
| Flrt2 | 1.60E+02 | 2.74E-05 | 1.53E-03 | -4.96E+00 |  |
| Cdrt4 | 6.16E+02 | 2.75E-05 | 1.54E-03 | -2.10E+00 |  |
| Rnf133 | 6.38E+02 | 2.76E-05 | 1.54E-03 | -2.14E+00 |  |
| Tmbim7 | 1.07E+03 | 2.77E-05 | 1.54E-03 | 1.92E+00 |  |
| Arsa | 1.27E+03 | 2.85E-05 | 1.58E-03 | 1.72E+00 |  |
| Dcaf1 | 3.14E+03 | 2.91E-05 | 1.61E-03 | -1.61E+00 |  |
| Dnah8 | 3.90E+04 | 2.96E-05 | 1.64E-03 | 1.52E+00 |  |
| Dync1h1 | 7.98E+03 | 3.01E-05 | 1.66E-03 | 1.51E+00 |  |
| Cdkl4 | 1.05E+03 | 3.03E-05 | 1.67E-03 | -1.98E+00 |  |
| Pgp | 1.82E+03 | 3.12E-05 | 1.71E-03 | 1.84E+00 |  |
| 4930563J15Rik | 7.95E+02 | 3.17E-05 | 1.73E-03 | -1.82E+00 |  |
| Gm7356 | 1.85E+03 | 3.23E-05 | 1.76E-03 | -1.57E+00 |  |
| 1700101O22Rik | 4.32E+02 | 3.24E-05 | 1.76E-03 | -3.25E+00 |  |
| Lingo2 | 1.20E+02 | 3.28E-05 | 1.78E-03 | -9.42E+00 |  |
| Zfp541 | 2.27E+03 | 3.31E-05 | 1.79E-03 | 1.59E+00 |  |
| Aldh1a2 | 5.68E+02 | 3.32E-05 | 1.79E-03 | 1.91E+00 |  |
| Gm6370 | 4.09E+02 | 3.32E-05 | 1.79E-03 | -3.73E+00 |  |
| 2810025M15Rik | 3.56E+02 | 3.47E-05 | 1.86E-03 | 2.65E+00 |  |
| Gm43820 | 5.58E+01 | 3.54E-05 | 1.89E-03 | -3.52E+01 |  |
| Nufip2 | 1.61E+03 | 3.55E-05 | 1.89E-03 | 1.77E+00 |  |
| Pigg | 5.29E+02 | 3.59E-05 | 1.91E-03 | -2.27E+00 |  |
| Gm36445 | 1.71E+03 | 3.67E-05 | 1.95E-03 | -2.04E+00 |  |
| Prkar2a | 9.87E+02 | 3.82E-05 | 2.03E-03 | -2.01E+00 |  |
| Gm22363 | 1.32E+02 | 3.83E-05 | 2.03E-03 | 1.84E+01 |  |
| Gm5565 | 2.62E+02 | 3.84E-05 | 2.03E-03 | -3.36E+00 |  |
| G2e3 | 1.53E+03 | 3.97E-05 | 2.09E-03 | -1.74E+00 |  |
| Gm30531 | 4.81E+01 | 4.10E-05 | 2.15E-03 | -1.50E+01 |  |
| Vmn1r68 | 1.49E+02 | 4.10E-05 | 2.15E-03 | -5.05E+00 |  |
| Ncoa4 | 1.29E+03 | 4.12E-05 | 2.15E-03 | 1.66E+00 |  |
| Spats2 | 7.04E+02 | 4.13E-05 | 2.15E-03 | 2.09E+00 |  |
| Vdac2 | 2.35E+03 | 4.13E-05 | 2.15E-03 | 1.58E+00 |  |
| Spata48 | 8.97E+02 | 4.28E-05 | 2.22E-03 | -2.02E+00 |  |
| Zfp58 | 7.79E+02 | 4.44E-05 | 2.30E-03 | -2.12E+00 |  |
| Coq6 | 2.72E+02 | 4.50E-05 | 2.33E-03 | -2.48E+00 |  |
| Cmtm2b | 1.69E+03 | 4.55E-05 | 2.34E-03 | 1.68E+00 |  |
| Gm25679 | 1.04E+02 | 4.55E-05 | 2.34E-03 | 9.49E+00 |  |
| Ppwd1 | 7.42E+02 | 4.55E-05 | 2.34E-03 | -1.99E+00 |  |
| Cdc14a | 1.37E+03 | 4.57E-05 | 2.34E-03 | -1.56E+00 |  |
| Gm23301 | 2.12E+02 | 4.62E-05 | 2.36E-03 | 1.04E+01 |  |
| Antxrl | 1.60E+03 | 4.63E-05 | 2.36E-03 | -1.71E+00 |  |
| Actg1 | 3.93E+03 | 4.80E-05 | 2.44E-03 | 1.81E+00 |  |
| Slc25a41 | 3.03E+02 | 4.88E-05 | 2.48E-03 | -2.98E+00 |  |
| Eppk1 | 1.45E+03 | 4.98E-05 | 2.53E-03 | 1.61E+00 |  |
| Nup35 | 5.48E+02 | 5.00E-05 | 2.53E-03 | -2.22E+00 |  |
| Gm10471 | 4.53E+02 | 5.02E-05 | 2.54E-03 | -3.03E+00 |  |
| Tuba8 | 1.34E+03 | 5.04E-05 | 2.54E-03 | -1.76E+00 |  |
| Alx4 | 6.97E+01 | 5.13E-05 | 2.58E-03 | -1.52E+01 |  |
| Gm8180 | 1.46E+02 | 5.36E-05 | 2.68E-03 | -4.47E+00 |  |
| Rimklb | 2.77E+03 | 5.56E-05 | 2.78E-03 | 1.67E+00 |  |
| Gm3252 | 1.62E+02 | 5.60E-05 | 2.79E-03 | -3.88E+00 |  |
| Naa80 | 6.40E+02 | 5.87E-05 | 2.92E-03 | -2.08E+00 |  |
| 1700028J19Rik | 1.46E+03 | 5.88E-05 | 2.92E-03 | 1.93E+00 |  |
| Slc2a3 | 1.19E+04 | 5.91E-05 | 2.92E-03 | 1.55E+00 |  |
| Gm24497 | 3.61E+04 | 5.93E-05 | 2.92E-03 | 3.05E+00 |  |
| Gm22317 | 9.80E+01 | 5.94E-05 | 2.92E-03 | 8.86E+00 |  |
| Ly6g6c | 2.85E+02 | 5.94E-05 | 2.92E-03 | -2.94E+00 |  |
| 1700001F09Rik | 1.29E+03 | 6.06E-05 | 2.98E-03 | -1.89E+00 |  |
| Bbs5 | 9.17E+02 | 6.10E-05 | 2.99E-03 | -2.08E+00 |  |
| Thsd7a | 2.42E+02 | 6.16E-05 | 3.01E-03 | -2.77E+00 |  |
| Gm17081 | 5.56E+02 | 6.18E-05 | 3.02E-03 | -2.48E+00 |  |
| Pabpc6 | 5.82E+03 | 6.21E-05 | 3.03E-03 | 1.59E+00 |  |
| Cds1 | 9.76E+02 | 6.23E-05 | 3.03E-03 | 1.71E+00 |  |
| Olfr1178 | 9.48E+01 | 6.42E-05 | 3.11E-03 | -8.56E+00 |  |
| Crbn | 6.32E+02 | 6.47E-05 | 3.13E-03 | 1.97E+00 |  |
| Gm24826 | 4.94E+01 | 6.51E-05 | 3.14E-03 | 3.40E+01 |  |
| Rab30 | 2.85E+02 | 6.52E-05 | 3.15E-03 | -4.69E+00 |  |
| Gm4787 | 1.95E+03 | 6.56E-05 | 3.15E-03 | -1.57E+00 |  |
| Vmn2r113 | 4.18E+02 | 6.58E-05 | 3.15E-03 | -3.72E+00 |  |
| Gm24044 | 4.04E+03 | 6.66E-05 | 3.18E-03 | 1.73E+00 |  |
| Gm26165 | 6.50E+02 | 6.86E-05 | 3.27E-03 | 3.63E+00 |  |
| Srgap2 | 7.05E+02 | 6.88E-05 | 3.27E-03 | 1.90E+00 |  |
| Olfr1183 | 1.37E+02 | 6.97E-05 | 3.30E-03 | -5.11E+00 |  |
| AA986860 | 1.55E+02 | 6.98E-05 | 3.30E-03 | -5.01E+00 |  |
| Ero1lb | 2.04E+03 | 7.08E-05 | 3.34E-03 | 1.56E+00 |  |
| Aplp2 | 8.42E+02 | 7.17E-05 | 3.38E-03 | 1.81E+00 |  |
| Jarid2 | 1.32E+03 | 7.19E-05 | 3.38E-03 | -1.78E+00 |  |
| Zfp72 | 1.47E+03 | 7.43E-05 | 3.49E-03 | -2.22E+00 |  |
| Gm26244 | 9.61E+01 | 7.66E-05 | 3.58E-03 | 8.68E+00 |  |
| Gm48521 | 1.23E+02 | 7.67E-05 | 3.58E-03 | -3.88E+00 |  |
| Cnga1 | 1.11E+02 | 7.68E-05 | 3.58E-03 | -6.21E+00 |  |
| Gm8356 | 2.83E+02 | 7.87E-05 | 3.67E-03 | -3.05E+00 |  |
| 1700015G11Rik | 5.61E+02 | 7.89E-05 | 3.67E-03 | -2.30E+00 |  |
| 5330437M03Rik | 5.33E+01 | 8.06E-05 | 3.74E-03 | -1.11E+01 |  |
| Gm8906 | 4.99E+02 | 8.08E-05 | 3.74E-03 | -2.62E+00 |  |
| Snora3 | 9.72E+02 | 8.10E-05 | 3.74E-03 | 5.26E+00 |  |
| Dnajc5b | 1.18E+03 | 8.16E-05 | 3.76E-03 | -1.85E+00 |  |
| Fam45a | 9.99E+02 | 8.17E-05 | 3.76E-03 | 1.63E+00 |  |
| Erich3 | 2.24E+03 | 8.19E-05 | 3.76E-03 | -1.60E+00 |  |
| 2810029C07Rik | 5.23E+02 | 8.20E-05 | 3.76E-03 | -2.12E+00 |  |
| Paip2 | 1.64E+03 | 8.25E-05 | 3.77E-03 | -1.54E+00 |  |
| Hook2 | 6.35E+02 | 8.33E-05 | 3.80E-03 | 1.85E+00 |  |
| Gm24119 | 6.55E+02 | 8.33E-05 | 3.80E-03 | 3.67E+00 |  |
| Fam98a | 1.33E+03 | 8.35E-05 | 3.80E-03 | 1.73E+00 |  |
| Selenow | 7.97E+02 | 8.44E-05 | 3.83E-03 | 1.83E+00 |  |
| Camsap1 | 2.91E+03 | 8.51E-05 | 3.85E-03 | 1.50E+00 |  |
| Ace | 2.53E+03 | 8.52E-05 | 3.85E-03 | -1.57E+00 |  |
| Oxct2b | 3.36E+02 | 8.56E-05 | 3.86E-03 | -2.24E+00 |  |
| Snord14c | 1.78E+02 | 8.61E-05 | 3.88E-03 | 5.40E+00 |  |
| Ssxb3 | 7.93E+01 | 8.68E-05 | 3.91E-03 | -8.06E+00 |  |
| Gk2 | 4.20E+03 | 8.75E-05 | 3.93E-03 | 1.57E+00 |  |
| Isg20l2 | 9.63E+02 | 8.87E-05 | 3.97E-03 | -1.79E+00 |  |
| C130026I21Rik | 1.47E+02 | 8.98E-05 | 4.01E-03 | -4.35E+00 |  |
| Gm48228 | 1.67E+02 | 9.10E-05 | 4.06E-03 | -4.40E+00 |  |
| BC048671 | 7.15E+02 | 9.29E-05 | 4.13E-03 | -2.07E+00 |  |
| Cops4 | 9.36E+02 | 9.34E-05 | 4.14E-03 | 1.70E+00 |  |
| Tprgl | 6.63E+02 | 9.40E-05 | 4.16E-03 | 2.03E+00 |  |
| Gm1979 | 5.03E+02 | 9.43E-05 | 4.17E-03 | -3.55E+00 |  |
| Gm2869 | 4.36E+01 | 9.48E-05 | 4.18E-03 | -2.23E+01 |  |
| Slfnl1 | 1.05E+03 | 9.49E-05 | 4.18E-03 | -1.72E+00 |  |
| Cdc37l1 | 1.45E+03 | 9.63E-05 | 4.22E-03 | 1.53E+00 |  |
| Ybx1 | 3.56E+03 | 9.67E-05 | 4.24E-03 | 1.55E+00 |  |
| Gm42680 | 4.88E+01 | 9.71E-05 | 4.25E-03 | -3.14E+01 |  |
| Tram1 | 1.25E+03 | 9.80E-05 | 4.27E-03 | 1.89E+00 |  |
| Vamp3 | 6.90E+02 | 9.83E-05 | 4.27E-03 | 1.83E+00 |  |
| Zfp110 | 1.90E+03 | 9.83E-05 | 4.27E-03 | 1.80E+00 |  |
| Orc1 | 2.02E+02 | 9.84E-05 | 4.27E-03 | -3.03E+00 |  |
| Wdr35 | 2.52E+03 | 9.89E-05 | 4.28E-03 | 1.55E+00 |  |
| Gm49027 | 4.98E+01 | 1.00E-04 | 4.32E-03 | 2.23E+01 |  |
| Gm24407 | 4.30E+04 | 1.01E-04 | 4.36E-03 | 3.13E+00 |  |
| Ccdc70 | 1.12E+03 | 1.05E-04 | 4.52E-03 | -1.57E+00 |  |
| Vmn2r-ps61 | 4.20E+01 | 1.07E-04 | 4.58E-03 | -1.91E+01 |  |
| Kcnh8 | 1.49E+02 | 1.07E-04 | 4.58E-03 | 3.47E+00 |  |
| Gm42986 | 6.40E+02 | 1.07E-04 | 4.58E-03 | 2.12E+00 |  |
| 1700011L22Rik | 7.06E+02 | 1.09E-04 | 4.64E-03 | -2.06E+00 |  |
| Tasp1 | 1.04E+03 | 1.11E-04 | 4.72E-03 | -1.71E+00 |  |
| Bcl2l14 | 1.53E+03 | 1.12E-04 | 4.75E-03 | -1.70E+00 |  |
| Gm43280 | 1.69E+02 | 1.12E-04 | 4.76E-03 | 4.23E+00 |  |
| 1700096J18Rik | 6.61E+01 | 1.13E-04 | 4.77E-03 | -6.74E+00 |  |
| Ptpa | 1.03E+03 | 1.13E-04 | 4.78E-03 | 1.71E+00 |  |
| Plekhg2 | 3.08E+02 | 1.14E-04 | 4.81E-03 | 2.38E+00 |  |
| Gm3248 | 1.35E+02 | 1.14E-04 | 4.81E-03 | -4.01E+00 |  |
| Spata31d1d | 1.56E+03 | 1.16E-04 | 4.86E-03 | -1.64E+00 |  |
| Olfr904 | 8.80E+01 | 1.20E-04 | 5.02E-03 | -7.65E+00 |  |
| Terf2ip | 2.35E+02 | 1.20E-04 | 5.03E-03 | -2.96E+00 |  |
| Dpy19l2 | 2.00E+03 | 1.21E-04 | 5.04E-03 | 1.59E+00 |  |
| Csnk1a1 | 1.95E+03 | 1.22E-04 | 5.07E-03 | 1.60E+00 |  |
| Gm26801 | 5.71E+01 | 1.22E-04 | 5.08E-03 | -1.45E+01 |  |
| Gm12378 | 6.67E+02 | 1.23E-04 | 5.09E-03 | -2.07E+00 |  |
| Snora81 | 4.63E+03 | 1.23E-04 | 5.09E-03 | 3.07E+00 |  |
| Hsd3b3 | 1.09E+02 | 1.23E-04 | 5.09E-03 | -9.73E+00 |  |
| Smok3b | 4.62E+02 | 1.24E-04 | 5.11E-03 | -3.65E+00 |  |
| Fam50b | 8.53E+02 | 1.24E-04 | 5.11E-03 | 1.77E+00 |  |
| 4933428P19Rik | 2.28E+02 | 1.24E-04 | 5.11E-03 | -4.03E+00 |  |
| Pmfbp1 | 2.98E+03 | 1.25E-04 | 5.12E-03 | -1.68E+00 |  |
| Ubl4b | 7.37E+02 | 1.25E-04 | 5.12E-03 | -1.87E+00 |  |
| Nudt4 | 5.07E+03 | 1.25E-04 | 5.13E-03 | 1.66E+00 |  |
| Prrg1 | 5.12E+01 | 1.26E-04 | 5.14E-03 | -1.09E+01 |  |
| 1700009N14Rik | 1.77E+03 | 1.26E-04 | 5.14E-03 | -1.73E+00 |  |
| Smurf2 | 8.48E+02 | 1.27E-04 | 5.16E-03 | 1.93E+00 |  |
| Gm4181 | 3.35E+02 | 1.27E-04 | 5.17E-03 | -4.58E+00 |  |
| Gm21103 | 5.64E+02 | 1.28E-04 | 5.20E-03 | -2.05E+00 |  |
| Olfr1256 | 1.01E+02 | 1.30E-04 | 5.26E-03 | -7.62E+00 |  |
| Camk1d | 2.87E+02 | 1.30E-04 | 5.27E-03 | -2.43E+00 |  |
| Fbxo34 | 8.40E+02 | 1.31E-04 | 5.30E-03 | -1.82E+00 |  |
| Olfr103 | 6.94E+01 | 1.32E-04 | 5.32E-03 | -1.82E+01 |  |
| Slc26a8 | 1.49E+03 | 1.33E-04 | 5.33E-03 | 1.65E+00 |  |
| Gm23262 | 1.65E+03 | 1.33E-04 | 5.33E-03 | 4.53E+00 |  |
| Stk25 | 1.14E+03 | 1.33E-04 | 5.33E-03 | 1.87E+00 |  |
| Gm37985 | 8.41E+02 | 1.34E-04 | 5.36E-03 | -3.15E+00 |  |
| Gm23971 | 4.22E+04 | 1.36E-04 | 5.41E-03 | 6.56E+00 |  |
| Slc39a6 | 1.07E+03 | 1.39E-04 | 5.55E-03 | 1.67E+00 |  |
| Phf10 | 1.40E+03 | 1.40E-04 | 5.57E-03 | 1.63E+00 |  |
| 4930588J15Rik | 3.63E+02 | 1.40E-04 | 5.57E-03 | -2.19E+00 |  |
| Gm7697 | 7.96E+02 | 1.41E-04 | 5.60E-03 | -1.92E+00 |  |
| Yod1 | 1.39E+03 | 1.42E-04 | 5.60E-03 | -1.60E+00 |  |
| Nedd9 | 1.85E+02 | 1.42E-04 | 5.60E-03 | -2.71E+00 |  |
| Fam166a | 1.03E+03 | 1.42E-04 | 5.61E-03 | -1.99E+00 |  |
| Slc35a5 | 8.49E+02 | 1.43E-04 | 5.63E-03 | -1.81E+00 |  |
| Sema3c | 6.55E+01 | 1.45E-04 | 5.71E-03 | -7.95E+00 |  |
| Gm24270 | 2.72E+02 | 1.47E-04 | 5.77E-03 | 7.89E+00 |  |
| Gm3194 | 1.46E+02 | 1.49E-04 | 5.84E-03 | -3.44E+00 |  |
| Ksr2 | 6.63E+02 | 1.50E-04 | 5.85E-03 | -1.89E+00 |  |
| Efcab12 | 9.09E+02 | 1.51E-04 | 5.89E-03 | -1.73E+00 |  |
| Xirp2 | 1.46E+02 | 1.51E-04 | 5.89E-03 | -5.96E+00 |  |
| 4930544G11Rik | 1.69E+03 | 1.52E-04 | 5.91E-03 | 1.68E+00 |  |
| Dnajb7 | 1.27E+03 | 1.53E-04 | 5.94E-03 | -1.70E+00 |  |
| Dach2 | 7.38E+01 | 1.54E-04 | 5.97E-03 | -8.43E+00 |  |
| Satl1 | 3.69E+02 | 1.55E-04 | 5.98E-03 | -2.37E+00 |  |
| Spata32 | 3.00E+02 | 1.57E-04 | 6.08E-03 | -2.29E+00 |  |
| Slc26a11 | 1.25E+02 | 1.62E-04 | 6.24E-03 | 6.89E+00 |  |
| Vmn1r60 | 1.07E+02 | 1.63E-04 | 6.27E-03 | -4.26E+00 |  |
| C130073E24Rik | 2.51E+02 | 1.64E-04 | 6.27E-03 | -3.41E+00 |  |
| Arhgap33 | 1.06E+03 | 1.64E-04 | 6.28E-03 | 1.64E+00 |  |
| Kif20a | 9.44E+02 | 1.65E-04 | 6.29E-03 | 1.89E+00 |  |
| Prm3 | 1.31E+03 | 1.70E-04 | 6.50E-03 | -1.78E+00 |  |
| Klhl25 | 6.94E+02 | 1.73E-04 | 6.59E-03 | -1.77E+00 |  |
| Sem1 | 1.53E+03 | 1.73E-04 | 6.59E-03 | -1.51E+00 |  |
| Gm14121 | 1.05E+03 | 1.74E-04 | 6.61E-03 | 1.75E+00 |  |
| Speer4a | 4.27E+02 | 1.75E-04 | 6.62E-03 | -2.32E+00 |  |
| Ranbp17 | 1.44E+03 | 1.75E-04 | 6.62E-03 | 1.67E+00 |  |
| Olfr116 | 5.70E+01 | 1.75E-04 | 6.62E-03 | -1.41E+01 |  |
| Cpt1b | 1.58E+03 | 1.76E-04 | 6.64E-03 | 1.64E+00 |  |
| Glrx2 | 1.30E+03 | 1.77E-04 | 6.67E-03 | -1.55E+00 |  |
| Clmp | 1.01E+02 | 1.78E-04 | 6.68E-03 | -5.08E+00 |  |
| Dpep2nb | 1.41E+02 | 1.79E-04 | 6.70E-03 | -5.11E+00 |  |
| Wdr48 | 3.99E+03 | 1.79E-04 | 6.70E-03 | 1.53E+00 |  |
| Gm43263 | 2.08E+02 | 1.82E-04 | 6.81E-03 | -2.97E+00 |  |
| Gm25099 | 4.77E+03 | 1.83E-04 | 6.81E-03 | 3.35E+01 |  |
| 1700019N19Rik | 1.19E+03 | 1.85E-04 | 6.91E-03 | -1.80E+00 |  |
| Rnu2-10 | 3.45E+04 | 1.86E-04 | 6.92E-03 | 7.05E+00 |  |
| Proca1 | 1.67E+03 | 1.87E-04 | 6.93E-03 | -1.54E+00 |  |
| Tcea2 | 1.97E+03 | 1.87E-04 | 6.94E-03 | 1.61E+00 |  |
| 2610507B11Rik | 3.75E+03 | 1.88E-04 | 6.96E-03 | 1.57E+00 |  |
| Zfp37 | 3.37E+03 | 1.98E-04 | 7.30E-03 | -1.56E+00 |  |
| Prkag2os1 | 1.89E+02 | 2.02E-04 | 7.44E-03 | -4.19E+00 |  |
| Olfr10 | 3.84E+01 | 2.04E-04 | 7.51E-03 | -2.42E+01 |  |
| Eif4e2 | 1.81E+03 | 2.08E-04 | 7.62E-03 | 1.53E+00 |  |
| Gm43705 | 8.04E+01 | 2.09E-04 | 7.63E-03 | -2.25E+01 |  |
| Psg25 | 3.94E+01 | 2.09E-04 | 7.65E-03 | -3.13E+01 |  |
| 1700016H13Rik | 1.19E+03 | 2.11E-04 | 7.70E-03 | -1.80E+00 |  |
| Tepp | 1.62E+02 | 2.11E-04 | 7.70E-03 | -3.03E+00 |  |
| Olfr843 | 1.77E+02 | 2.17E-04 | 7.90E-03 | -5.07E+00 |  |
| Zfp941 | 7.52E+02 | 2.18E-04 | 7.91E-03 | -2.14E+00 |  |
| Adam5 | 8.74E+03 | 2.18E-04 | 7.91E-03 | 1.63E+00 |  |
| Gm4772 | 1.22E+02 | 2.19E-04 | 7.91E-03 | -3.79E+00 |  |
| Ppp2r2b | 3.33E+02 | 2.19E-04 | 7.91E-03 | -2.42E+00 |  |
| Nwd2 | 3.06E+02 | 2.23E-04 | 8.01E-03 | -2.46E+00 |  |
| Gm10532 | 1.51E+02 | 2.23E-04 | 8.01E-03 | -3.24E+00 |  |
| Lrrc47 | 7.59E+02 | 2.23E-04 | 8.01E-03 | 1.94E+00 |  |
| Lrrc8c | 2.41E+02 | 2.26E-04 | 8.10E-03 | -2.57E+00 |  |
| Dynlt1a | 5.49E+02 | 2.27E-04 | 8.13E-03 | 2.15E+00 |  |
| Cd8b1 | 4.09E+01 | 2.31E-04 | 8.22E-03 | -1.25E+01 |  |
| Ldha | 5.97E+03 | 2.31E-04 | 8.22E-03 | 1.55E+00 |  |
| Gm13340 | 1.56E+04 | 2.31E-04 | 8.22E-03 | 1.68E+00 |  |
| Tcp10c | 3.24E+03 | 2.31E-04 | 8.22E-03 | 1.53E+00 |  |
| Cacna1h | 1.74E+03 | 2.32E-04 | 8.23E-03 | 1.64E+00 |  |
| E230020D15Rik | 1.33E+02 | 2.33E-04 | 8.25E-03 | -4.99E+00 |  |
| Gm4922 | 8.44E+02 | 2.34E-04 | 8.25E-03 | -2.24E+00 |  |
| Ubb | 5.52E+03 | 2.34E-04 | 8.25E-03 | 1.56E+00 |  |
| Spag6l | 2.99E+03 | 2.35E-04 | 8.29E-03 | 1.62E+00 |  |
| Psmd2 | 1.90E+03 | 2.36E-04 | 8.31E-03 | 1.59E+00 |  |
| Gm43980 | 7.93E+02 | 2.39E-04 | 8.37E-03 | 1.82E+00 |  |
| Gm43285 | 6.09E+01 | 2.40E-04 | 8.40E-03 | -9.39E+00 |  |
| Jcad | 1.08E+03 | 2.41E-04 | 8.42E-03 | 1.79E+00 |  |
| Hspa2 | 8.77E+03 | 2.42E-04 | 8.45E-03 | 1.52E+00 |  |
| Klk1b8 | 2.41E+02 | 2.43E-04 | 8.46E-03 | -2.85E+00 |  |
| Hdac11 | 1.43E+03 | 2.44E-04 | 8.47E-03 | 1.79E+00 |  |
| Eef1g | 7.61E+02 | 2.46E-04 | 8.54E-03 | -1.69E+00 |  |
| Usp1 | 6.77E+03 | 2.47E-04 | 8.57E-03 | 1.50E+00 |  |
| Rora | 5.33E+02 | 2.49E-04 | 8.62E-03 | -2.01E+00 |  |
| Gm35835 | 1.09E+02 | 2.50E-04 | 8.63E-03 | -6.42E+00 |  |
| Auts2 | 3.26E+02 | 2.51E-04 | 8.67E-03 | -3.25E+00 |  |
| Hectd4 | 3.79E+03 | 2.52E-04 | 8.69E-03 | 1.51E+00 |  |
| 4930547H16Rik | 4.26E+02 | 2.53E-04 | 8.69E-03 | -2.26E+00 |  |
| Ypel1 | 2.45E+03 | 2.55E-04 | 8.76E-03 | 1.60E+00 |  |
| Snapc1 | 3.53E+02 | 2.55E-04 | 8.76E-03 | -2.16E+00 |  |
| Trmt13 | 3.62E+02 | 2.56E-04 | 8.76E-03 | -2.01E+00 |  |
| Stambpl1 | 6.26E+02 | 2.59E-04 | 8.86E-03 | -1.93E+00 |  |
| AL928857.1 | 5.38E+01 | 2.60E-04 | 8.88E-03 | 1.54E+01 |  |
| Tcea1-ps1 | 2.34E+02 | 2.66E-04 | 9.02E-03 | -2.31E+00 |  |
| Arid4a | 6.42E+02 | 2.67E-04 | 9.05E-03 | -1.90E+00 |  |
| Calm1 | 5.28E+03 | 2.68E-04 | 9.06E-03 | 1.57E+00 |  |
| Adamts15 | 9.56E+01 | 2.68E-04 | 9.06E-03 | -5.94E+00 |  |
| Dynlt1-ps1 | 1.44E+03 | 2.69E-04 | 9.09E-03 | 1.80E+00 |  |
| Zc3h10 | 6.24E+02 | 2.72E-04 | 9.16E-03 | -1.93E+00 |  |
| Hap1 | 4.19E+02 | 2.76E-04 | 9.28E-03 | 2.09E+00 |  |
| Gm8430 | 3.90E+02 | 2.78E-04 | 9.34E-03 | 2.65E+00 |  |
| Gm37488 | 5.08E+01 | 2.80E-04 | 9.37E-03 | -1.18E+01 |  |
| Gm26791 | 1.54E+02 | 2.81E-04 | 9.40E-03 | -2.86E+00 |  |
| Olfr635 | 2.47E+02 | 2.82E-04 | 9.40E-03 | -3.02E+00 |  |
| Mast4 | 1.28E+03 | 2.82E-04 | 9.41E-03 | -1.71E+00 |  |
| Snora70 | 5.09E+02 | 2.85E-04 | 9.50E-03 | 3.68E+00 |  |
| 1700093K21Rik | 2.09E+03 | 2.87E-04 | 9.53E-03 | -1.52E+00 |  |
| Cep164 | 2.03E+03 | 2.88E-04 | 9.54E-03 | 1.54E+00 |  |
| Gm40999 | 5.65E+01 | 2.88E-04 | 9.55E-03 | -8.77E+00 |  |
| Gm20631 | 6.33E+01 | 2.89E-04 | 9.57E-03 | -2.28E+01 |  |
| Gm37298 | 6.22E+01 | 2.93E-04 | 9.67E-03 | -9.35E+00 |  |
| Snx25 | 5.93E+02 | 2.94E-04 | 9.69E-03 | 2.03E+00 |  |
| Hkdc1 | 2.72E+01 | 2.99E-04 | 9.84E-03 | -2.51E+01 |  |
| Fam83e | 7.69E+02 | 2.99E-04 | 9.84E-03 | -1.78E+00 |  |
| Il1b | 7.85E+01 | 3.01E-04 | 9.88E-03 | 6.01E+00 |  |
| Gm44723 | 7.24E+01 | 3.04E-04 | 9.95E-03 | -1.43E+01 |  |
| Smok3c | 3.46E+02 | 3.05E-04 | 9.95E-03 | -3.46E+00 |  |
| Homer2 | 2.01E+02 | 3.05E-04 | 9.95E-03 | -6.10E+00 |  |
| Ms4a4b | 3.03E+02 | 3.06E-04 | 9.95E-03 | -2.62E+00 |  |
| Gsdmcl1 | 8.55E+01 | 3.06E-04 | 9.95E-03 | 8.30E+00 |  |
| Cep170b | 2.13E+02 | 3.06E-04 | 9.95E-03 | 2.82E+00 |  |
| 1810020O05Rik | 8.39E+02 | 3.07E-04 | 9.98E-03 | -1.98E+00 |  |
| Brca1 | 1.27E+03 | 3.08E-04 | 9.98E-03 | -1.83E+00 |  |
| Set | 3.16E+02 | 3.08E-04 | 9.98E-03 | 2.31E+00 |  |
| Rapgef5 | 4.40E+02 | 3.09E-04 | 9.99E-03 | -1.99E+00 |  |
| R3hcc1 | 4.81E+02 | 3.10E-04 | 9.99E-03 | -2.09E+00 |  |
| Gm45159 | 5.02E+02 | 3.13E-04 | 1.01E-02 | -2.39E+00 |  |
| 1700008P02Rik | 1.15E+02 | 3.14E-04 | 1.01E-02 | -5.76E+00 |  |
| Rbakdn | 1.07E+03 | 3.15E-04 | 1.01E-02 | 1.61E+00 |  |
| Ccdc150 | 2.16E+03 | 3.16E-04 | 1.01E-02 | -1.64E+00 |  |
| Car2 | 2.38E+02 | 3.16E-04 | 1.01E-02 | -2.76E+00 |  |
| Pex5l | 5.27E+02 | 3.17E-04 | 1.01E-02 | -1.85E+00 |  |
| Plekha8 | 7.97E+02 | 3.25E-04 | 1.04E-02 | 1.93E+00 |  |
| Dnajc9 | 8.07E+02 | 3.27E-04 | 1.04E-02 | -1.67E+00 |  |
| Gm37143 | 3.54E+01 | 3.28E-04 | 1.04E-02 | -4.01E+01 |  |
| 2610005L07Rik | 6.90E+02 | 3.28E-04 | 1.04E-02 | 1.72E+00 |  |
| Gm8228 | 2.40E+02 | 3.28E-04 | 1.04E-02 | -2.69E+00 |  |
| Gm3642 | 2.10E+02 | 3.29E-04 | 1.04E-02 | -3.24E+00 |  |
| Sppl2c | 1.05E+03 | 3.34E-04 | 1.05E-02 | -1.61E+00 |  |
| Cops7a | 7.54E+02 | 3.39E-04 | 1.07E-02 | 1.97E+00 |  |
| Gm11780 | 2.38E+02 | 3.41E-04 | 1.07E-02 | -2.67E+00 |  |
| Ptges3 | 3.17E+03 | 3.44E-04 | 1.08E-02 | 1.57E+00 |  |
| Vmn2r95 | 1.63E+02 | 3.45E-04 | 1.08E-02 | -4.43E+00 |  |
| Edrf1 | 6.85E+02 | 3.49E-04 | 1.09E-02 | -1.91E+00 |  |
| Scrib | 1.93E+03 | 3.50E-04 | 1.10E-02 | 1.60E+00 |  |
| Tmem196 | 7.76E+01 | 3.51E-04 | 1.10E-02 | -5.25E+00 |  |
| Olfr918 | 7.67E+01 | 3.53E-04 | 1.10E-02 | -8.02E+00 |  |
| 4930448A20Rik | 2.51E+02 | 3.55E-04 | 1.10E-02 | -3.33E+00 |  |
| Olfr1205 | 8.98E+01 | 3.56E-04 | 1.10E-02 | -8.21E+00 |  |
| AC124459.1 | 1.40E+02 | 3.56E-04 | 1.10E-02 | -4.80E+00 |  |
| Gm30302 | 8.67E+02 | 3.56E-04 | 1.10E-02 | -1.67E+00 |  |
| 4930431P03Rik | 7.99E+02 | 3.56E-04 | 1.10E-02 | 1.92E+00 |  |
| Mael | 3.67E+03 | 3.56E-04 | 1.10E-02 | 1.50E+00 |  |
| Olfr1247 | 1.38E+02 | 3.59E-04 | 1.11E-02 | -4.66E+00 |  |
| 1700024P04Rik | 4.89E+02 | 3.61E-04 | 1.11E-02 | -3.17E+00 |  |
| Snord8 | 4.09E+02 | 3.65E-04 | 1.12E-02 | 2.76E+00 |  |
| Gm42609 | 1.43E+03 | 3.65E-04 | 1.12E-02 | -1.65E+00 |  |
| Spata4 | 3.85E+03 | 3.65E-04 | 1.12E-02 | 1.60E+00 |  |
| Ptgfrn | 1.33E+02 | 3.70E-04 | 1.13E-02 | -6.51E+00 |  |
| Rtn4 | 1.03E+03 | 3.72E-04 | 1.14E-02 | 1.59E+00 |  |
| BC106175 | 4.31E+03 | 3.72E-04 | 1.14E-02 | -1.51E+00 |  |
| 2810408A11Rik | 1.24E+03 | 3.75E-04 | 1.15E-02 | -1.90E+00 |  |
| Pip5k1a | 5.56E+02 | 3.77E-04 | 1.15E-02 | -2.70E+00 |  |
| Ankrd53 | 4.03E+02 | 3.80E-04 | 1.16E-02 | -2.14E+00 |  |
| Gm10184 | 1.35E+03 | 3.82E-04 | 1.16E-02 | 1.92E+00 |  |
| Vmn2r23 | 2.48E+02 | 3.87E-04 | 1.17E-02 | -3.35E+00 |  |
| Efhd1 | 2.30E+03 | 3.87E-04 | 1.17E-02 | 1.80E+00 |  |
| Ell2 | 9.15E+02 | 3.98E-04 | 1.20E-02 | -1.67E+00 |  |
| Olfr679 | 7.99E+01 | 3.98E-04 | 1.20E-02 | -5.79E+00 |  |
| Ppp1r2-ps6 | 1.30E+03 | 3.99E-04 | 1.20E-02 | -1.88E+00 |  |
| Ttc21b | 1.99E+03 | 4.04E-04 | 1.21E-02 | 1.51E+00 |  |
| Actl11 | 1.92E+03 | 4.05E-04 | 1.21E-02 | -1.66E+00 |  |
| Gm41279 | 1.94E+02 | 4.05E-04 | 1.21E-02 | -3.64E+00 |  |
| Gm5458 | 1.09E+03 | 4.05E-04 | 1.21E-02 | -2.32E+00 |  |
| Depdc1a | 7.11E+01 | 4.07E-04 | 1.22E-02 | -1.28E+01 |  |
| Col9a3 | 1.87E+02 | 4.10E-04 | 1.22E-02 | 3.28E+00 |  |
| Gm3531 | 7.28E+02 | 4.11E-04 | 1.22E-02 | 1.82E+00 |  |
| Slc2a5 | 2.96E+03 | 4.11E-04 | 1.22E-02 | 1.51E+00 |  |
| Srxn1 | 2.74E+02 | 4.12E-04 | 1.22E-02 | -2.11E+00 |  |
| Snx6 | 4.39E+01 | 4.13E-04 | 1.23E-02 | 2.99E+01 |  |
| Agpat5 | 1.54E+02 | 4.14E-04 | 1.23E-02 | -3.62E+00 |  |
| Lrp5 | 1.03E+03 | 4.16E-04 | 1.23E-02 | -1.71E+00 |  |
| Vmn1r184 | 1.25E+02 | 4.18E-04 | 1.23E-02 | -5.94E+00 |  |
| Fam131b | 6.27E+01 | 4.18E-04 | 1.23E-02 | -7.08E+00 |  |
| Pcbp3 | 1.31E+03 | 4.21E-04 | 1.24E-02 | 1.61E+00 |  |
| Gm5662 | 5.36E+01 | 4.23E-04 | 1.24E-02 | -3.60E+01 |  |
| Tedc1 | 5.35E+02 | 4.26E-04 | 1.25E-02 | 2.16E+00 |  |
| Fmo2 | 4.71E+01 | 4.27E-04 | 1.25E-02 | -1.44E+01 |  |
| Gm12238 | 4.95E+02 | 4.28E-04 | 1.25E-02 | 2.16E+00 |  |
| Hsdl2 | 1.45E+03 | 4.30E-04 | 1.26E-02 | -1.67E+00 |  |
| Bbs4 | 7.76E+02 | 4.31E-04 | 1.26E-02 | -1.80E+00 |  |
| Olfr810 | 6.30E+01 | 4.34E-04 | 1.26E-02 | -1.55E+01 |  |
| Snora62 | 1.12E+03 | 4.34E-04 | 1.26E-02 | 1.87E+00 |  |
| Leng8 | 3.70E+03 | 4.38E-04 | 1.27E-02 | 1.57E+00 |  |
| Boll | 1.73E+03 | 4.43E-04 | 1.29E-02 | 1.56E+00 |  |
| Gm33948 | 5.57E+01 | 4.44E-04 | 1.29E-02 | -7.04E+00 |  |
| 1700010J16Rik | 2.77E+02 | 4.46E-04 | 1.29E-02 | -2.69E+00 |  |
| Tmem88 | 7.74E+01 | 4.48E-04 | 1.29E-02 | -6.43E+00 |  |
| Gm14326 | 4.35E+01 | 4.48E-04 | 1.29E-02 | 1.92E+01 |  |
| Evl | 1.26E+02 | 4.48E-04 | 1.29E-02 | 4.31E+00 |  |
| Polh | 9.92E+02 | 4.51E-04 | 1.30E-02 | -1.77E+00 |  |
| Gm35769 | 5.62E+01 | 4.56E-04 | 1.31E-02 | -1.45E+01 |  |
| 4930547E14Rik | 2.32E+02 | 4.59E-04 | 1.32E-02 | -2.78E+00 |  |
| Acsbg2 | 2.15E+03 | 4.61E-04 | 1.32E-02 | -1.62E+00 |  |
| Selenos | 1.07E+03 | 4.61E-04 | 1.32E-02 | 1.61E+00 |  |
| Eif4ebp2 | 1.06E+03 | 4.63E-04 | 1.32E-02 | 1.76E+00 |  |
| Rps3a2 | 3.13E+02 | 4.65E-04 | 1.32E-02 | 2.70E+00 |  |
| Vash2 | 1.84E+03 | 4.65E-04 | 1.32E-02 | -1.58E+00 |  |
| Lin9 | 6.96E+02 | 4.66E-04 | 1.32E-02 | -1.74E+00 |  |
| Cmpk2 | 2.94E+02 | 4.67E-04 | 1.32E-02 | -2.49E+00 |  |
| Snora16a | 1.68E+02 | 4.68E-04 | 1.32E-02 | 6.12E+00 |  |
| Snora34 | 2.87E+02 | 4.75E-04 | 1.34E-02 | 3.91E+00 |  |
| Acot7 | 1.65E+03 | 4.79E-04 | 1.35E-02 | 1.69E+00 |  |
| Gm23442 | 6.93E+02 | 4.80E-04 | 1.35E-02 | 2.23E+00 |  |
| Ppp4r3a | 1.29E+03 | 4.80E-04 | 1.35E-02 | -1.63E+00 |  |
| Usp24 | 1.56E+03 | 4.81E-04 | 1.35E-02 | 1.57E+00 |  |
| Rgs12 | 8.49E+02 | 4.83E-04 | 1.35E-02 | 1.66E+00 |  |
| Vmn1r39 | 3.36E+01 | 4.88E-04 | 1.37E-02 | -3.80E+01 |  |
| Rbmxl1 | 3.83E+02 | 4.92E-04 | 1.37E-02 | -2.23E+00 |  |
| Spef1 | 3.85E+02 | 4.93E-04 | 1.37E-02 | -2.03E+00 |  |
| Gm45504 | 4.43E+01 | 4.93E-04 | 1.37E-02 | -2.39E+01 |  |
| Gm28661 | 7.04E+03 | 4.94E-04 | 1.38E-02 | 1.75E+00 |  |
| Rps12-ps3 | 1.80E+02 | 4.95E-04 | 1.38E-02 | 6.08E+00 |  |
| Gm26578 | 5.38E+01 | 4.95E-04 | 1.38E-02 | -1.90E+01 |  |
| A130010J15Rik | 3.60E+02 | 4.98E-04 | 1.38E-02 | -2.44E+00 |  |
| Sirt4 | 3.23E+02 | 5.01E-04 | 1.39E-02 | -2.71E+00 |  |
| Vmn1r81 | 1.46E+02 | 5.06E-04 | 1.40E-02 | -3.74E+00 |  |
| Jakmip2 | 1.74E+02 | 5.13E-04 | 1.42E-02 | -2.80E+00 |  |
| Copb2 | 6.68E+02 | 5.15E-04 | 1.42E-02 | 2.00E+00 |  |
| Gm8165 | 8.28E+01 | 5.15E-04 | 1.42E-02 | -5.02E+00 |  |
| Zkscan17 | 1.24E+03 | 5.17E-04 | 1.42E-02 | 1.60E+00 |  |
| Gm6351 | 9.03E+01 | 5.20E-04 | 1.43E-02 | -4.64E+00 |  |
| Pcsk5 | 8.08E+01 | 5.24E-04 | 1.44E-02 | -5.34E+00 |  |
| Lig3 | 2.14E+03 | 5.31E-04 | 1.46E-02 | 1.61E+00 |  |
| Lrp1b | 2.63E+02 | 5.31E-04 | 1.46E-02 | -2.57E+00 |  |
| Gm21759 | 6.80E+02 | 5.38E-04 | 1.47E-02 | -1.96E+00 |  |
| Gm136 | 4.23E+02 | 5.40E-04 | 1.48E-02 | -2.12E+00 |  |
| Gm3542 | 3.17E+02 | 5.43E-04 | 1.48E-02 | -3.18E+00 |  |
| Lyzl4os | 4.93E+02 | 5.44E-04 | 1.48E-02 | -1.99E+00 |  |
| Gm34004 | 4.21E+01 | 5.46E-04 | 1.48E-02 | -1.38E+01 |  |
| Dvl1 | 1.25E+03 | 5.46E-04 | 1.48E-02 | 1.67E+00 |  |
| Cst8 | 1.14E+03 | 5.47E-04 | 1.48E-02 | -1.67E+00 |  |
| Hsbp1 | 1.93E+03 | 5.50E-04 | 1.49E-02 | 1.52E+00 |  |
| Olfr551 | 5.22E+01 | 5.51E-04 | 1.49E-02 | -1.77E+01 |  |
| Zfp330 | 7.71E+02 | 5.58E-04 | 1.51E-02 | -1.69E+00 |  |
| Actl9 | 1.28E+03 | 5.58E-04 | 1.51E-02 | -1.61E+00 |  |
| D930036K23Rik | 5.59E+01 | 5.64E-04 | 1.52E-02 | -8.21E+00 |  |
| Olfr1307 | 2.38E+02 | 5.64E-04 | 1.52E-02 | -2.66E+00 |  |
| Zfp493 | 1.70E+02 | 5.65E-04 | 1.52E-02 | -4.41E+00 |  |
| Olfr878 | 5.81E+01 | 5.67E-04 | 1.52E-02 | -2.44E+01 |  |
| Afap1 | 5.64E+02 | 5.70E-04 | 1.53E-02 | 1.96E+00 |  |
| Il1rap | 2.23E+02 | 5.73E-04 | 1.54E-02 | -3.51E+00 |  |
| Tnfaip8l3 | 6.66E+01 | 5.75E-04 | 1.54E-02 | -6.61E+00 |  |
| D1Ertd622e | 9.76E+02 | 5.78E-04 | 1.54E-02 | -1.99E+00 |  |
| Rcn2 | 4.32E+02 | 5.82E-04 | 1.55E-02 | 2.23E+00 |  |
| Hikeshi | 1.95E+02 | 5.87E-04 | 1.56E-02 | -3.56E+00 |  |
| Gm37269 | 3.94E+01 | 5.87E-04 | 1.56E-02 | -2.65E+01 |  |
| Sfmbt1 | 2.31E+03 | 5.92E-04 | 1.57E-02 | 1.51E+00 |  |
| Rasgef1b | 2.13E+02 | 5.97E-04 | 1.59E-02 | -3.24E+00 |  |
| Gm44673 | 3.52E+01 | 5.99E-04 | 1.59E-02 | -2.81E+01 |  |
| Gm38195 | 2.96E+01 | 6.03E-04 | 1.60E-02 | -4.87E+01 |  |
| Snora24 | 3.31E+02 | 6.05E-04 | 1.60E-02 | 4.13E+00 |  |
| Stim1 | 1.42E+03 | 6.07E-04 | 1.60E-02 | 1.58E+00 |  |
| AC133601.1 | 5.55E+01 | 6.08E-04 | 1.60E-02 | -2.61E+01 |  |
| Ktn1 | 1.49E+02 | 6.11E-04 | 1.61E-02 | -4.28E+00 |  |
| C2cd3 | 2.56E+03 | 6.13E-04 | 1.61E-02 | -1.51E+00 |  |
| Tacr3 | 4.16E+01 | 6.14E-04 | 1.61E-02 | -1.25E+01 |  |
| Phactr1 | 3.83E+02 | 6.18E-04 | 1.62E-02 | -2.07E+00 |  |
| 4930579F01Rik | 8.95E+02 | 6.20E-04 | 1.62E-02 | -1.70E+00 |  |
| Entpd7 | 1.19E+02 | 6.21E-04 | 1.63E-02 | -5.28E+00 |  |
| Ccdc54 | 2.19E+03 | 6.22E-04 | 1.63E-02 | -1.69E+00 |  |
| Pde3b | 1.37E+03 | 6.23E-04 | 1.63E-02 | 1.54E+00 |  |
| Nup160 | 9.89E+02 | 6.23E-04 | 1.63E-02 | -1.71E+00 |  |
| Gm23287 | 5.09E+02 | 6.27E-04 | 1.63E-02 | 3.50E+01 |  |
| Tmco5b | 3.53E+02 | 6.28E-04 | 1.63E-02 | -1.99E+00 |  |
| Aldoart2 | 1.36E+03 | 6.30E-04 | 1.64E-02 | -1.56E+00 |  |
| Phospho1 | 4.52E+02 | 6.32E-04 | 1.64E-02 | -2.08E+00 |  |
| Snora17 | 3.37E+02 | 6.32E-04 | 1.64E-02 | 3.27E+00 |  |
| Mad2l2 | 5.84E+02 | 6.37E-04 | 1.65E-02 | 2.25E+00 |  |
| Gm10807 | 8.18E+01 | 6.40E-04 | 1.65E-02 | -4.07E+00 |  |
| Gm34678 | 7.25E+02 | 6.41E-04 | 1.65E-02 | 2.14E+00 |  |
| Snora23 | 1.10E+03 | 6.41E-04 | 1.65E-02 | 1.91E+00 |  |
| Sptbn1 | 3.36E+03 | 6.44E-04 | 1.66E-02 | -1.50E+00 |  |
| Olfr881 | 7.77E+01 | 6.44E-04 | 1.66E-02 | -9.79E+00 |  |
| Gm553 | 9.88E+01 | 6.47E-04 | 1.66E-02 | -8.63E+00 |  |
| Gm19589 | 1.08E+02 | 6.49E-04 | 1.67E-02 | -5.15E+00 |  |
| Itga2 | 1.95E+02 | 6.50E-04 | 1.67E-02 | -2.53E+00 |  |
| Cpq | 1.52E+02 | 6.53E-04 | 1.67E-02 | 4.17E+00 |  |
| Speer1 | 3.45E+02 | 6.56E-04 | 1.68E-02 | -2.31E+00 |  |
| Hpn | 4.76E+01 | 6.60E-04 | 1.69E-02 | -1.25E+01 |  |
| Gm2237 | 2.41E+02 | 6.66E-04 | 1.70E-02 | -2.85E+00 |  |
| Gm44676 | 7.28E+01 | 6.67E-04 | 1.70E-02 | -4.52E+00 |  |
| Snora52 | 2.06E+02 | 6.73E-04 | 1.71E-02 | 2.68E+00 |  |
| Fryl | 6.03E+02 | 6.73E-04 | 1.71E-02 | 1.74E+00 |  |
| Neto1 | 1.34E+02 | 6.74E-04 | 1.71E-02 | -4.10E+00 |  |
| Rasl2-9 | 6.30E+02 | 6.74E-04 | 1.71E-02 | 1.78E+00 |  |
| Snord22 | 4.82E+03 | 6.74E-04 | 1.71E-02 | 4.40E+00 |  |
| Pwwp3a | 3.53E+03 | 6.74E-04 | 1.71E-02 | 1.51E+00 |  |
| Gm8116 | 2.40E+02 | 6.77E-04 | 1.71E-02 | 2.50E+00 |  |
| 4930432E11Rik | 3.51E+03 | 6.78E-04 | 1.71E-02 | -1.87E+00 |  |
| Olfr78 | 1.01E+02 | 6.84E-04 | 1.72E-02 | -8.75E+00 |  |
| Olfr168 | 2.87E+01 | 6.84E-04 | 1.72E-02 | -4.69E+01 |  |
| Gm25835 | 3.68E+03 | 6.84E-04 | 1.72E-02 | 3.84E+00 |  |
| Gm37345 | 7.15E+01 | 6.84E-04 | 1.72E-02 | -6.78E+00 |  |
| Asprv1 | 7.35E+01 | 6.86E-04 | 1.72E-02 | 7.12E+00 |  |
| Klf9 | 1.16E+03 | 6.92E-04 | 1.74E-02 | -2.00E+00 |  |
| 1700042G07Rik | 1.75E+02 | 6.92E-04 | 1.74E-02 | -2.65E+00 |  |
| Htt | 2.17E+03 | 6.92E-04 | 1.74E-02 | 1.51E+00 |  |
| Wnk1 | 8.25E+03 | 7.04E-04 | 1.76E-02 | 1.57E+00 |  |
| Ptprs | 1.03E+03 | 7.06E-04 | 1.76E-02 | 1.64E+00 |  |
| Cdh10 | 1.06E+02 | 7.07E-04 | 1.76E-02 | -7.05E+00 |  |
| Slc35f1 | 7.73E+01 | 7.09E-04 | 1.77E-02 | -1.27E+01 |  |
| Baiap2 | 7.08E+02 | 7.10E-04 | 1.77E-02 | 1.65E+00 |  |
| Oplah | 6.90E+02 | 7.10E-04 | 1.77E-02 | -1.68E+00 |  |
| Setdb1 | 9.92E+02 | 7.12E-04 | 1.77E-02 | 1.62E+00 |  |
| Gm29681 | 3.28E+01 | 7.13E-04 | 1.77E-02 | -1.27E+01 |  |
| Crkl | 8.19E+02 | 7.14E-04 | 1.77E-02 | -1.74E+00 |  |
| Spo11 | 7.06E+02 | 7.16E-04 | 1.77E-02 | 1.77E+00 |  |
| Eif3l | 6.31E+02 | 7.16E-04 | 1.77E-02 | 1.94E+00 |  |
| Gm7600 | 7.36E+01 | 7.19E-04 | 1.78E-02 | 6.86E+00 |  |
| Rasgrf2 | 6.12E+01 | 7.22E-04 | 1.78E-02 | -6.01E+00 |  |
| Snora64 | 4.58E+02 | 7.24E-04 | 1.78E-02 | 2.75E+00 |  |
| Vmn1r171 | 6.49E+01 | 7.24E-04 | 1.78E-02 | -1.60E+01 |  |
| H2afz | 7.76E+02 | 7.27E-04 | 1.79E-02 | 1.80E+00 |  |
| Snord16a | 1.12E+03 | 7.27E-04 | 1.79E-02 | 4.74E+00 |  |
| Ints10 | 1.30E+03 | 7.28E-04 | 1.79E-02 | 1.53E+00 |  |
| Gm16726 | 2.95E+02 | 7.35E-04 | 1.80E-02 | -2.20E+00 |  |
| Dnah11 | 1.16E+02 | 7.38E-04 | 1.81E-02 | 3.82E+00 |  |
| Gm24601 | 3.13E+03 | 7.38E-04 | 1.81E-02 | 2.90E+00 |  |
| Cd300a | 7.00E+01 | 7.40E-04 | 1.81E-02 | -7.41E+00 |  |
| Atrnl1 | 3.46E+02 | 7.41E-04 | 1.81E-02 | -2.30E+00 |  |
| Gm22265 | 1.61E+03 | 7.45E-04 | 1.82E-02 | 2.41E+01 |  |
| 2310002L09Rik | 3.12E+01 | 7.49E-04 | 1.82E-02 | -3.43E+01 |  |
| Gm41206 | 2.81E+02 | 7.53E-04 | 1.83E-02 | -2.96E+00 |  |
| Acr | 1.09E+03 | 7.59E-04 | 1.84E-02 | 1.56E+00 |  |
| Snora28 | 1.96E+03 | 7.62E-04 | 1.85E-02 | 3.49E+00 |  |
| Chpt1 | 1.05E+03 | 7.65E-04 | 1.85E-02 | -1.69E+00 |  |
| Ccdc169 | 1.07E+03 | 7.66E-04 | 1.85E-02 | -1.55E+00 |  |
| Mical3 | 1.61E+03 | 7.71E-04 | 1.87E-02 | -1.57E+00 |  |
| 1700095B10Rik | 4.34E+02 | 7.74E-04 | 1.87E-02 | -2.01E+00 |  |
| Gm12295 | 4.14E+01 | 7.77E-04 | 1.87E-02 | -1.15E+01 |  |
| Plk2 | 2.27E+02 | 7.77E-04 | 1.87E-02 | -3.48E+00 |  |
| Atp10a | 1.21E+03 | 7.78E-04 | 1.87E-02 | -1.74E+00 |  |
| Shcbp1 | 1.14E+03 | 7.79E-04 | 1.87E-02 | -1.65E+00 |  |
| Sccpdh | 3.20E+03 | 7.84E-04 | 1.88E-02 | 1.59E+00 |  |
| Olfr1297 | 1.39E+02 | 7.84E-04 | 1.88E-02 | -5.22E+00 |  |
| 1700012B07Rik | 1.56E+03 | 7.86E-04 | 1.89E-02 | -1.56E+00 |  |
| Reep2 | 4.47E+02 | 7.94E-04 | 1.90E-02 | 1.97E+00 |  |
| Gstm1 | 9.89E+01 | 7.94E-04 | 1.90E-02 | 5.35E+00 |  |
| Rnu1a1 | 2.81E+02 | 7.99E-04 | 1.91E-02 | 3.48E+00 |  |
| 4930572O13Rik | 8.96E+01 | 8.01E-04 | 1.91E-02 | -4.36E+00 |  |
| Olfr630 | 3.07E+01 | 8.03E-04 | 1.91E-02 | -1.49E+01 |  |
| 4930545H06Rik | 1.72E+02 | 8.04E-04 | 1.91E-02 | -3.23E+00 |  |
| Gm21671 | 1.66E+02 | 8.08E-04 | 1.92E-02 | -5.38E+00 |  |
| Pi4ka | 1.06E+03 | 8.09E-04 | 1.92E-02 | 1.57E+00 |  |
| Olfr1264 | 6.36E+01 | 8.11E-04 | 1.93E-02 | -4.39E+00 |  |
| Gm8126 | 4.55E+02 | 8.13E-04 | 1.93E-02 | -2.45E+00 |  |
| Gm3417 | 2.14E+03 | 8.29E-04 | 1.96E-02 | 1.86E+00 |  |
| Gm6117 | 6.43E+02 | 8.30E-04 | 1.96E-02 | -1.91E+00 |  |
| Ncbp1 | 2.50E+03 | 8.37E-04 | 1.98E-02 | 1.55E+00 |  |
| Bzw1 | 1.17E+03 | 8.37E-04 | 1.98E-02 | -1.65E+00 |  |
| Olfr1023 | 4.86E+01 | 8.42E-04 | 1.98E-02 | -1.23E+01 |  |
| Cxxc5 | 7.08E+02 | 8.43E-04 | 1.99E-02 | -1.82E+00 |  |
| Gm37730 | 3.06E+01 | 8.46E-04 | 1.99E-02 | -3.35E+01 |  |
| Glul | 6.71E+02 | 8.47E-04 | 1.99E-02 | -1.65E+00 |  |
| Ttc12 | 9.07E+02 | 8.47E-04 | 1.99E-02 | 1.54E+00 |  |
| Gm36814 | 2.44E+02 | 8.49E-04 | 1.99E-02 | -2.73E+00 |  |
| Ghr | 1.37E+02 | 8.57E-04 | 2.01E-02 | -2.81E+00 |  |
| Prox2os | 1.24E+02 | 8.59E-04 | 2.01E-02 | 4.06E+00 |  |
| Zfp784 | 8.02E+01 | 8.61E-04 | 2.01E-02 | 1.28E+01 |  |
| Plekho1 | 1.01E+02 | 8.76E-04 | 2.04E-02 | 4.41E+00 |  |
| Fam71f2 | 5.46E+02 | 8.77E-04 | 2.04E-02 | -1.90E+00 |  |
| Tomm6os | 6.54E+01 | 8.79E-04 | 2.04E-02 | 7.61E+00 |  |
| Gm26939 | 1.29E+02 | 8.85E-04 | 2.05E-02 | -4.82E+00 |  |
| Dixdc1 | 1.17E+03 | 8.86E-04 | 2.06E-02 | 1.62E+00 |  |
| C630028M04Rik | 6.94E+01 | 8.91E-04 | 2.06E-02 | -1.20E+01 |  |
| 1700048M11Rik | 3.68E+01 | 8.91E-04 | 2.06E-02 | -1.47E+01 |  |
| Ilrun | 5.33E+02 | 8.91E-04 | 2.06E-02 | -1.88E+00 |  |
| Ccr2 | 6.53E+01 | 9.01E-04 | 2.08E-02 | -4.45E+00 |  |
| Gm25820 | 8.24E+02 | 9.02E-04 | 2.08E-02 | 2.10E+00 |  |
| Gm45461 | 1.06E+02 | 9.04E-04 | 2.08E-02 | -6.53E+00 |  |
| 4930529C04Rik | 1.98E+02 | 9.07E-04 | 2.08E-02 | -3.56E+00 |  |
| D830030K20Rik | 1.02E+02 | 9.26E-04 | 2.12E-02 | 5.26E+00 |  |
| Rbms3 | 1.02E+02 | 9.28E-04 | 2.12E-02 | -4.96E+00 |  |
| Spast | 8.80E+02 | 9.28E-04 | 2.12E-02 | 1.68E+00 |  |
| Homez | 1.22E+03 | 9.29E-04 | 2.12E-02 | -1.69E+00 |  |
| Gm24920 | 1.34E+02 | 9.31E-04 | 2.12E-02 | 7.96E+00 |  |
| Olfr1170 | 4.88E+01 | 9.31E-04 | 2.12E-02 | -1.93E+01 |  |
| Snora41 | 1.63E+02 | 9.33E-04 | 2.12E-02 | 6.64E+00 |  |
| Gm15530 | 1.84E+02 | 9.34E-04 | 2.13E-02 | -2.67E+00 |  |
| Slc44a5 | 2.88E+02 | 9.38E-04 | 2.13E-02 | -2.31E+00 |  |
| 4921504E06Rik | 1.00E+03 | 9.38E-04 | 2.13E-02 | -1.85E+00 |  |
| Gm25632 | 7.96E+01 | 9.38E-04 | 2.13E-02 | 9.95E+00 |  |
| Gm25313 | 1.36E+04 | 9.38E-04 | 2.13E-02 | 1.51E+01 |  |
| Gm24134 | 2.07E+02 | 9.39E-04 | 2.13E-02 | 1.41E+02 |  |
| 4930442L01Rik | 6.02E+02 | 9.49E-04 | 2.15E-02 | -2.00E+00 |  |
| Cfhr1 | 1.45E+02 | 9.51E-04 | 2.15E-02 | -2.73E+00 |  |
| Snora74a | 2.09E+02 | 9.54E-04 | 2.15E-02 | 4.59E+00 |  |
| Lims1 | 2.89E+02 | 9.54E-04 | 2.15E-02 | 2.18E+00 |  |
| Jmy | 1.22E+03 | 9.58E-04 | 2.16E-02 | -1.74E+00 |  |
| Tbc1d21 | 1.09E+03 | 9.65E-04 | 2.17E-02 | -1.92E+00 |  |
| Tmem200a | 2.58E+02 | 9.74E-04 | 2.19E-02 | -2.65E+00 |  |
| Vmn1r74 | 1.10E+02 | 9.77E-04 | 2.19E-02 | -5.92E+00 |  |
| Mllt1 | 8.76E+02 | 9.79E-04 | 2.20E-02 | 1.74E+00 |  |
| Bsg | 2.39E+03 | 9.84E-04 | 2.20E-02 | 1.63E+00 |  |
| Fbxo21 | 1.35E+02 | 9.84E-04 | 2.20E-02 | -3.05E+00 |  |
| Rnf138 | 1.95E+03 | 9.91E-04 | 2.21E-02 | -1.59E+00 |  |
| Spaca9 | 6.39E+02 | 9.96E-04 | 2.22E-02 | -1.91E+00 |  |
| 4933417O13Rik | 4.10E+02 | 9.99E-04 | 2.22E-02 | -2.58E+00 |  |
| Gm15581 | 6.91E+01 | 1.00E-03 | 2.23E-02 | -4.54E+00 |  |
| Psmd12 | 4.78E+02 | 1.01E-03 | 2.24E-02 | 1.83E+00 |  |
| Nup133 | 5.97E+02 | 1.01E-03 | 2.25E-02 | 1.87E+00 |  |
| Tssk6 | 2.03E+02 | 1.02E-03 | 2.26E-02 | -2.87E+00 |  |
| Vmn1r172 | 1.44E+02 | 1.02E-03 | 2.26E-02 | -5.29E+00 |  |
| Grin2a | 1.86E+02 | 1.02E-03 | 2.26E-02 | -2.95E+00 |  |
| Vmn2r94 | 1.85E+02 | 1.03E-03 | 2.27E-02 | -5.85E+00 |  |
| Snord14d | 1.95E+02 | 1.03E-03 | 2.28E-02 | 4.54E+00 |  |
| Etv1 | 1.72E+02 | 1.04E-03 | 2.30E-02 | -3.42E+00 |  |
| Gm45493 | 3.39E+01 | 1.04E-03 | 2.30E-02 | -1.21E+01 |  |
| Mif4gd | 1.38E+03 | 1.05E-03 | 2.31E-02 | 1.51E+00 |  |
| Ide | 2.91E+03 | 1.05E-03 | 2.31E-02 | 1.51E+00 |  |
| Gm5114 | 1.50E+03 | 1.07E-03 | 2.34E-02 | -1.98E+00 |  |
| Gm8212 | 1.34E+02 | 1.07E-03 | 2.34E-02 | -4.86E+00 |  |
| Il5ra | 7.37E+01 | 1.07E-03 | 2.34E-02 | -5.23E+00 |  |
| Zfand3 | 1.30E+03 | 1.07E-03 | 2.34E-02 | -1.52E+00 |  |
| Mylk4 | 1.92E+02 | 1.07E-03 | 2.35E-02 | -3.00E+00 |  |
| Olfr1042 | 6.87E+01 | 1.07E-03 | 2.35E-02 | -1.28E+01 |  |
| Spata2l | 5.05E+02 | 1.08E-03 | 2.36E-02 | -1.90E+00 |  |
| Ccdc27 | 9.35E+02 | 1.08E-03 | 2.36E-02 | -1.63E+00 |  |
| Dab2ip | 9.86E+02 | 1.08E-03 | 2.36E-02 | -1.68E+00 |  |
| Gm6367 | 7.02E+01 | 1.08E-03 | 2.36E-02 | -1.44E+01 |  |
| Ube2d2a | 1.41E+03 | 1.08E-03 | 2.36E-02 | 1.51E+00 |  |
| Olfr372 | 6.87E+01 | 1.08E-03 | 2.36E-02 | -1.18E+01 |  |
| Gm8046 | 3.39E+01 | 1.08E-03 | 2.36E-02 | 2.26E+01 |  |
| Lrp12 | 1.96E+02 | 1.09E-03 | 2.36E-02 | 2.78E+00 |  |
| Fancd2 | 5.88E+02 | 1.09E-03 | 2.36E-02 | 1.79E+00 |  |
| Vgll3 | 4.61E+02 | 1.09E-03 | 2.36E-02 | -1.82E+00 |  |
| Rpn1 | 1.04E+03 | 1.09E-03 | 2.37E-02 | 1.53E+00 |  |
| Gtf2i | 2.13E+03 | 1.10E-03 | 2.37E-02 | 1.51E+00 |  |
| Ndnf | 7.67E+01 | 1.10E-03 | 2.37E-02 | -4.68E+00 |  |
| Cdk16 | 4.18E+02 | 1.10E-03 | 2.38E-02 | -2.08E+00 |  |
| Rnaseh2c | 8.08E+01 | 1.11E-03 | 2.39E-02 | 9.25E+00 |  |
| Gm10309 | 2.00E+02 | 1.11E-03 | 2.40E-02 | -2.86E+00 |  |
| Bambi | 3.86E+02 | 1.12E-03 | 2.40E-02 | -2.22E+00 |  |
| Hfm1 | 7.30E+02 | 1.12E-03 | 2.41E-02 | 1.66E+00 |  |
| Arpp21 | 1.38E+02 | 1.14E-03 | 2.44E-02 | -3.70E+00 |  |
| Gm19872 | 2.30E+02 | 1.14E-03 | 2.44E-02 | -2.34E+00 |  |
| Rny3 | 2.33E+02 | 1.14E-03 | 2.45E-02 | 3.24E+00 |  |
| Nyap2 | 1.17E+02 | 1.14E-03 | 2.45E-02 | -3.01E+00 |  |
| Rbm6-ps1 | 2.22E+02 | 1.15E-03 | 2.46E-02 | -2.40E+00 |  |
| Lars | 3.48E+02 | 1.15E-03 | 2.47E-02 | 2.00E+00 |  |
| Snora65 | 2.30E+03 | 1.16E-03 | 2.47E-02 | 3.62E+00 |  |
| Synpo2 | 1.69E+02 | 1.16E-03 | 2.48E-02 | -3.42E+00 |  |
| Mrps31 | 2.18E+02 | 1.17E-03 | 2.49E-02 | 2.71E+00 |  |
| Taf9 | 2.79E+03 | 1.17E-03 | 2.49E-02 | 1.80E+00 |  |
| Gm14144 | 1.64E+03 | 1.18E-03 | 2.50E-02 | 1.59E+00 |  |
| Snora5c | 2.83E+02 | 1.18E-03 | 2.50E-02 | 3.11E+00 |  |
| Gm10157 | 9.27E+02 | 1.19E-03 | 2.52E-02 | -1.81E+00 |  |
| Zfp40 | 7.13E+01 | 1.19E-03 | 2.53E-02 | -4.70E+00 |  |
| Gm36368 | 3.34E+02 | 1.22E-03 | 2.57E-02 | -2.43E+00 |  |
| A230004M16Rik | 1.03E+02 | 1.22E-03 | 2.57E-02 | -1.19E+01 |  |
| Lamb2 | 6.63E+01 | 1.22E-03 | 2.57E-02 | 6.79E+00 |  |
| Gm42888 | 5.37E+01 | 1.22E-03 | 2.57E-02 | -1.41E+01 |  |
| Olfr1384 | 6.79E+01 | 1.22E-03 | 2.57E-02 | -1.27E+01 |  |
| Tmem81 | 5.64E+02 | 1.22E-03 | 2.57E-02 | -2.10E+00 |  |
| 2810006K23Rik | 2.00E+02 | 1.23E-03 | 2.58E-02 | -3.82E+00 |  |
| Rnf25 | 5.89E+02 | 1.24E-03 | 2.59E-02 | -1.76E+00 |  |
| Cdh2 | 1.49E+03 | 1.24E-03 | 2.60E-02 | 1.82E+00 |  |
| Gm14245 | 4.85E+01 | 1.25E-03 | 2.60E-02 | 9.56E+00 |  |
| Hsp90ab1 | 1.10E+03 | 1.25E-03 | 2.60E-02 | 1.55E+00 |  |
| 1700029B22Rik | 2.43E+02 | 1.27E-03 | 2.64E-02 | -2.48E+00 |  |
| 1700001O22Rik | 6.56E+02 | 1.27E-03 | 2.64E-02 | -1.99E+00 |  |
| Scarna8 | 4.60E+01 | 1.27E-03 | 2.65E-02 | 9.69E+00 |  |
| Gm26493 | 2.04E+02 | 1.27E-03 | 2.65E-02 | 1.95E+01 |  |
| Gm6313 | 4.57E+01 | 1.28E-03 | 2.66E-02 | -8.26E+00 |  |
| Gm15756 | 5.40E+02 | 1.28E-03 | 2.66E-02 | -1.99E+00 |  |
| Nudt7 | 3.55E+01 | 1.28E-03 | 2.66E-02 | -2.17E+01 |  |
| Mrpl48 | 2.50E+02 | 1.29E-03 | 2.66E-02 | -2.03E+00 |  |
| Pfn2 | 9.74E+02 | 1.29E-03 | 2.67E-02 | 1.63E+00 |  |
| Pdgfd | 2.47E+02 | 1.29E-03 | 2.67E-02 | -2.60E+00 |  |
| Zfp9 | 5.70E+01 | 1.29E-03 | 2.67E-02 | -7.73E+00 |  |
| Hist1h2be | 6.74E+01 | 1.30E-03 | 2.69E-02 | -5.22E+00 |  |
| Csnk2a2 | 9.46E+02 | 1.32E-03 | 2.73E-02 | 1.66E+00 |  |
| Ifih1 | 9.50E+01 | 1.32E-03 | 2.73E-02 | -4.94E+00 |  |
| Glns-ps1 | 9.98E+01 | 1.33E-03 | 2.74E-02 | -3.19E+00 |  |
| Wwc2 | 1.49E+03 | 1.34E-03 | 2.75E-02 | 1.51E+00 |  |
| 1700019M22Rik | 1.14E+03 | 1.34E-03 | 2.75E-02 | -1.60E+00 |  |
| Kcnb1 | 2.13E+02 | 1.34E-03 | 2.76E-02 | -2.43E+00 |  |
| Rnf148 | 5.13E+02 | 1.37E-03 | 2.81E-02 | -1.74E+00 |  |
| Gm20749 | 5.45E+01 | 1.38E-03 | 2.83E-02 | 1.06E+01 |  |
| Paip1 | 5.84E+02 | 1.40E-03 | 2.86E-02 | 1.92E+00 |  |
| Vmn2r79 | 1.44E+02 | 1.40E-03 | 2.86E-02 | -2.96E+00 |  |
| Lamb3 | 2.00E+02 | 1.40E-03 | 2.86E-02 | -2.70E+00 |  |
| Olfr1132 | 5.43E+01 | 1.40E-03 | 2.86E-02 | -6.70E+00 |  |
| Slc7a11 | 1.99E+02 | 1.40E-03 | 2.86E-02 | -3.70E+00 |  |
| Atm | 2.86E+02 | 1.40E-03 | 2.86E-02 | 2.33E+00 |  |
| Lrp2bp | 9.79E+02 | 1.40E-03 | 2.86E-02 | -1.62E+00 |  |
| Tmem210 | 3.96E+02 | 1.41E-03 | 2.86E-02 | -2.20E+00 |  |
| Gm26825 | 3.28E+02 | 1.41E-03 | 2.87E-02 | 4.09E+00 |  |
| Aunip | 9.53E+01 | 1.42E-03 | 2.87E-02 | -3.81E+00 |  |
| Gm45257 | 5.51E+01 | 1.42E-03 | 2.87E-02 | -7.66E+00 |  |
| Gm11422 | 4.58E+01 | 1.42E-03 | 2.88E-02 | 1.30E+01 |  |
| Trabd2b | 9.59E+01 | 1.43E-03 | 2.89E-02 | -9.21E+00 |  |
| Atf4 | 4.43E+02 | 1.43E-03 | 2.89E-02 | -1.91E+00 |  |
| Rex2 | 5.03E+02 | 1.44E-03 | 2.90E-02 | 2.74E+00 |  |
| Mbtps2 | 1.67E+02 | 1.45E-03 | 2.91E-02 | -4.09E+00 |  |
| Gm19434 | 3.31E+01 | 1.45E-03 | 2.92E-02 | -1.85E+01 |  |
| Rnu5g | 1.40E+04 | 1.46E-03 | 2.93E-02 | 1.43E+01 |  |
| Klhl10 | 2.76E+03 | 1.46E-03 | 2.94E-02 | -1.51E+00 |  |
| Snx27 | 1.55E+03 | 1.46E-03 | 2.94E-02 | 1.54E+00 |  |
| Rubcnl | 5.74E+01 | 1.46E-03 | 2.94E-02 | -9.88E+00 |  |
| Fndc8 | 5.25E+02 | 1.47E-03 | 2.94E-02 | -1.91E+00 |  |
| Plxnb1 | 1.97E+02 | 1.47E-03 | 2.94E-02 | 3.00E+00 |  |
| Pabpc4l | 7.87E+01 | 1.49E-03 | 2.97E-02 | -8.59E+00 |  |
| Gm20611 | 1.72E+02 | 1.50E-03 | 2.99E-02 | -2.91E+00 |  |
| Ccdc110 | 1.11E+03 | 1.50E-03 | 2.99E-02 | 1.50E+00 |  |
| Tgfb2 | 5.99E+01 | 1.50E-03 | 3.00E-02 | -6.16E+00 |  |
| Asna1 | 2.84E+02 | 1.51E-03 | 3.00E-02 | 2.10E+00 |  |
| 1700013G24Rik | 1.36E+03 | 1.51E-03 | 3.00E-02 | -1.66E+00 |  |
| Gm47919 | 3.24E+01 | 1.52E-03 | 3.02E-02 | -2.00E+01 |  |
| Olfr959 | 1.01E+02 | 1.52E-03 | 3.02E-02 | -4.32E+00 |  |
| Gm13387 | 4.40E+01 | 1.53E-03 | 3.04E-02 | -2.27E+01 |  |
| Naif1 | 9.68E+02 | 1.54E-03 | 3.05E-02 | -1.59E+00 |  |
| Gm5177 | 5.46E+01 | 1.54E-03 | 3.05E-02 | -6.62E+00 |  |
| 9330158H04Rik | 1.22E+02 | 1.54E-03 | 3.05E-02 | -3.15E+00 |  |
| Myo18b | 7.45E+02 | 1.54E-03 | 3.05E-02 | -1.67E+00 |  |
| Spatc1 | 4.11E+02 | 1.55E-03 | 3.06E-02 | -1.93E+00 |  |
| Gm30292 | 4.49E+01 | 1.56E-03 | 3.07E-02 | -1.40E+01 |  |
| 2610318N02Rik | 1.45E+03 | 1.56E-03 | 3.08E-02 | -1.65E+00 |  |
| Gm34821 | 2.72E+02 | 1.57E-03 | 3.08E-02 | -2.32E+00 |  |
| Cytl1 | 2.94E+02 | 1.58E-03 | 3.10E-02 | -3.39E+00 |  |
| 1700092M07Rik | 5.55E+02 | 1.58E-03 | 3.11E-02 | -1.73E+00 |  |
| 4930453L07Rik | 2.52E+02 | 1.59E-03 | 3.11E-02 | 2.25E+00 |  |
| Gm23925 | 2.41E+02 | 1.59E-03 | 3.11E-02 | 2.92E+00 |  |
| Itpk1 | 3.21E+02 | 1.59E-03 | 3.11E-02 | 2.13E+00 |  |
| Cnot1 | 6.51E+03 | 1.59E-03 | 3.11E-02 | 1.56E+00 |  |
| Vmn2r42 | 4.94E+01 | 1.59E-03 | 3.11E-02 | -9.27E+00 |  |
| C87436 | 9.26E+02 | 1.59E-03 | 3.11E-02 | -1.65E+00 |  |
| Epb42 | 5.21E+01 | 1.60E-03 | 3.12E-02 | -1.11E+01 |  |
| Olfr49 | 7.21E+01 | 1.61E-03 | 3.13E-02 | -5.63E+00 |  |
| Ttc30b | 4.51E+02 | 1.61E-03 | 3.13E-02 | -2.20E+00 |  |
| Wscd2 | 2.47E+02 | 1.61E-03 | 3.14E-02 | -2.03E+00 |  |
| Htr2a | 3.49E+01 | 1.62E-03 | 3.14E-02 | -1.41E+01 |  |
| Ybx1-ps2 | 5.05E+02 | 1.62E-03 | 3.14E-02 | 1.96E+00 |  |
| Olfr1157 | 3.96E+01 | 1.62E-03 | 3.15E-02 | -7.99E+00 |  |
| Rnf165 | 1.16E+02 | 1.62E-03 | 3.15E-02 | -3.44E+00 |  |
| Vmn2r96 | 7.28E+01 | 1.63E-03 | 3.16E-02 | -1.02E+01 |  |
| Gm26447 | 3.86E+03 | 1.63E-03 | 3.16E-02 | 3.20E+00 |  |
| Lancl2 | 1.79E+03 | 1.64E-03 | 3.16E-02 | 1.56E+00 |  |
| Catsper4 | 9.75E+02 | 1.64E-03 | 3.18E-02 | -1.73E+00 |  |
| 1700061N14Rik | 3.18E+02 | 1.65E-03 | 3.18E-02 | -2.35E+00 |  |
| Nek9 | 5.94E+02 | 1.66E-03 | 3.20E-02 | 1.92E+00 |  |
| Nptxr | 2.02E+02 | 1.66E-03 | 3.20E-02 | -2.46E+00 |  |
| Olfr1261 | 4.28E+01 | 1.66E-03 | 3.20E-02 | -1.99E+01 |  |
| Ccdc28a | 7.55E+02 | 1.67E-03 | 3.20E-02 | -2.03E+00 |  |
| Gm20658 | 4.13E+01 | 1.67E-03 | 3.20E-02 | -6.86E+00 |  |
| Gm27500 | 4.12E+01 | 1.68E-03 | 3.22E-02 | -6.55E+00 |  |
| Jakmip3 | 6.38E+01 | 1.68E-03 | 3.23E-02 | 1.09E+01 |  |
| Ccn1 | 4.60E+01 | 1.69E-03 | 3.23E-02 | -7.60E+00 |  |
| Mmp16 | 9.38E+01 | 1.70E-03 | 3.25E-02 | -4.29E+00 |  |
| Gm22154 | 1.90E+02 | 1.70E-03 | 3.25E-02 | 2.61E+00 |  |
| Gm47036 | 3.12E+02 | 1.71E-03 | 3.27E-02 | -2.34E+00 |  |
| Rhov | 2.60E+02 | 1.72E-03 | 3.28E-02 | -2.15E+00 |  |
| Gm13723 | 1.23E+02 | 1.72E-03 | 3.29E-02 | -3.20E+00 |  |
| Gm42785 | 1.55E+02 | 1.74E-03 | 3.30E-02 | -3.53E+00 |  |
| Plppr4 | 7.76E+01 | 1.74E-03 | 3.30E-02 | -5.61E+00 |  |
| Olfr1204 | 1.04E+02 | 1.74E-03 | 3.30E-02 | -5.39E+00 |  |
| Vmn2r70 | 1.88E+02 | 1.74E-03 | 3.30E-02 | -3.25E+00 |  |
| Gm22270 | 4.08E+02 | 1.74E-03 | 3.31E-02 | 2.85E+00 |  |
| Gm49238 | 1.15E+02 | 1.74E-03 | 3.31E-02 | -3.59E+00 |  |
| Gm25989 | 1.82E+02 | 1.75E-03 | 3.32E-02 | 3.64E+00 |  |
| 4932411K12Rik | 1.09E+03 | 1.75E-03 | 3.32E-02 | -1.72E+00 |  |
| Gm37180 | 5.14E+01 | 1.76E-03 | 3.34E-02 | -8.02E+00 |  |
| Tmem156 | 2.38E+02 | 1.77E-03 | 3.36E-02 | -2.96E+00 |  |
| Rcor3 | 1.19E+03 | 1.78E-03 | 3.36E-02 | 1.56E+00 |  |
| Gm13369 | 8.07E+01 | 1.78E-03 | 3.36E-02 | -3.64E+00 |  |
| Obox3 | 6.45E+01 | 1.78E-03 | 3.37E-02 | -1.44E+01 |  |
| Tusc2 | 2.46E+02 | 1.79E-03 | 3.37E-02 | -2.24E+00 |  |
| Rnu11 | 6.38E+03 | 1.80E-03 | 3.39E-02 | 5.41E+00 |  |
| Gm42583 | 1.27E+02 | 1.81E-03 | 3.41E-02 | -3.12E+00 |  |
| Gm30211 | 3.31E+01 | 1.82E-03 | 3.41E-02 | -1.47E+01 |  |
| Gm18248 | 3.68E+01 | 1.82E-03 | 3.41E-02 | -3.91E+01 |  |
| Cacnb1 | 5.92E+02 | 1.83E-03 | 3.43E-02 | 1.75E+00 |  |
| Gm36325 | 3.55E+01 | 1.83E-03 | 3.44E-02 | -3.78E+01 |  |
| Tbx5 | 1.15E+02 | 1.83E-03 | 3.44E-02 | -3.65E+00 |  |
| Slc17a3 | 1.01E+02 | 1.84E-03 | 3.44E-02 | -6.50E+00 |  |
| Zc2hc1b | 6.40E+01 | 1.84E-03 | 3.44E-02 | -5.06E+00 |  |
| Ptchd4 | 4.39E+01 | 1.85E-03 | 3.44E-02 | -6.60E+00 |  |
| Fam229a | 4.82E+02 | 1.85E-03 | 3.45E-02 | -1.67E+00 |  |
| Vmn1r77 | 2.13E+02 | 1.86E-03 | 3.45E-02 | -3.40E+00 |  |
| 1700049E17Rik2 | 1.51E+02 | 1.86E-03 | 3.45E-02 | -3.06E+00 |  |
| Snord57 | 1.12E+02 | 1.86E-03 | 3.45E-02 | 4.77E+00 |  |
| Fcrl6 | 5.53E+01 | 1.86E-03 | 3.45E-02 | -6.08E+00 |  |
| Samd11 | 6.80E+01 | 1.86E-03 | 3.46E-02 | 5.70E+00 |  |
| Nalcn | 1.78E+02 | 1.87E-03 | 3.46E-02 | -3.20E+00 |  |
| Myoz3 | 5.95E+01 | 1.88E-03 | 3.47E-02 | -1.76E+01 |  |
| Gm17473 | 4.58E+01 | 1.88E-03 | 3.47E-02 | -9.55E+00 |  |
| Eif4e | 1.04E+03 | 1.88E-03 | 3.47E-02 | -1.56E+00 |  |
| Gm24523 | 7.69E+02 | 1.89E-03 | 3.48E-02 | 2.48E+00 |  |
| Tarsl2 | 3.14E+02 | 1.89E-03 | 3.48E-02 | 2.44E+00 |  |
| Eif3m | 2.36E+02 | 1.90E-03 | 3.50E-02 | 2.26E+00 |  |
| Kcnu1 | 1.30E+03 | 1.91E-03 | 3.52E-02 | -1.76E+00 |  |
| Zfp987 | 6.95E+01 | 1.92E-03 | 3.53E-02 | -1.40E+01 |  |
| 4933402C06Rik | 2.53E+02 | 1.93E-03 | 3.54E-02 | -2.67E+00 |  |
| Rtkn2 | 6.83E+01 | 1.93E-03 | 3.54E-02 | -8.05E+00 |  |
| Gnpda2 | 1.08E+03 | 1.93E-03 | 3.54E-02 | -1.74E+00 |  |
| Tent5b | 1.68E+02 | 1.94E-03 | 3.54E-02 | 3.18E+00 |  |
| Runx1 | 1.37E+02 | 1.94E-03 | 3.54E-02 | -4.50E+00 |  |
| Gm47161 | 4.05E+01 | 1.94E-03 | 3.54E-02 | -1.00E+01 |  |
| Mrip-ps | 4.55E+02 | 1.94E-03 | 3.54E-02 | 1.90E+00 |  |
| Olfr1454 | 7.84E+01 | 1.94E-03 | 3.55E-02 | -4.68E+00 |  |
| Gm13270 | 6.03E+01 | 1.95E-03 | 3.56E-02 | -7.14E+00 |  |
| Tas1r1 | 2.60E+02 | 1.95E-03 | 3.56E-02 | -2.93E+00 |  |
| Atp8b5 | 1.08E+03 | 1.96E-03 | 3.57E-02 | 1.51E+00 |  |
| Vmn1r20 | 5.22E+01 | 1.97E-03 | 3.59E-02 | -5.82E+00 |  |
| Spanxn4 | 6.85E+02 | 1.97E-03 | 3.59E-02 | -1.71E+00 |  |
| Gm20337 | 2.11E+02 | 1.98E-03 | 3.59E-02 | -2.52E+00 |  |
| 1700048F04Rik | 1.95E+02 | 1.99E-03 | 3.61E-02 | -2.66E+00 |  |
| Gabarapl1 | 7.78E+02 | 1.99E-03 | 3.61E-02 | -1.56E+00 |  |
| Adipoq | 1.89E+02 | 1.99E-03 | 3.61E-02 | -2.58E+00 |  |
| Smarcc2 | 1.83E+03 | 2.00E-03 | 3.62E-02 | 1.58E+00 |  |
| Prokr1 | 4.73E+01 | 2.00E-03 | 3.62E-02 | -8.66E+00 |  |
| Gm17078 | 3.42E+01 | 2.01E-03 | 3.63E-02 | -1.95E+01 |  |
| Clic4 | 7.41E+02 | 2.01E-03 | 3.63E-02 | 1.75E+00 |  |
| Gm8108 | 2.95E+01 | 2.02E-03 | 3.64E-02 | 1.99E+01 |  |
| Gm15681 | 6.49E+01 | 2.02E-03 | 3.64E-02 | -5.64E+00 |  |
| Ctdp1 | 5.86E+02 | 2.02E-03 | 3.64E-02 | -1.79E+00 |  |
| Hsfy2 | 1.12E+03 | 2.02E-03 | 3.64E-02 | -1.84E+00 |  |
| Oaz1 | 3.56E+02 | 2.03E-03 | 3.65E-02 | -2.08E+00 |  |
| Olfr350 | 4.87E+01 | 2.04E-03 | 3.67E-02 | -2.44E+01 |  |
| 1700003F12Rik | 1.28E+03 | 2.05E-03 | 3.67E-02 | -1.76E+00 |  |
| Lrrc41 | 9.68E+02 | 2.05E-03 | 3.68E-02 | 1.53E+00 |  |
| Trim30b | 7.17E+01 | 2.05E-03 | 3.68E-02 | -6.05E+00 |  |
| Olfr402 | 1.18E+02 | 2.06E-03 | 3.69E-02 | -6.37E+00 |  |
| Snord13 | 7.32E+03 | 2.07E-03 | 3.69E-02 | 1.60E+00 |  |
| Olfr495 | 3.52E+01 | 2.07E-03 | 3.69E-02 | -1.12E+01 |  |
| Kcnh4 | 6.24E+01 | 2.07E-03 | 3.69E-02 | 7.26E+00 |  |
| Aga | 5.72E+01 | 2.07E-03 | 3.70E-02 | -6.56E+00 |  |
| Gm28437 | 6.63E+03 | 2.07E-03 | 3.70E-02 | 1.68E+00 |  |
| Vmn2r44 | 8.45E+01 | 2.08E-03 | 3.70E-02 | -1.19E+01 |  |
| Snora44 | 2.98E+02 | 2.08E-03 | 3.70E-02 | 2.34E+00 |  |
| Inpp5k | 8.71E+02 | 2.08E-03 | 3.70E-02 | 1.71E+00 |  |
| P4ha2 | 2.54E+02 | 2.08E-03 | 3.70E-02 | -2.13E+00 |  |
| Myo7b | 5.02E+01 | 2.08E-03 | 3.70E-02 | -7.25E+00 |  |
| Gm5512 | 4.84E+02 | 2.10E-03 | 3.72E-02 | -1.76E+00 |  |
| Sis | 4.26E+01 | 2.10E-03 | 3.72E-02 | -1.68E+01 |  |
| Olfr896-ps1 | 1.12E+02 | 2.10E-03 | 3.72E-02 | -3.45E+00 |  |
| Vwa3a | 9.46E+02 | 2.10E-03 | 3.72E-02 | -1.58E+00 |  |
| Gm1988 | 6.16E+02 | 2.11E-03 | 3.72E-02 | -2.24E+00 |  |
| Plcg1 | 3.60E+02 | 2.12E-03 | 3.73E-02 | 2.04E+00 |  |
| Zfp93 | 1.25E+02 | 2.12E-03 | 3.74E-02 | -3.55E+00 |  |
| Gm42544 | 6.27E+01 | 2.12E-03 | 3.74E-02 | -6.87E+00 |  |
| Gm49618 | 1.02E+02 | 2.12E-03 | 3.74E-02 | -3.67E+00 |  |
| Rnf214 | 5.98E+02 | 2.12E-03 | 3.74E-02 | 1.69E+00 |  |
| Gm49345 | 3.30E+01 | 2.13E-03 | 3.74E-02 | 2.24E+01 |  |
| Rab3gap2 | 3.16E+02 | 2.13E-03 | 3.74E-02 | 2.06E+00 |  |
| Olfr353 | 5.37E+01 | 2.13E-03 | 3.74E-02 | -1.30E+01 |  |
| A530053G22Rik | 1.04E+02 | 2.13E-03 | 3.74E-02 | -4.74E+00 |  |
| A930002C04Rik | 1.16E+02 | 2.13E-03 | 3.74E-02 | 3.62E+00 |  |
| Apip | 9.56E+01 | 2.14E-03 | 3.74E-02 | 4.06E+00 |  |
| Shc3 | 3.07E+01 | 2.14E-03 | 3.75E-02 | -1.95E+01 |  |
| Ankrd61 | 1.12E+03 | 2.15E-03 | 3.75E-02 | -1.65E+00 |  |
| Vmn2r102 | 2.23E+02 | 2.15E-03 | 3.75E-02 | -3.04E+00 |  |
| Tipin | 6.40E+02 | 2.15E-03 | 3.75E-02 | -1.96E+00 |  |
| Lpar1 | 7.59E+02 | 2.15E-03 | 3.76E-02 | -1.67E+00 |  |
| Aknad1 | 3.10E+02 | 2.16E-03 | 3.76E-02 | -1.88E+00 |  |
| Npas4 | 1.63E+02 | 2.16E-03 | 3.76E-02 | -2.48E+00 |  |
| 1700063H06Rik | 3.92E+01 | 2.16E-03 | 3.76E-02 | -1.01E+01 |  |
| A730009L09Rik | 3.57E+01 | 2.16E-03 | 3.76E-02 | -1.64E+01 |  |
| Thoc5 | 5.35E+02 | 2.17E-03 | 3.76E-02 | 2.01E+00 |  |
| Gm38233 | 3.35E+01 | 2.17E-03 | 3.76E-02 | 1.47E+01 |  |
| Gm49087 | 5.55E+01 | 2.18E-03 | 3.77E-02 | -7.14E+00 |  |
| Iqcm | 1.15E+03 | 2.18E-03 | 3.77E-02 | -1.61E+00 |  |
| Bfar | 4.30E+02 | 2.19E-03 | 3.79E-02 | -1.96E+00 |  |
| Slc13a2os | 3.06E+01 | 2.20E-03 | 3.80E-02 | -1.08E+01 |  |
| Gm47689 | 2.87E+01 | 2.20E-03 | 3.80E-02 | -3.69E+01 |  |
| Olfr911-ps1 | 7.56E+01 | 2.21E-03 | 3.81E-02 | -5.16E+00 |  |
| Gm37498 | 7.76E+01 | 2.21E-03 | 3.81E-02 | -1.12E+01 |  |
| Olfr1093 | 4.50E+01 | 2.21E-03 | 3.81E-02 | -8.99E+00 |  |
| Gm7664 | 3.14E+02 | 2.22E-03 | 3.82E-02 | -3.13E+00 |  |
| Lrrc40 | 4.30E+02 | 2.22E-03 | 3.82E-02 | -1.87E+00 |  |
| Gm16198 | 5.00E+01 | 2.22E-03 | 3.82E-02 | -6.36E+00 |  |
| Ryr2 | 1.46E+02 | 2.23E-03 | 3.84E-02 | -3.44E+00 |  |
| Zfp239 | 1.24E+02 | 2.24E-03 | 3.84E-02 | -3.26E+00 |  |
| Gm37775 | 6.13E+01 | 2.24E-03 | 3.84E-02 | -1.55E+01 |  |
| Gm17167 | 9.21E+02 | 2.24E-03 | 3.84E-02 | -1.82E+00 |  |
| 4930449C09Rik | 1.43E+03 | 2.25E-03 | 3.85E-02 | 1.70E+00 |  |
| Vmn2r65 | 1.31E+02 | 2.25E-03 | 3.85E-02 | -4.67E+00 |  |
| Rgsl1 | 5.83E+02 | 2.25E-03 | 3.86E-02 | -1.73E+00 |  |
| Gm30083 | 5.32E+02 | 2.28E-03 | 3.89E-02 | -2.09E+00 |  |
| Gm44542 | 5.00E+01 | 2.28E-03 | 3.89E-02 | -1.16E+01 |  |
| Gga3 | 5.05E+02 | 2.28E-03 | 3.89E-02 | -1.78E+00 |  |
| Exosc10 | 7.17E+02 | 2.29E-03 | 3.90E-02 | 1.81E+00 |  |
| AC140980.1 | 2.79E+01 | 2.29E-03 | 3.90E-02 | -2.05E+01 |  |
| BC049739 | 1.64E+02 | 2.29E-03 | 3.90E-02 | -3.55E+00 |  |
| Gm7972 | 8.86E+01 | 2.29E-03 | 3.90E-02 | 5.60E+00 |  |
| Nim1k | 2.76E+02 | 2.30E-03 | 3.90E-02 | -1.93E+00 |  |
| Gm47494 | 6.32E+01 | 2.30E-03 | 3.91E-02 | -7.61E+00 |  |
| 1700022A22Rik | 4.67E+02 | 2.30E-03 | 3.91E-02 | -1.66E+00 |  |
| Gm44502 | 4.20E+01 | 2.31E-03 | 3.93E-02 | 2.84E+01 |  |
| Trpd52l3 | 7.94E+02 | 2.32E-03 | 3.93E-02 | 1.74E+00 |  |
| Cacna2d1 | 4.18E+02 | 2.32E-03 | 3.93E-02 | -1.70E+00 |  |
| Gm6377 | 3.99E+01 | 2.36E-03 | 3.99E-02 | -8.52E+00 |  |
| Gm8369 | 1.48E+02 | 2.37E-03 | 4.00E-02 | -2.80E+00 |  |
| Olfr134 | 2.71E+01 | 2.37E-03 | 4.00E-02 | -2.82E+01 |  |
| Catsperd | 1.47E+03 | 2.37E-03 | 4.00E-02 | 1.52E+00 |  |
| Gm11946 | 4.43E+01 | 2.37E-03 | 4.00E-02 | -6.40E+00 |  |
| Gm19683 | 3.57E+01 | 2.39E-03 | 4.01E-02 | -2.64E+01 |  |
| Snora33 | 3.85E+02 | 2.39E-03 | 4.01E-02 | 3.15E+00 |  |
| Gm23297 | 8.77E+02 | 2.39E-03 | 4.01E-02 | 1.96E+00 |  |
| Ugt2b5 | 3.47E+01 | 2.39E-03 | 4.01E-02 | -1.47E+01 |  |
| Gm48798 | 2.98E+01 | 2.39E-03 | 4.01E-02 | -1.93E+01 |  |
| Gm48401 | 9.79E+01 | 2.39E-03 | 4.01E-02 | -3.16E+00 |  |
| Adarb2 | 1.89E+02 | 2.40E-03 | 4.01E-02 | -3.59E+00 |  |
| Ccdc184 | 6.10E+02 | 2.40E-03 | 4.01E-02 | -1.83E+00 |  |
| Gm38012 | 5.41E+01 | 2.40E-03 | 4.01E-02 | -7.56E+00 |  |
| Nhlh2 | 3.37E+01 | 2.40E-03 | 4.01E-02 | -1.00E+01 |  |
| Gm16433 | 1.82E+02 | 2.40E-03 | 4.01E-02 | 3.09E+00 |  |
| Gm31822 | 4.35E+01 | 2.40E-03 | 4.01E-02 | -1.03E+01 |  |
| Fth1 | 3.16E+02 | 2.42E-03 | 4.04E-02 | 2.18E+00 |  |
| Tnnt2 | 3.63E+01 | 2.42E-03 | 4.04E-02 | -1.20E+01 |  |
| Rnaseh2a | 1.80E+03 | 2.44E-03 | 4.06E-02 | 1.57E+00 |  |
| Ipo4 | 9.28E+02 | 2.44E-03 | 4.07E-02 | 1.56E+00 |  |
| Plin2 | 4.15E+02 | 2.45E-03 | 4.07E-02 | -1.93E+00 |  |
| Spdya | 1.33E+03 | 2.45E-03 | 4.07E-02 | 1.69E+00 |  |
| Gm41504 | 2.98E+01 | 2.46E-03 | 4.08E-02 | -2.19E+01 |  |
| Grip1 | 1.02E+03 | 2.46E-03 | 4.08E-02 | -1.57E+00 |  |
| Gm17200 | 1.25E+02 | 2.46E-03 | 4.08E-02 | 4.18E+00 |  |
| Gm38382 | 5.94E+01 | 2.46E-03 | 4.08E-02 | 7.77E+00 |  |
| Ptprc | 1.61E+02 | 2.47E-03 | 4.08E-02 | -2.68E+00 |  |
| Gm45768 | 8.15E+01 | 2.48E-03 | 4.10E-02 | -3.41E+00 |  |
| Golga7b | 2.29E+02 | 2.49E-03 | 4.12E-02 | -2.11E+00 |  |
| Syce2 | 5.94E+02 | 2.50E-03 | 4.14E-02 | -1.71E+00 |  |
| Cdv3-ps | 5.86E+02 | 2.51E-03 | 4.14E-02 | -1.74E+00 |  |
| Tssk2 | 1.81E+03 | 2.51E-03 | 4.14E-02 | -1.53E+00 |  |
| Crat | 4.42E+03 | 2.51E-03 | 4.14E-02 | 1.51E+00 |  |
| Irgc1 | 2.65E+03 | 2.52E-03 | 4.15E-02 | -1.51E+00 |  |
| Cabp4 | 5.25E+01 | 2.52E-03 | 4.15E-02 | -5.65E+00 |  |
| Dock3 | 1.05E+03 | 2.53E-03 | 4.16E-02 | 1.57E+00 |  |
| Chrm2 | 9.62E+01 | 2.53E-03 | 4.16E-02 | -4.28E+00 |  |
| Grip1os2 | 3.18E+01 | 2.54E-03 | 4.17E-02 | -9.12E+00 |  |
| 4933427I04Rik | 7.83E+02 | 2.54E-03 | 4.17E-02 | -1.69E+00 |  |
| Gm6472 | 7.98E+02 | 2.55E-03 | 4.17E-02 | 1.60E+00 |  |
| Gm6374 | 8.12E+01 | 2.55E-03 | 4.17E-02 | -3.94E+00 |  |
| Cnbd1 | 4.30E+02 | 2.55E-03 | 4.17E-02 | -1.85E+00 |  |
| Gm25395 | 1.20E+03 | 2.55E-03 | 4.17E-02 | 1.95E+00 |  |
| Gm11585 | 8.50E+01 | 2.55E-03 | 4.17E-02 | 4.10E+00 |  |
| Ube2i | 7.07E+02 | 2.56E-03 | 4.18E-02 | 1.61E+00 |  |
| H2al1m | 7.35E+01 | 2.57E-03 | 4.18E-02 | 1.27E+01 |  |
| Vmn2r14 | 9.57E+01 | 2.58E-03 | 4.20E-02 | -5.26E+00 |  |
| Gm6176 | 4.13E+01 | 2.58E-03 | 4.20E-02 | -2.17E+01 |  |
| Bag1 | 9.11E+02 | 2.59E-03 | 4.21E-02 | -1.55E+00 |  |
| 4930512P04Rik | 4.04E+01 | 2.59E-03 | 4.21E-02 | 1.12E+01 |  |
| Gm47047 | 1.09E+02 | 2.61E-03 | 4.23E-02 | -3.27E+00 |  |
| Gm6740 | 3.04E+01 | 2.61E-03 | 4.23E-02 | -2.66E+01 |  |
| Gm43942 | 3.51E+01 | 2.61E-03 | 4.23E-02 | -1.15E+01 |  |
| Olfr250 | 5.64E+01 | 2.62E-03 | 4.24E-02 | -1.11E+01 |  |
| Gm20356 | 6.55E+01 | 2.62E-03 | 4.24E-02 | -8.26E+00 |  |
| Vmn1r219 | 9.17E+01 | 2.63E-03 | 4.25E-02 | -7.71E+00 |  |
| Olfr167 | 1.08E+02 | 2.63E-03 | 4.26E-02 | -5.30E+00 |  |
| Drc3 | 6.37E+02 | 2.64E-03 | 4.26E-02 | 1.65E+00 |  |
| Sel1l2 | 1.11E+03 | 2.65E-03 | 4.28E-02 | -1.63E+00 |  |
| Bicral | 6.15E+02 | 2.66E-03 | 4.28E-02 | -1.66E+00 |  |
| Nedd4 | 4.07E+02 | 2.66E-03 | 4.28E-02 | 1.98E+00 |  |
| Cnot9 | 7.59E+02 | 2.67E-03 | 4.29E-02 | 1.51E+00 |  |
| Ifrd1 | 4.32E+02 | 2.67E-03 | 4.29E-02 | -1.94E+00 |  |
| Gm16506 | 9.08E+01 | 2.67E-03 | 4.30E-02 | -7.06E+00 |  |
| Olfr943 | 8.11E+01 | 2.69E-03 | 4.32E-02 | -4.87E+00 |  |
| Gm13456 | 1.31E+03 | 2.70E-03 | 4.32E-02 | 1.63E+00 |  |
| Nubp2 | 6.41E+02 | 2.72E-03 | 4.36E-02 | 1.63E+00 |  |
| Gm43781 | 5.54E+01 | 2.73E-03 | 4.37E-02 | -9.46E+00 |  |
| Gm3636 | 1.69E+02 | 2.74E-03 | 4.39E-02 | -2.36E+00 |  |
| Camk4 | 3.73E+02 | 2.76E-03 | 4.41E-02 | -1.98E+00 |  |
| Olfr609 | 4.29E+01 | 2.76E-03 | 4.41E-02 | -1.51E+01 |  |
| Insl3 | 1.06E+02 | 2.76E-03 | 4.41E-02 | 4.23E+00 |  |
| Tbata | 1.52E+03 | 2.76E-03 | 4.41E-02 | -1.60E+00 |  |
| Gsk3b | 1.46E+03 | 2.77E-03 | 4.41E-02 | -1.53E+00 |  |
| Kcnk9 | 1.49E+02 | 2.78E-03 | 4.42E-02 | -4.70E+00 |  |
| Gjc3 | 3.73E+01 | 2.78E-03 | 4.43E-02 | -1.16E+01 |  |
| Zfp979 | 6.64E+02 | 2.79E-03 | 4.43E-02 | -2.07E+00 |  |
| Vmn2r75 | 8.07E+01 | 2.80E-03 | 4.44E-02 | -6.16E+00 |  |
| Gm10635 | 3.27E+01 | 2.81E-03 | 4.45E-02 | -1.09E+01 |  |
| Gm18246 | 3.11E+01 | 2.81E-03 | 4.46E-02 | -2.39E+01 |  |
| Csnk2b | 2.34E+03 | 2.82E-03 | 4.46E-02 | 1.57E+00 |  |
| Vdac3 | 6.26E+02 | 2.82E-03 | 4.46E-02 | 1.73E+00 |  |
| Wwp2 | 6.28E+02 | 2.83E-03 | 4.47E-02 | -1.82E+00 |  |
| Gm9239 | 7.82E+02 | 2.83E-03 | 4.47E-02 | -1.85E+00 |  |
| Gm25777 | 1.17E+02 | 2.83E-03 | 4.47E-02 | 4.36E+00 |  |
| Sgsm3 | 2.90E+02 | 2.84E-03 | 4.48E-02 | 2.02E+00 |  |
| Chchd6 | 6.39E+02 | 2.84E-03 | 4.49E-02 | 1.80E+00 |  |
| Gm43011 | 8.30E+01 | 2.85E-03 | 4.49E-02 | -3.71E+00 |  |
| Gm48231 | 1.24E+02 | 2.85E-03 | 4.50E-02 | -3.94E+00 |  |
| Gm5913 | 3.30E+02 | 2.86E-03 | 4.51E-02 | -1.98E+00 |  |
| Speer9-ps1 | 2.33E+02 | 2.87E-03 | 4.52E-02 | -2.31E+00 |  |
| Gm14287 | 3.00E+01 | 2.88E-03 | 4.52E-02 | -2.78E+01 |  |
| Dchs2 | 6.81E+01 | 2.89E-03 | 4.54E-02 | -7.03E+00 |  |
| Psmb11 | 7.08E+01 | 2.89E-03 | 4.54E-02 | -6.39E+00 |  |
| Prdm16 | 1.91E+02 | 2.89E-03 | 4.54E-02 | -2.88E+00 |  |
| Siglecg | 5.50E+01 | 2.91E-03 | 4.56E-02 | -5.25E+00 |  |
| Pacs1 | 1.75E+03 | 2.91E-03 | 4.56E-02 | 1.54E+00 |  |
| Nras | 9.81E+02 | 2.91E-03 | 4.56E-02 | 1.55E+00 |  |
| Gm50092 | 1.15E+03 | 2.92E-03 | 4.58E-02 | -1.56E+00 |  |
| Tex50 | 5.54E+02 | 2.93E-03 | 4.58E-02 | -1.66E+00 |  |
| Atp11a | 7.50E+02 | 2.95E-03 | 4.62E-02 | 1.61E+00 |  |
| Clp1 | 5.44E+02 | 2.96E-03 | 4.62E-02 | -1.76E+00 |  |
| Pdcd5 | 2.95E+02 | 2.97E-03 | 4.63E-02 | 1.89E+00 |  |
| Gm14062 | 6.04E+01 | 2.97E-03 | 4.63E-02 | -8.36E+00 |  |
| Dll3 | 9.74E+01 | 2.98E-03 | 4.63E-02 | 3.86E+00 |  |
| Ric1 | 3.59E+02 | 2.98E-03 | 4.63E-02 | 1.95E+00 |  |
| Oser1 | 1.26E+03 | 3.00E-03 | 4.66E-02 | -1.62E+00 |  |
| 4930433I11Rik | 8.80E+02 | 3.01E-03 | 4.67E-02 | -1.63E+00 |  |
| Gm15577 | 7.04E+01 | 3.01E-03 | 4.67E-02 | -1.22E+01 |  |
| Gpr45 | 6.57E+01 | 3.02E-03 | 4.69E-02 | -1.51E+01 |  |
| Scarna17 | 6.50E+01 | 3.06E-03 | 4.75E-02 | 6.80E+00 |  |
| Gm3327 | 1.62E+02 | 3.07E-03 | 4.75E-02 | -2.97E+00 |  |
| Gm30655 | 7.20E+01 | 3.07E-03 | 4.75E-02 | -6.97E+00 |  |
| D930019O06Rik | 7.02E+01 | 3.07E-03 | 4.75E-02 | -5.73E+00 |  |
| Donson | 6.34E+02 | 3.08E-03 | 4.75E-02 | 1.70E+00 |  |
| Olfr1418 | 4.56E+01 | 3.08E-03 | 4.76E-02 | -9.47E+00 |  |
| Dlgap4 | 3.78E+02 | 3.08E-03 | 4.76E-02 | -1.79E+00 |  |
| Vmn2r-ps130 | 2.11E+02 | 3.09E-03 | 4.76E-02 | -3.44E+00 |  |
| Atxn10 | 9.20E+02 | 3.09E-03 | 4.77E-02 | 1.56E+00 |  |
| Paqr8 | 5.92E+02 | 3.10E-03 | 4.77E-02 | 1.67E+00 |  |
| Gm20878 | 4.87E+02 | 3.13E-03 | 4.81E-02 | -2.31E+00 |  |
| Olfr924 | 1.64E+02 | 3.13E-03 | 4.81E-02 | -2.98E+00 |  |
| Snord1b | 5.63E+01 | 3.14E-03 | 4.82E-02 | 1.63E+01 |  |
| Unk | 1.76E+02 | 3.15E-03 | 4.82E-02 | 2.29E+00 |  |
| Gm15638 | 6.19E+01 | 3.15E-03 | 4.82E-02 | -6.24E+00 |  |
| 4933432I09Rik | 3.80E+02 | 3.15E-03 | 4.82E-02 | -1.86E+00 |  |
| Gm3200 | 3.46E+01 | 3.16E-03 | 4.84E-02 | 2.30E+01 |  |
| Bhmt | 8.04E+01 | 3.17E-03 | 4.84E-02 | 6.60E+00 |  |
| Akap6 | 5.28E+02 | 3.17E-03 | 4.84E-02 | -2.13E+00 |  |
| Ulbp1 | 1.48E+02 | 3.18E-03 | 4.85E-02 | -2.91E+00 |  |
| Sox30 | 1.72E+03 | 3.18E-03 | 4.85E-02 | -1.51E+00 |  |
| Sv2a | 1.08E+02 | 3.18E-03 | 4.85E-02 | -4.17E+00 |  |
| Nebl | 1.10E+02 | 3.19E-03 | 4.85E-02 | -3.83E+00 |  |
| Ndrg3 | 1.69E+03 | 3.19E-03 | 4.85E-02 | 1.53E+00 |  |
| 4930428D20Rik | 9.38E+01 | 3.19E-03 | 4.85E-02 | -3.09E+00 |  |
| Gm14900 | 2.77E+01 | 3.21E-03 | 4.87E-02 | -3.02E+01 |  |
| Plekhg1 | 2.15E+02 | 3.21E-03 | 4.87E-02 | -2.11E+00 |  |
| Prrx1 | 1.46E+02 | 3.21E-03 | 4.87E-02 | -2.92E+00 |  |
| Gm43200 | 1.97E+02 | 3.24E-03 | 4.91E-02 | 2.31E+00 |  |
| Gm47700 | 3.75E+01 | 3.25E-03 | 4.91E-02 | -6.27E+00 |  |
| Izumo3 | 1.05E+03 | 3.25E-03 | 4.92E-02 | -1.51E+00 |  |
| Gm16072 | 6.24E+01 | 3.26E-03 | 4.93E-02 | -7.04E+00 |  |
| Acly | 2.04E+02 | 3.27E-03 | 4.94E-02 | 2.37E+00 |  |
| Gm11960 | 1.29E+02 | 3.28E-03 | 4.96E-02 | 2.95E+00 |  |
| Snora21 | 4.11E+02 | 3.29E-03 | 4.96E-02 | 3.02E+00 |  |
| Gm16499 | 8.84E+01 | 3.29E-03 | 4.96E-02 | -4.09E+00 |  |
| Gm2824 | 1.59E+02 | 3.30E-03 | 4.97E-02 | 3.21E+00 |  |
| Gm2396 | 4.48E+03 | 3.32E-03 | 4.98E-02 | 1.60E+00 |  |
| Csk | 6.84E+01 | 3.32E-03 | 4.98E-02 | 4.62E+00 |  |
| Rab11a | 3.81E+02 | 3.32E-03 | 4.98E-02 | 1.74E+00 |  |
| Mrps12 | 2.68E+02 | 3.32E-03 | 4.98E-02 | -2.42E+00 |  |
| Pkn2 | 1.05E+03 | 3.33E-03 | 4.99E-02 | 1.51E+00 |  |
| Pip4k2a | 5.53E+01 | 3.33E-03 | 4.99E-02 | -5.16E+00 |  |
