# Supplementary material for: Role of Phosphorylated Gonadotropin-Regulated Testicular RNA Helicase (GRTH/DDX25) in the Regulation of Germ Cell Specific mRNAs in Chromatoid Bodies During Spermatogenesis
Source: Front Cell Dev Biol. 2020 Dec 23;8:580019. doi: 10.3389/fcell.2020.580019 (PMC7786181; doi:10.3389/fcell.2020.580019)
Supplement: Supplementary Table 1 — List of primers used for genotyping and validation of differentially enriched transcripts using qRT-PCR. [file Data_Sheet_1.zip › Supplementary files/Supplementary Table 7.docx]

**Supplementary Table 7:** Gene Ontology (GO) enrichment analysis showing functional category, biological process (BP), cellular component (CC) and molecular function (MF) with group ID (Term), category description, LogP and a list of gene symbols.

| **Category** | **Term** | **Description** | **LogP** | **Gene symbols** |
| --- | --- | --- | --- | --- |
| GO BP | GO:0048232 | male gamete generation | 15.97375 | Ace,Atm,Cftr,Crkl,Bscl2,Htt,Hspa1l,Hspa2,Odf1,Cdk16,Prm1,Prm2,Prm3,Qk,Tle3,Tnp1,Tnp2,Tssk2,Ubb,Zfp37,Spo11,Ddx25,Spag6l,Prdx4,Oaz3,Nphp1,Phc2,Sfmbt1,Fscn3,Ybx3,Pmfbp1,Tssk3,Suv39h2,Tbata,Klhl10,Wdr48,Paip2,H1fnt,Spata16,Spdya,Cabs1,Cabyr,Calr3,Spata18,1110017D15Rik,Tbc1d21,Spem1,Boll,Ropn1,Tssk6,Mael,Bbs4,Catsperd,Tsga10,Txndc2,Sox30,Spata20,Slc26a8,Catsper1,Txnrd3,Tmf1, Tbpl1,Arid4a,Rimbp3,Dpy19l2,Spata32,Acsbg2,Catsper4,Hfm1,Zfp541 |
| GO BP | GO:0007283 | spermatogenesis | 14.56357 | Ace,Cftr,Crkl,Bscl2,Htt,Hspa1l,Hspa2,Odf1,Cdk16,Prm1,Prm2,Prm3,Qk,Tle3,Tnp1,Tnp2,Tssk2,Zfp37,Spo11,Ddx25,Spag6l,Prdx4,Oaz3,Nphp1,Phc2,Sfmbt1,Fscn3,Ybx3,Pmfbp1,Tssk3,Tbata,Klhl10,Wdr48,Paip2,H1fnt,Spata16,Cabs1,Cabyr,Calr3,Spata18,1110017D15Rik,Tbc1d21,Spem1,Boll,Ropn1,Tssk6,Mael,Bbs4,Catsperd,Tsga10,Txndc2,Sox30,Spata20,Slc26a8,Catsper1,Txnrd3,Tmf1,Tbpl1,Arid4a,Rimbp3, Dpy19l2,Spata32,Acsbg2,Catsper4,Hfm1,Zfp541 |
| GO BP | GO:0048515 | spermatid differentiation | 8.11703 | Cftr,Bscl2,Hspa2,Prm1,Prm2,Qk,Tnp1,Tnp2,Tssk2,Spo11,Ddx25,Spag6l,Nphp1,Fscn3,Tssk3,Tbata, Klhl10,H1fnt,Cabyr,Spem1,Ropn1,Tssk6,Bbs4,Catsperd,Slc26a8,Tmf1,Tbpl1,Rimbp3,Dpy19l2,Catsper4 |
| GO BP | GO:0007286 | spermatid development | 6.780898 | Cftr,Hspa2,Prm1,Prm2,Qk,Tnp1,Tnp2,Tssk2,Spo11,Ddx25,Spag6l,Fscn3,Tssk3,Klhl10,H1fnt,Cabyr, Spem1,Ropn1,Tssk6,Bbs4,Catsperd,Slc26a8,Tmf1,Tbpl1,Rimbp3,Dpy19l2,Catsper4 |
| GO BP | GO:0007281 | germ cell development | 6.13202 | Atm,Cftr,Hspa2,Insl3,Prm1,Prm2,Ptn,Qk,Tnp1,Tnp2,Tssk2,Zfp37,Spo11,Ddx25,Spag6l,Fscn3,Tssk3, Klhl10,H1fnt,Cabyr,Paqr8,Spem1,Boll,Ropn1,Tssk6,Bbs4,Catsperd,Slc26a8,Tmf1,Tbpl1,Rimbp3,Dpy19l2,Catsper4,Hfm1 |
| GO BP | GO:0003006 | developmental process involved in reproduction | 4.384473 | Atm,Runx1,Cftr,Col9a3,Crkl,Fkbp4,Bscl2,Hspa2,Hsp90ab1,Ccn1,Il1b,Insl3,Prm1,Prm2,Pten,Ptn,Qk,Tgfb2,Tnp1,Tnp2,Tssk2,Ubb,Zfp37,Esrrb,Spo11,Ddx25,Spag6l,Prdx4,Nphp1,Fscn3,Ybx3,Tssk3,Tbata,Klhl10,Wdr48,H1fnt,Cabyr,Paqr8,Spem1,Ccdc182,Boll,Ropn1,Tssk6,Dach2,Bbs4,Catsperd,Slc26a8,Vash2,Tmf1,Tbpl1,Arid4a,Rimbp3,Dpy19l2,Catsper4,Hfm1,Insl3,Nphp1,Fscn3, Suv39h2,Spdya,Mael,Slc26a8, Catsper1 |
| GO BP | GO:0097722 | sperm motility | 5.48393 | Akap4,Dnah11,Gapdhs,Smcp,Prm3,Tacr3,Tnp2,Spag6l,Spem1,Ropn1,Catsperd,Txndc2,Slc26a8,Catsper1,Tmf1,Catsper4 |
| GO BP | GO:0007339 | binding of sperm to zona pellucida | 4.677972 | Acr,Arsa,Cct7,Cct8,Hspa1l,Smcp,Tcp1,Tnp2,Vdac2 |
| GO BP | GO:0009566 | fertilization | 4.2825 | Acr,Arsa,Cct7,Cct8,Crkl,Hspa1l,Smcp,Tcp1,Tnp2,Vdac2,Clic4,Ybx3,Cacna1h,Klhl10,Wdr48,Iqcf3,Wbp2nl,Mael,Catsper1,Rimbp3, |
| GO BP | GO:0035036 | sperm-egg recognition | 4.165287 | Acr,Arsa,Cct7,Cct8,Hspa1l,Smcp,Tcp1,Tnp2,Vdac2 |
|  |  |  |  |  |
| GO CC | GO:0097223 | sperm part | 9.932616 | Ace,Acr,Akap4,Arsa,Bsg,Csnk2a2,Dnah8,Gapdhs,Hk1,Hsp90ab1,Ldha,Odf1,Slc2a3,Tacr3,Tcp1,Tssk2, Vdac2,Spag6l,Oaz3,Pmfbp1,Iqcf3,Spaca9,Cabyr,Spata18,Tbc1d21,Wbp2nl,Ropn1,Dnajb1,Usp8,Catsperd,Tsga10,Txndc2,Atp8b5,Catsper4 |
| GO CC | GO:0097228 | sperm principal piece | 6.575637 | Akap4,Dnah8,Gapdhs,Hk1,Spag6l,Cabyr,Ropn1,Catsperd,Tsga10,Catsper4 |
| GO CC | GO:0032838 | plasma membrane bounded cell projection cytoplasm | 4.292806 | Dnah11,Dnah8,Htt,Uhmk1,Prkar2a,Spag6l,Gabarapl1,Spata4,Spef1,Ttc30b,Bbs5,Drc3,Wdr35,Iqca, Lca5,Baiap2,Wdpcp,Ttll10,Cep162 |
| GO CC | GO:0005815 | microtubule organizing center | 4.271195 | Atf4,Brca1,Cct8,Dync1h1,Hap1,Htt,Prkcq,Prkar2a,Ptpn20,Rbbp6,Plk2,Tcp1,Tssk2,Nubp2,Clic4,Vps37a,Rab11a,Gsk3b,Ctdp1,Spert,Dctn2,Aunip,Spaca9,4921507P07Rik,Lrrcc1,Ttc30b,Bbs5,Spatc1,Wdr35,Lca5,Cep112,Csnk1a1,Akna,Bbs4,Pkn2,Zmynd10,Hook2,Cep164,Nek9,Cenpj,Cep76,Cdc14a,Ttc12,Leo1, C2cd3,Cep162,Tcp10c,Pifo |
| GO CC | GO:0005813 | centrosome | 3.854755 | Atf4,Brca1,Cct8,Dync1h1,Hap1,Prkar2a,Rbbp6,Plk2,Tcp1,Nubp2,Clic4,Vps37a,Rab11a,Gsk3b,Ctdp1, Dctn2,Aunip,4921507P07Rik,Lrrcc1,Bbs5,Spatc1,Wdr35,Cep112,Csnk1a1,Akna,Bbs4,Pkn2,Zmynd10, Hook2,Cep164,Nek9,Cenpj,Cep76,Cdc14a,Ttc12,Leo1,C2cd3,Cep162 |
| GO CC | GO:0005891 | voltage-gated calcium channel complex | 2.982713 | Cacna2d1,Cacnb1,Hspa2,Cacna1h,Catsperd,Catsper1,Catsper4 |
| GO CC | GO:0099568 | cytoplasmic region | 2.584379 | Macf1,Capza1,Cdh2,Dnah11,Dnah8,Dync1h1,Eef1a1,Epb42,Htt,Uhmk1,Prkar2a,Sptbn1,Stim1,Spag6l,Nsmf,Gabarapl1,4930544G11Rik,Spata4,Spef1,Lancl2,Fryl,Ttc30b,Bbs5,Drc3,Wdr35,Iqca,Lca5,Baiap2,Wdpcp,Rhov,Ttll10,Cep162 |
| GO CC | GO:1902495 | transmembrane transporter complex | 2.23011 | Atp1b3,Cacna2d1,Cacnb1,Calm1,Cftr,Cnga1,Grin2a,Hspa2,Kcnb1,Ryr2,Clic4,Cacna1h,Pex5l,Lrrc8c, Scrib,Catsperd,Chrna5,Ptpa,Catsper1,Akap6,Catsper4,Lrrc8b |
| GO CC | GO:0036064 | ciliary basal body | 1.804505 | Spert,Spaca9,Ttc30b,Bbs5,Wdr35,Lca5,Csnk1a1,Bbs4,Cenpj,Cdc14a,C2cd3,Pifo |
| GO CC | GO:0034703 | cation channel complex | 1.543924 | Cacna2d1,Cacnb1,Calm1,Cnga1,Grin2a,Hspa2,Kcnb1,Ryr2,Cacna1h,Pex5l,Catsperd,Ptpa,Catsper1, Akap6,Catsper4 |
|  |  |  |  |  |
| GO MF | GO:0019901 | protein kinase binding | 3.977697 | Ace,Akap1,Atf4,Cdh2,Cdkn2c,Csk,Dvl1,Eef1a1,Ghr,Grin2a,Gstm1,Gtf2i,Hsp90ab1,Il1rap,Lipe,Nedd9, Pde3b,Plcg1,Prkar2a,Pten,Ptn,Dusp1,Ptprc,Ptprk,Kif20a,Rbbp6,Ryr2,Shc3,Copb2,Rnf138,Gsk3b,Sv2a, Bcl2l14,4930544G11Rik,Dab2ip,Dctn2,Spdya,Mad2l2,Ppme1,Fam83e,Spdye4a,Lims1,Nek9,Cenpj, Rhov,Wnk1,Arhgap33,Dixdc1,Ksr2,Pifo |
| GO MF | GO:0005244 | voltage-gated ion channel activity | 2.375185 | Cacna2d1,Cacnb1,Grin2a,Kcnb1,Kcnu1,Vdac2,Vdac3,Clic4,Cacna1h,Slc17a3,Kcnh8,Kcnk9,Catsper1, Catsper4,Nalcn,Kcnh4, Cftr,Cnga1,Grin2a,Hpn, Kcnu1,Rasa3,Ryr2, Pex5l, Chrna5, Stim1, Lrrc8c, Slc26a8, Lrrc8b, |
| GO MF | GO:0022838 | substrate-specific channel activity | 1.984003 | Cacna2d1,Cacnb1,Cftr,Cnga1,Grin2a,Hpn,Kcnb1,Kcnu1,Rasa3,Ryr2,Stim1,Vdac2,Vdac3,Clic4,Cacna1h,Pex5l,Lrrc8c,Slc17a3,Chrna5,Kcnh8,Kcnk9,Slc26a8,Catsper1,Catsper4,Nalcn,Kcnh4,Lrrc8b |
| GO MF | GO:0015267 | channel activity | 1.934522 | Cacna2d1,Cacnb1,Cftr,Cnga1,Grin2a,Hpn,Kcnb1,Kcnu1,Rasa3,Ryr2,Stim1,Vdac2,Vdac3,Clic4,Cacna1h,Pex5l,Lrrc8c,Slc17a3,Chrna5,Gjc3,Kcnh8,Kcnk9,Slc26a8,Catsper1,Catsper4,Nalcn,Kcnh4,Lrrc8b |
| GO MF | GO:0022803 | passive transmembrane transporter activity | 1.934522 | Cacna2d1,Cacnb1,Cftr,Cnga1,Grin2a,Hpn,Kcnb1,Kcnu1,Rasa3,Ryr2,Stim1,Vdac2,Vdac3,Clic4,Cacna1h,Pex5l,Lrrc8c,Slc17a3,Chrna5,Gjc3,Kcnh8,Kcnk9,Slc26a8,Catsper1,Catsper4,Nalcn,Kcnh4,Lrrc8b |
| GO MF | GO:0022843 | voltage-gated cation channel activity | 1.565139 | Cacna2d1,Cacnb1,Grin2a,Kcnb1,Kcnu1,Cacna1h,Kcnh8,Kcnk9,Catsper1,Catsper4,Kcnh4 |
| GO MF | GO:0070064 | proline-rich region binding | 1.367954 | Csk,Nedd4,Baiap2 |
| GO MF | GO:0005261 | cation channel activity | 1.364565 | Cacna2d1,Cacnb1,Cnga1,Grin2a,Hpn,Kcnb1,Kcnu1,Rasa3,Ryr2,Stim1,Cacna1h,Pex5l,Chrna5,Kcnh8, Kcnk9,Catsper1,Catsper4,Nalcn,Kcnh4 |
| GO MF | GO:0005245 | voltage-gated calcium channel activity | 1.325073 | Cacna2d1,Cacnb1,Cacna1h,Catsper1,Catsper4 |
| GO MF | GO:0005262 | calcium channel activity | 1.306753 | Cacna2d1,Cacnb1,Grin2a,Rasa3,Ryr2,Stim1,Cacna1h,Catsper1,Catsper4 |
