# Supplementary figures and images for: Role of Phosphorylated Gonadotropin-Regulated Testicular RNA Helicase (GRTH/DDX25) in the Regulation of Germ Cell Specific mRNAs in Chromatoid Bodies During Spermatogenesis
Source: Front Cell Dev Biol. 2020 Dec 23;8:580019. doi: 10.3389/fcell.2020.580019 (PMC7786181; doi:10.3389/fcell.2020.580019)

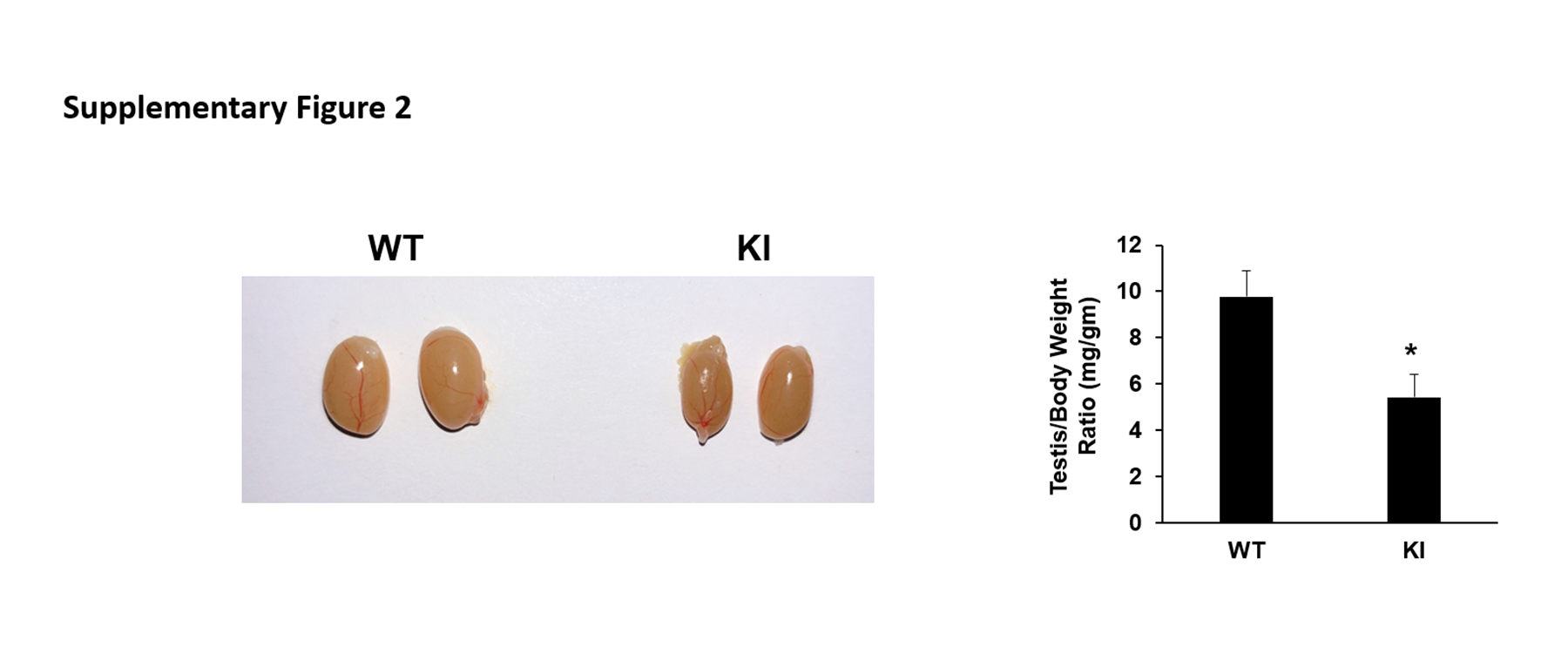

Supplement: Supplementary Table 1 — List of primers used for genotyping and validation of differentially enriched transcripts using qRT-PCR. [file Data_Sheet_1.zip › Supplementary files/Supplementary Figure 2.tif]

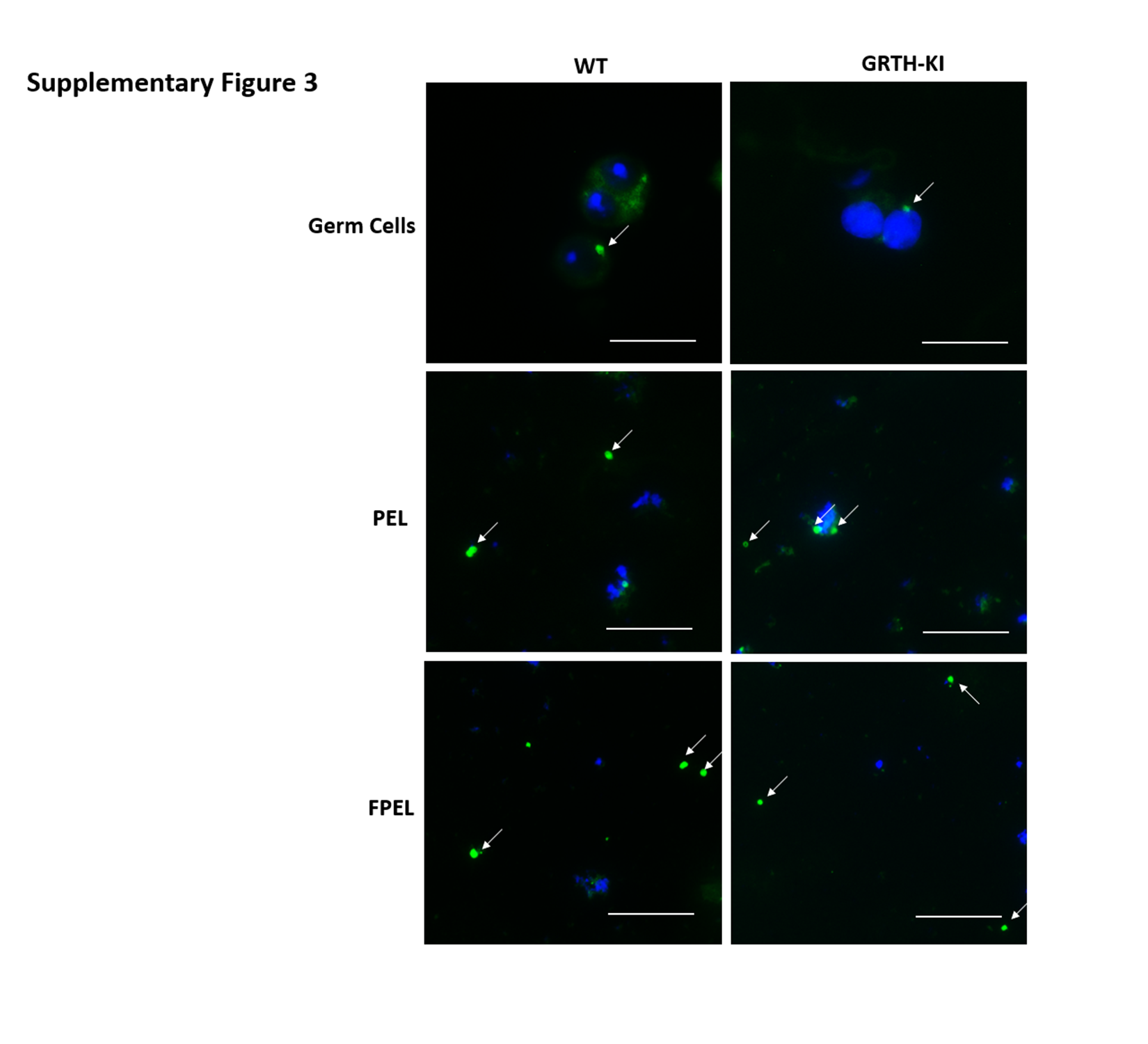

Supplement: Supplementary Table 1 — List of primers used for genotyping and validation of differentially enriched transcripts using qRT-PCR. [file Data_Sheet_1.zip › Supplementary files/Supplementary Figure 3.tif]

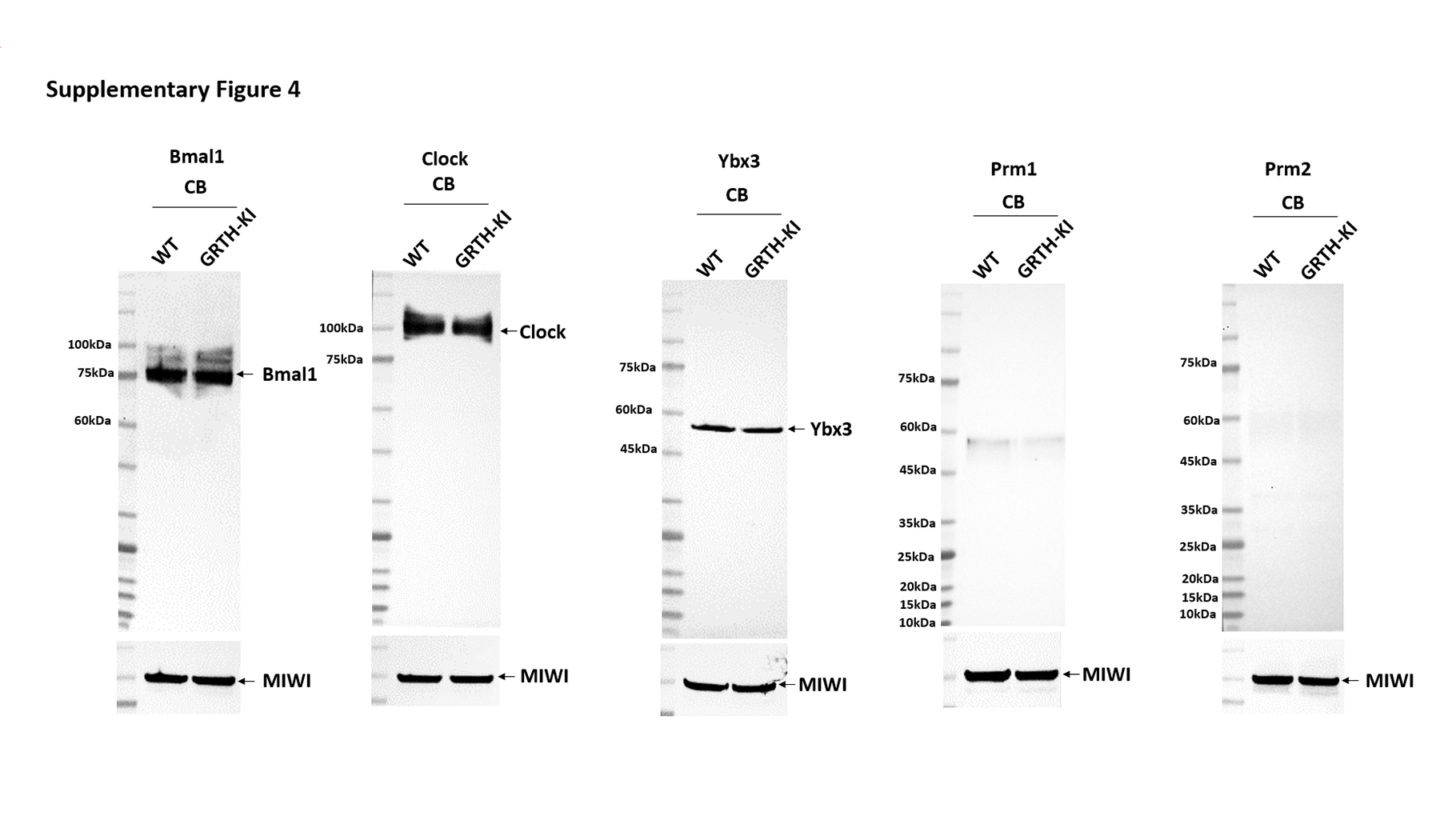

Supplement: Supplementary Table 1 — List of primers used for genotyping and validation of differentially enriched transcripts using qRT-PCR. [file Data_Sheet_1.zip › Supplementary files/Supplementary Figure 4.tif]

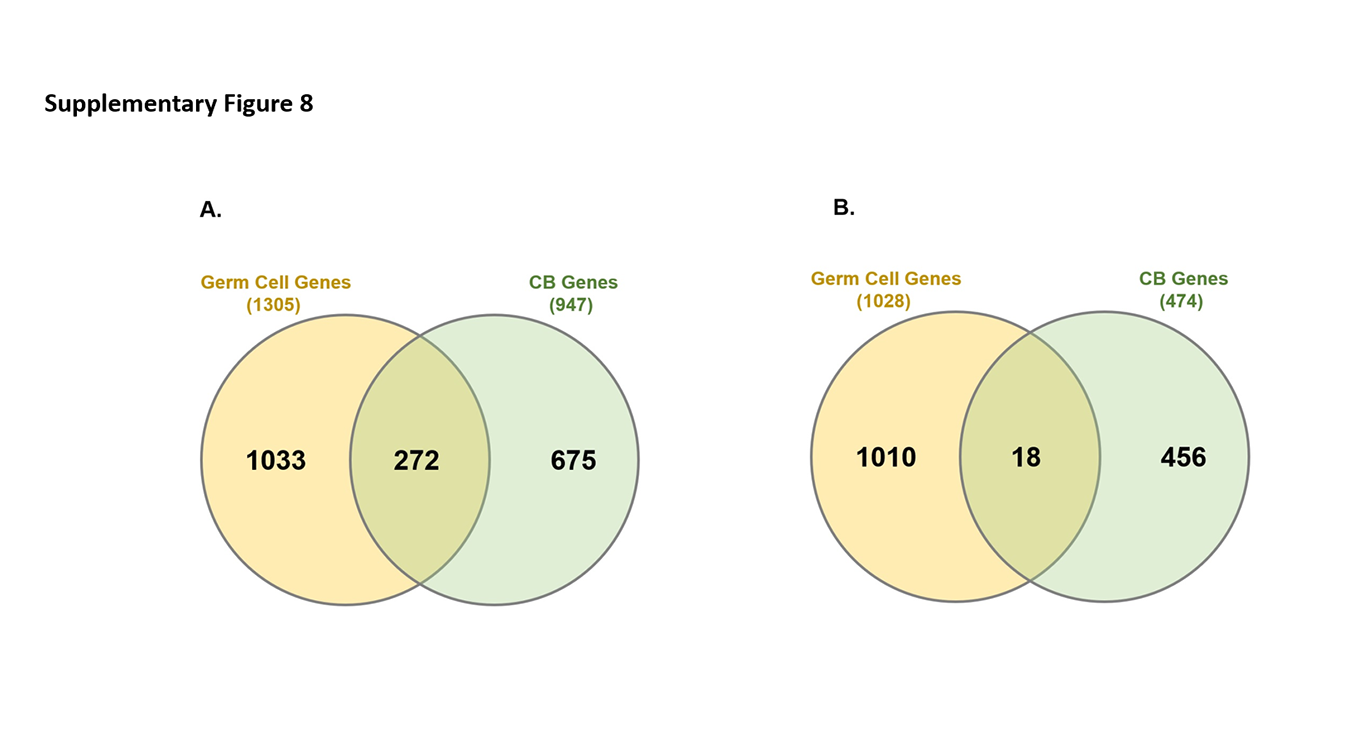

Supplement: Supplementary Table 1 — List of primers used for genotyping and validation of differentially enriched transcripts using qRT-PCR. [file Data_Sheet_1.zip › Supplementary files/Supplementary Figure 8.tif]

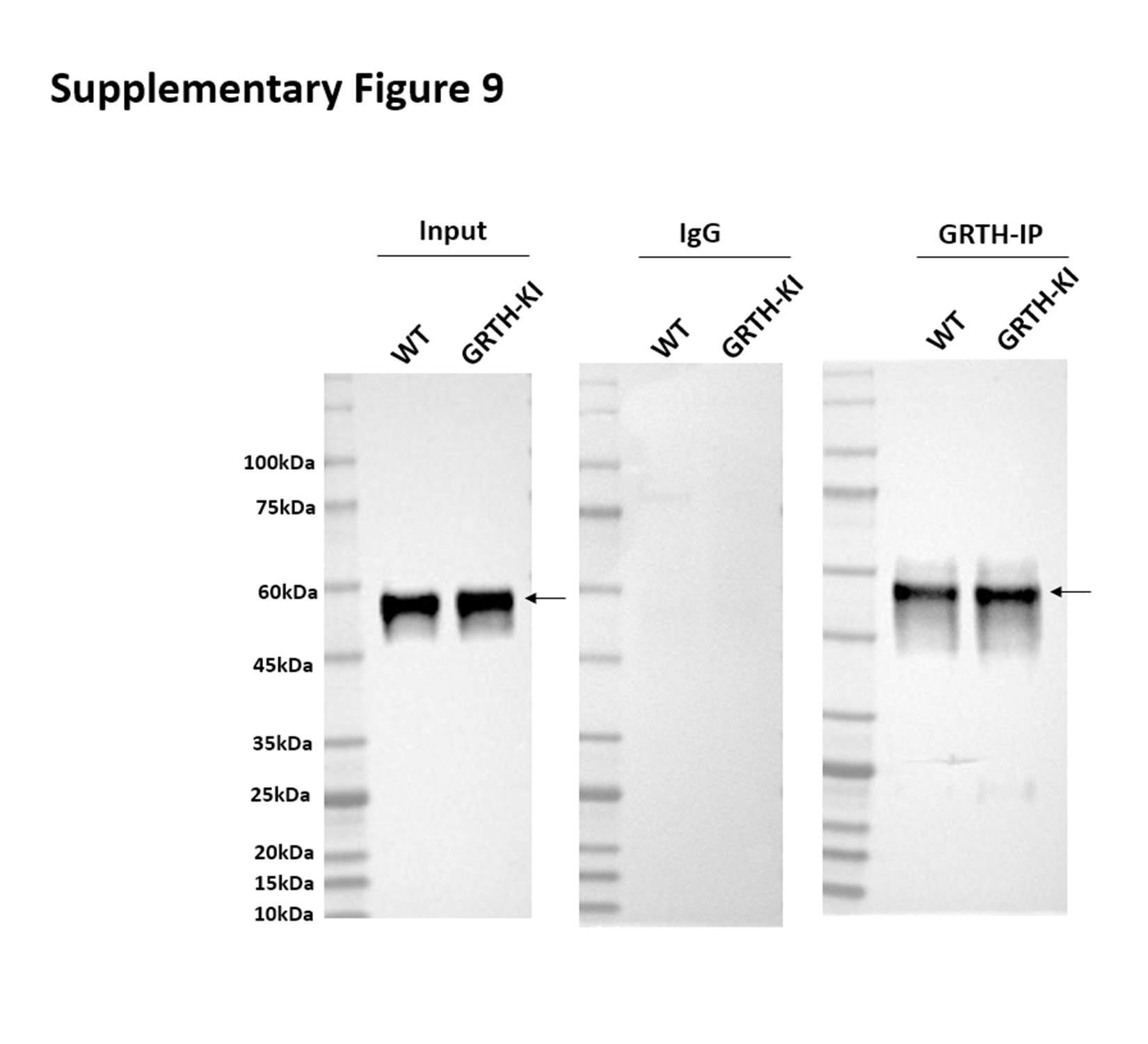

Supplement: Supplementary Table 1 — List of primers used for genotyping and validation of differentially enriched transcripts using qRT-PCR. [file Data_Sheet_1.zip › Supplementary files/Supplementary Figure 9.tif]
